# Author Correction: A sedimentary ancient DNA perspective on human and carnivore persistence through the Late Pleistocene in El Mirón Cave, Spain

**DOI:** 10.1038/s41467-025-56198-x

**Published:** 2025-01-17

**Authors:** Pere Gelabert, Victoria Oberreiter, Lawrence Guy Straus, Manuel Ramón González Morales, Susanna Sawyer, Ana B. Marín-Arroyo, Jeanne Marie Geiling, Florian Exler, Florian Brueck, Stefan Franz, Fernanda Tenorio Cano, Sophie Szedlacsek, Evelyn Zelger, Michelle Hämmerle, Brina Zagorc, Alejandro Llanos-Lizcano, Olivia Cheronet, José-Miguel Tejero, Thomas Rattei, Stephan M. Kraemer, Ron Pinhasi

**Affiliations:** 1 Department of Evolutionary Anthropology, University of Vienna, Vienna, Austria; 2 Human Evolution and Archeological Sciences (HEAS), University of Vienna, Vienna, Austria; 3 Department of Anthropology, University of New Mexico, Albuquerque, NM USA; 4 Grupo I+D+i EvoAdapta, Departamento de Ciencias Históricas, Universidad de Cantabria, Santander, Spain; 5 Instituto Internacional de Investigaciones Prehistóricas de Cantabria (Universidad de Cantabria, Gobierno de Cantabria, Santander), Santander, Spain; 6 Department of Environmental Geosciences, Centre for Microbiology and Environmental Systems Science, University of Vienna, Vienna, Austria; 7 Department of Botany and Biodiversity Research, University of Vienna, Vienna, Austria; 8 Facultad de Química y Farmacia, Universidad del Atlántico, Barranquilla, Colombia; 9 Seminari d’Estudis i Recerques Prehistòriques (SERP), University of Barcelona, Barcelona, Spain; 10 Division of Computational Systems Biology, Centre for Microbiology and Environmental Systems Science, University of Vienna, Vienna, Austria

**Keywords:** Evolutionary biology, Population genetics

Correction to: *Nature Communications* 10.1038/s41467-024-55740-7, published online 02 January 2025

In this article the following sentence was omitted from the Acknowledgements section, ‘Open access funding provided by University of Vienna’. The original article has been corrected.

