# Supplementary material for: A sedimentary ancient DNA perspective on human and carnivore persistence through the Late Pleistocene in El Mirón Cave, Spain
Source: Nat Commun. 2025 Jan 2;16:107. doi: 10.1038/s41467-024-55740-7 (PMC11696082; doi:10.1038/s41467-024-55740-7)
Supplement: Supplementary file 1 — Supplementary Information [file 41467_2024_55740_MOESM1_ESM.pdf]

Supplementary Information

Supplementary Note 1: Site description

Supplementary Note 2: Detection of Inhibition in the DNA extracts

Supplementary Note 3: The Classification of sedaDNA at the species level

Supplementary Note 4: Individual genomes and phylogenies

Supplementary References

Supplementary Note 1: Site Description

1. El Mirón cave

The archaeological site of El Mirón is located on the eastern edge of the northern Spanish region of Cantabria, about 20 km from the present Bay of Biscay shore. The site is 260 m a.s.l. on a mountainside dominating the broad upper Asón River valley, which here is at c. 100 m a.s.l. It is a limestone cave excavated since 1996 under the direction of L.G. Straus and M.R. González Morales, and recently I. Gutiérrez Zugasti and D. Cuenca Solana [1,2]. The cave has a prominent mouth and a spacious, mostly dry, sunlit vestibule (30 m deep to 16 m wide and 13 to 20 m high) (Supplementary Figure 1A). In contrast, the 100 m deep inner cave is narrow, low-ceilinged and completely dark.

El Mirón was occupied by humans from the late Middle Palaeolithic until modern times, including much of the Upper Palaeolithic (Gravettian, Solutrean, Magdalenian and the Epipalaeolithic Azilian period, as well as the Neolithic, Chalcolithic and Bronze Age). It is one of the best-dated late Quaternary sites of the Cantabrian Region, with 101 radiocarbon assays, major assemblages of artefacts and faunal remains, and numerous palaeoenvironmental studies [3–6]. With excellent preservation of osseous material and large-scale excavations, the site has yielded numerous human remains, notably those of the Lower Magdalenian "Red Lady" burial (18898-18747 cal BP) [7]. The genome of this individual has played a key role in defining the genetic characteristics of the Magdalenian population of Europe [8]. Recent genetic research has defined the Magdalenian population represented by the human buried remains called "Red Lady" as the result of the Gravettian-like population that survived the LGM during the Solutrean (represented by the genome of Fournol) plus an incoming gene flow represented by the genome of Arene-Candide 16. The genetic ancestry of the "Red Lady" can be modelled with these two sources [9]. Other human remains (loose teeth) have been found in other Lower Magdalenian levels. Surface deposits have been disturbed pertaining in part to the Chalcolithic and Bronze Age.

The archaeological evidence found at the lowermost sequence of El Mirón (Mousterian, Early Upper Palaeolithic: >46-28 cal kya BP), excavated below a series of Solutrean levels

only in a $2m^2$ *sondage* (squares W-X10) dug below the base of a large looters' pit at the rear of the cave vestibule, but tied in stratigraphically to the overlying sequence of Initial, Lower, Middle and Upper Magdalenian levels in the contiguous area not affected by looting, indicates an alternating occupation of the cave by humans and carnivores. Human visits to the cave during the Mousterian and early Upper Palaeolithic were sporadic, ephemeral, and probably seasonal, with limited evidence showing that occupations took place in winter or spring based on the ages of ibex individuals [1].

The Lower Magdalenian levels (c. 20-18 cal kya), in particular, are extraordinarily rich in artefacts, including lithic and osseous manufacturing debris and finished products, namely domestic tools, weapons, personal ornaments, and works of portable art (some both characteristic of the regional archaeological culture at this time and others indicative of social connections with human groups in southern France), and anthropogenic features items such as hearths, pits and a possible wall. The ritual human burial documented during this period at El Mirón is associated with (indeed marked by) non-local red ochre and rock art and is unique for this period in Iberia. The classic Cantabrian Lower Magdalenian horizon was preceded in El Mirón by a series of rich Initial Magdalenian levels (c. 21-20 cal kya) extremely rare for Iberia. They overlie Solutrean levels (c. 25-21 cal kya) that attest to several brief visits to this montane site (possibly from base camps near the now-inundated LGM coast) by hunting parties who left behind numerous fragmentary stone projectile points and elements of personal adornment. The pre-LGM humans (presumably *Neanderthal* and *or* Anatomically Modern Humans (AMH)) who inhabited the cave in preceding times (> 47, and up to c. 31.5 cal kya) were even more ephemeral. All these archaeological levels (Mousterian, Early Upper Palaeolithic, Gravettian, Solutrean and Initial Magdalenian) in El Mirón provide a unique archive for high-resolution studies on the alternating patterns of human and animal presence within Pleistocene archaeological cave sites[10]. The genetic study of a Magdalenian burial (The Red Lady) was crucial to reveal the arrival of Villabruna genes into the former refugium of Iberia during the Magdalenian[8].

2. Sedimentology and sample stratigraphic and taphonomic context

The sedimentology of El Mirón was published by the late W.R. Farrand in a *Geoarchaeology* article and in a chapter of the El Mirón monograph [11,12]. The performed analyses include study on the particle size, the roudness, Calcium Carbonate and Organic Matter, Lithology and Clay Mineralogy.

Levels 130-121 are a series of colluvial, sandy loam deposits. Among these are finer grain levels, others more gravelly, with varying amounts of alluvial pebbles and cobbles and limestone rocks spalled rocks from the cave walls and ceiling, generally yellowish-brown or brownish-yellow, but a few browner or olive-brown. The clay-sized sediment fraction ranges from 30 to 50%, the rest is coarse fraction. There are no visible changes in this ratio through the sondage.

The pH ranges between 8.67-8.08, except Levels 125-123, which vary between 7.74-7.91. These levels are generally low in organic matter, although some Solutrean levels are richer therein [11]. These basal levels (130-121) slope down (westward) toward the cave mouth, c. 15-20°. Their faunal and cultural remains were deposited atop the eroded face of alluvial sediments that fill the inner cave (Supplementary Figure 1), which had been voided out of the cave vestibule by running water. Hence, these levels are at the interface between the alluvial in-filling of the inner cave and the vestibule, which had later been re-filled with highly anthropogenic Upper Palaeolithic and post-Palaeolithic deposits (coarse material). Levels 121-130 are described by Farrand [11] as "colluvial terrace" material. The Solutrean levels 121-127 are relatively rather thin, very clearly stratified and without macroscopic observations of diagenesis, with concentrated bones and stone artefacts. The bones in even levels 128-130 are well-preserved. There is no evidence of gullying or significant water disturbance, but the materials are quite scarce and dispersed through quite thick levels (especially 130)[13] reveal that the bones (including ones of very small animals—birds, lagomorphs) are in fine condition and showing not signs of transport (i.e., surfaces not altered by running water or other diagenetic processes, etc.) in these levels. So, neither the sedimentology nor the taphonomy would lead us to conclude disturbance. We do not have any evidence indicating that the deposits, particularly those from the Solutrean and Initial Magdalenian levels, are in a secondary

position. Similar studies at other Cantabrian archaeological sites have revealed secondary depositions and disturbances. For instance, in La Cueva de El Pendo[14,15], a taphonomic reappraisal of the sequences suggests that the interstratifications do not correspond to a primary archaeological sequence but may result from post-depositional processes. A similar situation has been reported at La Güelga cave, where micromorphological observations and new radiocarbon dating strongly suggest that the few presumably Châtelperronian finds were transported [16].

Description of the sampled levels

Level 130, which is a massive (> 1 m), undifferentiated horizon whose base was not reached, yielded 115 stone artefacts (2 denticulates, a burin, a flake core, 110 items of debitage and a hammerstone) that were not concentrated either horizontally or vertically. Undated, 35-50 cm-thick Level 129 produced only 14 items of debitage—including blades and bladelets [10]. Level 128—30-50 cm-thick—is artifactually much richer (550 items of debitage, including 66 blades and bladelets, a bladelet core and a mixed core, plus 23 retouched tools including many classic "Upper Palaeolithic" types none of which, however, is diagnostic of the Gravettian despite the 31-32 cal ky BP radiocarbon age[17] [13].

Levels 127-122 are generally 10-20 cm thick each and some are discontinuous in the area excavated. All but one yielded Solutrean points (laurel, willow, shouldered or unifacial types), while artefact-poor Level 121 had none, although overlying, disturbed "Level" 120 (hard-packed, mixed sediments at the very base of the looter pit) had a shouldered point. Radiocarbon dates from Levels 127, 126, 125, 122 and 121 all fall within the Solutrean period. In total, these levels yielded 13,673 items of lithic debitage (ranging between 451-3739 per level) and 223 retouched tools/weapons (between 10-55 per level), and several perforated animal teeth. bones and mollusc shells, small numbers of bone/antler artefacts (points, needles, awls, and blanks) and a possibly engraved antler tine [3] [14].

None of these basal levels sampled in the deep pit (130-121) had apparent hearths, concentrations of charcoal, or black staining from fires. Human occupations---at least in this area at the rear of the cave vestibule—were relatively minor and probably short, although the Solutrean occupations—possibly by hunting parties—were more significant

than the visits during late Middle Palaeolithic, possibly Early Upper Palaeolithic and "Gravettian" periods whose traces are quite ephemeral.

Atop this sequence of light-colour, organic- and artefact-poor levels, there lies the long, artifactually and faunally extraordinarily rich, dark (variegated, but often blackish-brown, organic-rich, loam, with angular, white limestone fragments and cobbles) series of early (Initial and Lower) Magdalenian levels. The one sampled for this study, Level 119.2 in square V10, pertains to the Initial Magdalenian. There is a clear break (unconformity) between Level 121—the uppermost Solutrean—and the c. 20-30 cm-thick Level 119-119.3 series. This 119-119.3 series yielded 62,087 items of lithic debitage and 639 retouched tools/weapon elements. Osseous artefacts—notably large, round-section points (*sagaies*)-- are relatively numerous and often engraved. There are also bone needles, blanks, and—in Level 19.2—a perforated, schist-like plaque with the engraving of a horse head. The contrasts between this stratigraphic unit (and the other overlying Initial and Lower Magdalenian levels) and the underlying Mousterian, Early Upper Palaeolithic and even Solutrean levels in terms of sediment colour, organic matter, faunal, charcoal and artefact contents are stark. Human occupations of the cave vestibule during the Initial Magdalenian were massive, long-duration, repeated (spring and fall) and multi-functional, as judged by the wide variety of artefacts (used for diverse manufacture/maintenance activities, hunting, decoration, etc.) and evidence of fires [18].

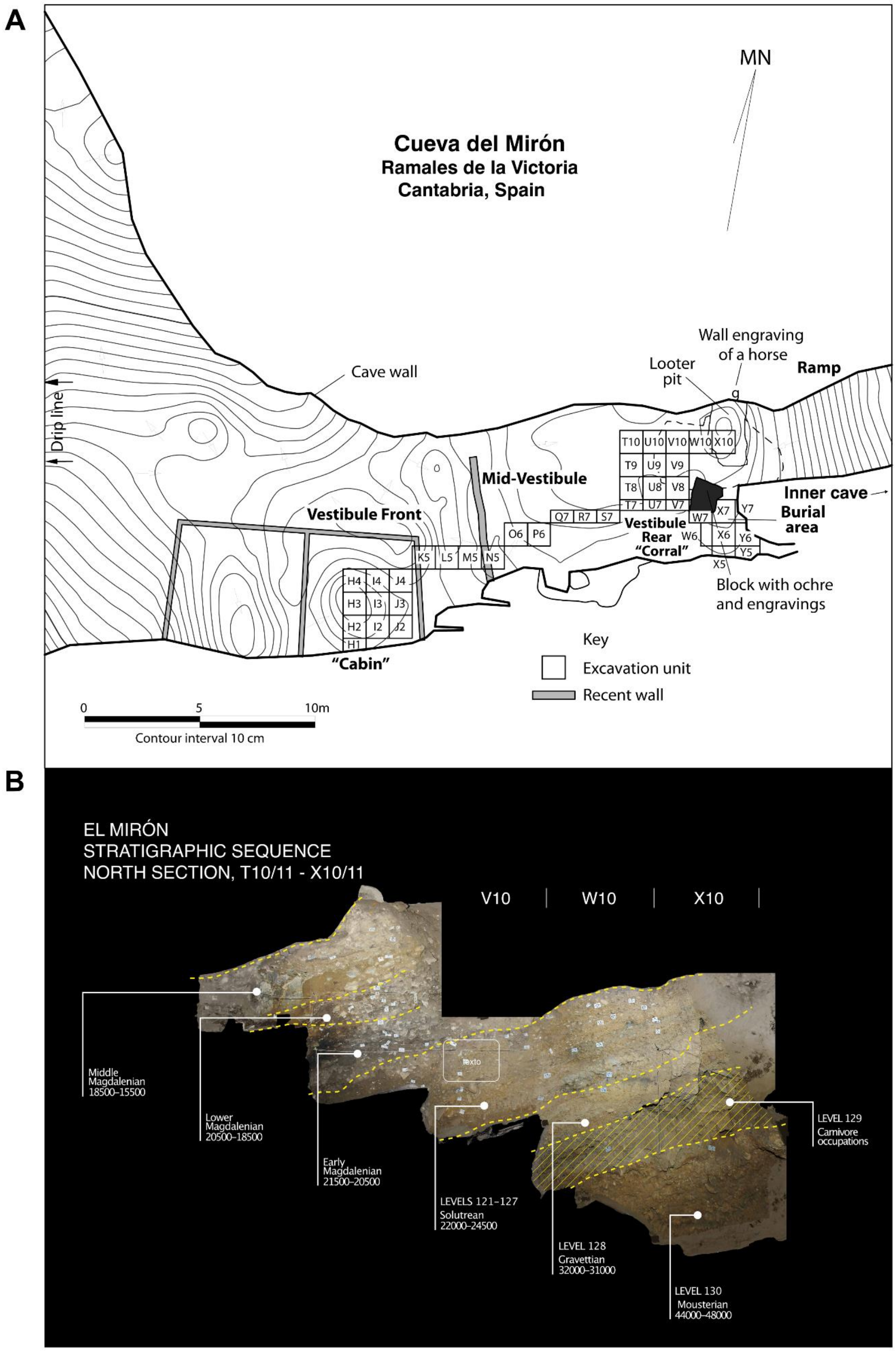

Supplementary Figure 1: A) Plan of El Mirón Cave (by L.G. Straus and R.L. Stauber, based on cave topography by E. Torres). B) Stratigraphic sequence of the North Section with corresponding periods. (photos and montage by M.R. González Morales).

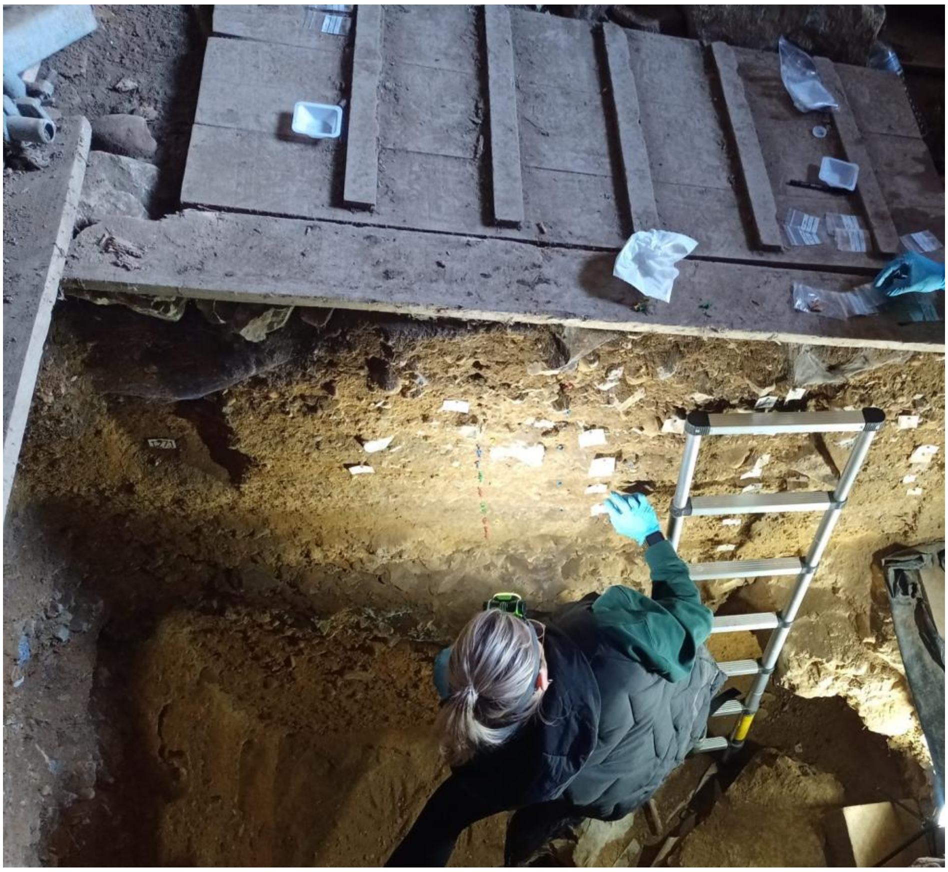

Supplementary Figure 2: Sampling procedure on the rear vestibule of the Cave, February 2023.

3. Archaeozoological record at El Mirón

The Middle (Level 130, ≥46 cal ky BP), and/or Early Upper Palaeolithic (Levels 129-128, Level 128: 31-32 cal ky BP), Solutrean (Levels 127-121: c. 24.5-21.5 cal ky BP) and Initial Magdalenian (Levels 119-119.2: c.21-20.5 cal ky BP) mammalian faunas from El Mirón (which have been analysed taxonomically and taphonomically) principally include red deer, and ibex, in addition to chamois, roe deer, leporids, plus traces of horse, mammoth, wolf, cave lion, and bear in some of the levels [13,19]. The summary of all the fauna identifications in levels 130-119.2 is presented in Supplementary Data 7 and Supplementary Table 1. The small amount of carnivore remains identified is compatible with the roaming activities of these taxa that did not leave evident remains. The archaeological evidence found at the lower sequence of El Mirón (Mousterian, Early Upper Paleolithic: >46-28 cal kya BP), clearly indicates an alternating occupation of the cave by humans and carnivores. Human visits to the cave during the Mousterian and early Upper Palaeolithic were sporadic, ephemeral, and probably seasonal, with limited evidence showing that visit occupations took place in winter or spring based on the ages of ibex individuals [1]. No hominin remains have been discovered in the deep but small (2x1 m) pit (squares W-X/10) that samples these basal levels or in the archaeologically richer overlying sequence of Solutrean and Initial Magdalenian levels (c. 24.5-20.5 cal kya BP) excavated over a somewhat larger area also at the vestibule rear (1m^2 more for the Solutrean levels in square V10); 2 m^2 for the Initial Magdalenian ones in squares V10 and V8)). Despite the evidence of albeit scanty human occupation, no human DNA has been recovered from the Mousterian and EUP levels.

Supplementary Table 1 : Ungulate & Leporid remains from the Middle and Palaeolithic, Early Upper Palaeolithic, Solutrean and Initial Magdalenian Levels in El Mirón Cave studied in this project (NISP/MNI).

Level	*Equus* sp.	Bos/Bison sp.	*Cervus elaphus*	*Capreolus capreolus*	*Capra pyren aica*	*Rupicapra pyrenaica*	*Lepus* sp.
119.2	2/1	9/2	151/8	2/1	122/1 2	12/1	
121			107/4		77/4	22/33	1/1
122		2/1	106/3		107/5	19/2	1/1
123					12/1	2/1	
124	1/1		59/3		59/4	10/3	
125			73/4		97/4	5/2	1/1
126			14/2		8/3	9/1	1/1
127			20/3		26/2		
128			44/3		86/3	10/1	64/6
129			8/2		26/2	1/1	15/2
130	2/1		15/2		54/3	10/2	6/2

Supplementary Note 2: Detection of Inhibition in the DNA extracts

To evaluate the inhibition potential of each extract, 5 µL of an extract diluted in EBT by a factor of 5 (equivalent to 1 µL of pure extract) was added to a PCR reaction designed to amplify the CL104 template oligo (see Supplementary Table 2). The inhibition potential was determined by measuring the concentration of amplified DNA using the Qubit 1x dsDNA HS Assay Kit and comparing it to a control that contained EBT instead of the extract. The efficiency of the PCR reaction is calculated based on the concentration values obtained for the control (4,47±0,10 ng/µL; n=3) (Supplementary Data 4, Supplementary Figure 3).

Supplementary Table 2: Inhibition PCR conditions used in the experiment.

	Volume / µL
10X Standard Taq Reaction Buffer	2.5
dNTPs (10 mM)	0.5
CL107 (5 µM) [42]	1
CL108 (5 µM) [42]	1
CL 104 (0,4 nM) [42]	1
Taq DNA Polymerase	0.125
Nuclease-free water	13.875
Diluted extract	5
Total	25

Step	Temp	Time
Initital denaturation	95°C	30 s
35 cycles	95°C	30 s
	52°C	30 s
	68°C	30s
Final Extension	68°C	5 min
Hold	4°C	

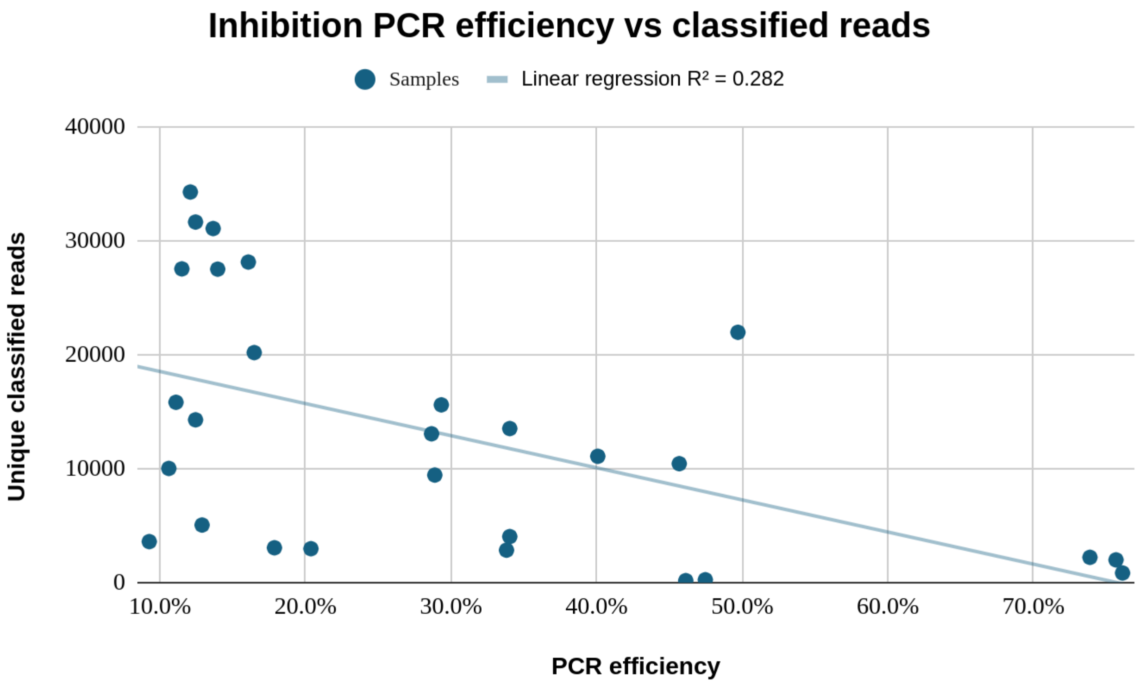

Supplementary Figure 3: Inhibition PCR efficiency vs classified sequencing reads.

We tested the presence of a linear regression (using thePCR efficieny and the sequencing reads) and observed that the R^2 value is 0.282 (n=31). This indicates that approximately 28.2% of the variance in the dependent variable (classified reads) is explained by the independent variable (Efficiency). The *p-value* is 0.00127, showing statistical significance.

Interpretation: Our regression analysis indicates that inhibition is higher in younger samples compared to older ones, with statistical significance. The lower levels yielded fewer classified reads, suggesting that organic material (DNA and inhibitory substances) is less preserved in the older levels. Therefore, the reduced number of classified reads obtained for level 130 is attributed to low DNA preservation rather than inhibition.

Supplementary Note 3: The Classification of sedaDNA at the species level

1. Classification process

To classify the recovered eukaryotic mtDNA reads at a family-order level, we used the euka pipeline[29]. All 32 samples showed the presence of mammalian aDNA. The average number of reads classified per sample using euka is 41,701. The data was classified by euka into 26 taxa, including three genera, 12 families, and 11 other taxonomic groups from suprafamily to order (Supplementary Data 2). The number of recovered DNA reads varied for each archaeological level and sample (Supplementary Data 2), ranging from 2,641 to 124,874 classified unique reads. While we retrieved aDNA from all the levels, there are vertical differences in the section: the average classified amount of reads per sample from the Mousterian (level 130) is 10,518 classified unique reads. This value is five times higher in the Solutrean levels, with an average of 53,745 classified unique reads (Supplementary Data 2). This classification process enabled us to distinguish the animal families present at El Mirón and prepare the data for the single-taxa analyses (Supplementary Data 2). We do not use this data to assess the relative presence of taxa in the samples due to classification limitations and the inability to specifically validate these reads.

We then refined the euka identifications with BLASTn[31], and family-order level reads were classified into genera and species using the LCA algorithm implemented in MEGAN[32]. The following taxonomical levels were identified in euka and resolved at the genus or species level through the BLASTn process:

The reads are classified at the ***Bovidae*** family level and were classified into genera. We determined the presence of genera: *Bos, Bison, Ovis, Capra and Rupicapra.* Each of the reads aligning to these genera was later attributed to *Bos sp. (most likely Bos primigenius), Bison bonasus, Ovis aries, Capra pyrenaica, and Rupicapra pyrenaica.* In the case of *Rupicapra* and *Capra*, as the classification through BLASTn was not conclusive, we agreed on the assignations based on the phylogeny, which shows that the reads belong to the diversity of *Capra pyrenaica* and *Rupicapra pyrenaica.* (Supplementary Figure 34).

The reads classified at the ***Canidae*** family level were later classified in genera. We determined the presence of the following genera: *Cuon, Canis and Vulpes.* Each of the reads aligning to these genera was later attributed to *Cuon alpinus, Canis lupus* and *Vulpes vulpes. Cuon alpinus* is an uncommon taxa in Iberia, with only some identifications in the archaeofauna register [43]. *Vulpes vulpes* and *Canis lupus* are however much more common, both have already been detected in El Mirón [10].

The reads belonging to ***Carnivora*** yielded only *Crocuta* identifications; all the reads classified as *Crocuta* genus were determined to belong to *Crocuta crocuta.*

The reads belonging to the ***Cervidae*** family are later classified in genera. We only identified reads belonging to the genus *Cervus.* All reads classified as *Cervus* were identified as *Cervus elaphus.*

No further attributions were possible for reads classified at the ***Charadriiformes*** level.

The reads belonging to the ***Corvoidea*** family are classified in genera. We successfully identified reads belonging to the genus *Pyrrhocorax.* All reads classified as *Pyrrhocorax* were identified as *Pyrrhocorax graculus, which is* common in Pleistocene sites in Iberia[44] although not identified in El Mirón remains yet.

The reads belonging to ***Corvus*** are classified into genera, and only the reads belonging to the genus *Corvus are classified.* All reads classified as *Corvus* were identified as *Corvus corax. Corvus corax* has been identified as a human exploited animal during the Pleistocene in Iberia [45].

The reads belonging to the ***Soricidae*** were later classified into genera. We successfully identified reads belonging to the genus *Sorex* and attributed them to *Sorex araneus.*

The reads belonging to the ***Suina*** level were later classified into the genera *Sus*, and those belonging to the genera *Sus* were classified as *Sus scrofa.*

The reads belonging to the ***Ursidae*** family were later classified in genera. We successfully identified reads belonging to the genus *Ursus.* All the reads belonging to *Ursus* were determined to belong to *Ursus arctos.*

The reads belonging to the ***Equidae*** family were later classified in genera, we determined the presence of the genus *Equus*. All the reads belonging to the genus *Equus* were determined to belong to *Equus* sp.

The reads belonging to the ***Lepus*** genus were determined to belong to *Lepus* sp. A common species in El Mirón fauna.

The reads belonging to the ***Passeroidea*** parvorder were not sufficient for further classifications.

The reads belonging to the ***Eulipotyphla*** order were later classified into genera, we determined the presence of the genus *Talpa*. All the reads belonging to the genus *Talpa* were classified as *Talpa europaea.*

The reads belonging to the ***Felidae*** family were later classified into genera. We determined the presence of the following genera: *Panthera, Felis, and Lynx.* The reads aligning to these genera were later attributed to *Panthera pardus*, *Felis catus,* and *Lynx pardinus.* The iberian Lynx was common in Iberia during the Pleistocene [46]

The reads belonging to the ***Neognathae*** infraclass were later classified into genera, and we determined the presence of the following genera: *Columba* and *Falco*. The reads aligning to these genera was later attributed to *Columba livia* and *Falco* sp. *Columba livia* has also descrived as a human-exploited ressource during the Pleistocene in Iberia [47]

The reads belonging to the ***Pecora*** infraorder were later classified into genera, and we determined the presence of the following genera: *Capreolus* and *Rangifer. The* reads aligning to these genera was later attributed to *Capreolus capreolus* and *Rangifer tarandus. Rangifer tarandus* is rare in the North of Iberia [48]. *Caperolus capreolus* is much more common [49].

The reads belonging to the ***Muridae*** family were not sufficient for further classifications.

The reads belonging to the ***Glires*** order were not sufficient for further classifications.

The reads belonging to the ***Galliformes*** *o*rder were too few for further classifications.

The reads belonging to the ***Microtus*** genus were classified as *Microtus sp.*

The reads belonging to the ***Cricetidae*** family were not sufficient for further classifications.

All the reads belonging to the genus ***Mustela*** were classified as *Mustela nivalis.*

All reads belonging to order ***Proboscidea*** were classified as *Mammuthus primigenius.* Wholly Mammoths have been identified in more than 20 sites from the MIS 3-2 in Iberia [50]

All reads belonging to order ***Perissodactyla*** were classified as *Coelodonta antiquitatis.* Wholly rhino is not common but has been identified in several sites of the North of Iberia. With documented presence until 20 kya [51].

All the reads ***Homininae*** subfamily were classified as *Homo sapiens.*

The unique classified reads were finally aligned to their reference sequence (as mentioned before either the proper identified sequence or the selected sequence based on literature) using BWA aln[33]. This process enabled a strict classification and the discarding of spurious assignments as each sequencing read was placed in a single taxon as well as removing PCR duplicates. To claim a specific species/genus identification in a given sample, we set a cut-off value of 100 reads, an average read length below 75 bp, and a 20% deamination signal (C>T changes to the reference) in the read ends.

This process successfully identified 31 mammal and avian species/genera (Figure 2, Supplementary Data 3). Multiple samples yielded more than 1,000 unique reads assigned to a specific mammalian species. The read length distributions of mapped reads show a general average read length between 50 and 60 bp. However, some taxa, such as *O. aries,*

do not have short reads, pointing towards modern origin. Following these observations (the read-end deamination and length estimates), we determined the presence of modern contaminants in some samples. We detected the presence of modern *Ovis aries* in samples El Miron_13, Mirón_1, El Miron_2, El Miron_14, El Miron_16 and especially in El Miron_3 where 9,460 unique *O. aries* reads were identified as modern. *Felis catus* DNA was identified in sample El Miron_3 (Supplementary Data 3). In addition, the read length distribution of *M. arvalis* (Supplementary Figure 4) shows the presence of elevated long reads despite a clear deamination signal (Supplementary Data 3), we decided to not study these reads due to the uncongruent length. Therefore, *Ovis' and Felis'* and *Microtus's* identifications were removed from the results, and these taxa were not further analysed. Following this identification we also removed the *Capra* sp. from ElMiron_3 from the analyses because of damage (10% lower than the average 55%) and long read distribution (average of 75 vs average arround 60 for the rest). The origin of the high numbers of *O. aries* DNA reads in some of the samples is congruent with the usage of the cave as a stable during recent times. This contamination is primarily present in the samples closest to the pre-excavation surface of the W-X10 sondage (at the base of a large looter pit)(Figure 1), probably from ovid faecal sources. This contamination, however, does not affect the rest of the results, as the deamination values of the other taxa are noticeably different (Supplementary Data 3). We also detected the presence of contaminant modern human DNA in more than half of the samples (Supplementary Data 6). Only human mtDNA samples with more than 30% of C>T changes relative to the reference at read ends have been further examined in downstream analyses and identified as specific ancient human identifications.

We studied the edit distances of the individual alignments observing that most of the sample edit distances averages are between 1 and 2, which is consistent with metagenomic ancient DNA [52]. The edit distance is plotted together with coverage and damage using aDNA-BAMPlotter [53] for all the discussed individual mitochondrial sequences.

2. Statistical analyses

Efficiency and DNA preservation

We used the total numbers of classified reads at the species level, focusing on the ones with compelling evidence of ancient origin (Supplementary Data 3), to elaborate on patterns of DNA preservation. The average of classified reads per sample is 12,904. All the Mousterian samples are below this number. We then corrected the total amount of present classified reads by the number of sequenced reads (Supplementary Data 1) and we calculated both the Average (0.001966016248) and the Median (0.001509933178). All the Mousterian samples (level 130) are below these numbers (Supplementary Data 5), the temporal trend shows less preservation in the older samples (Supplementary Figure 4).

We tested the regression (n=31) of the relative abundance of DNA reads against the midpoint of the modeled age for each sampled level to assess the relationship between time and DNA preservation (Supplementary Data 1). The relationship is statistically significant, with an R^2 value of 0.3849 and a *p-value* of 0.000152.

Additionally, we evaluated the regression (n=31) of inhibition test efficiency against the midpoint of the modeled age for each sampled level to further explore the relationship between time and DNA preservation. This relationship is also statistically significant, with an R^2 value of 0.4475 and a *p-value* of 0.000028. Both relationships are presented in Supplementary Figure 4.

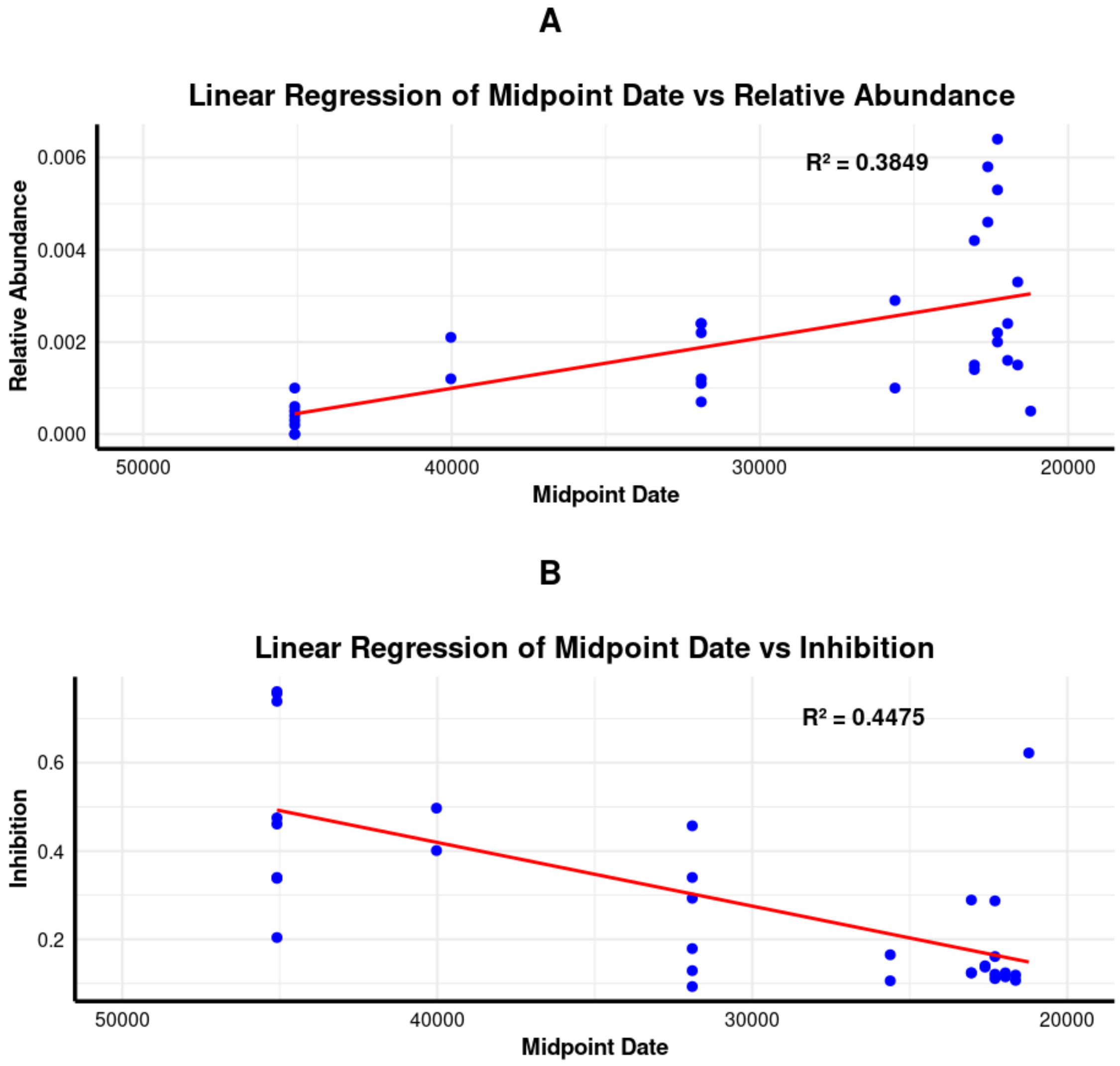

Supplementary Figure 4: DNA preservation and PCR efficiency vs. sample age. A) Relative abundance of classified reads vs. level age B) Efficiency of the PCR reaction vs. level age. The regression values show that the older samples have less relative DNA presence and less inhibition.

The previous results suggest that the DNA preservation is negatively related to sample age and the efficiency of the DNA amplification is positively related to sample age.

sedaDNA and Archaeofauna relationship

Prey animals are the most present in the faunal assemblages and are relevant to assessing subsistence patterns among human populations[54]. At the species level, it is clear that most of the genetic material belongs to Spanish ibex (*Capra pyrenaica)* and red deer (*Cervus*

elaphus). Previous archaeozoological taxa identifications reported that 47% of the Upper Palaeolithic ungulate faunal remains from El Mirón correspond to Spanish ibex, followed by red deer (19%) and chamois (6%)[10] (Supplementary Data 7). Our findings indicate that the sedaDNA of red deer represents an average 27% of the reads, followed by Spanish ibex with 23% and Chamois with 6% (Supplementary Data 3, Figure 2).

We next tested the possibility that the observations of sedaDNA and archeozoofauna show the same trend. We first converted Supplementary Data 7 into relative values according to the total sample per level (total (NISP)[10,13,55,56] or total classified reads at the species level). This enables us to compare the values across the sample profile. The obtained relative values are presented in Supplementary Table 3.

Supplementary Table 3: Relative presence of the taxa classified at the individual level. The series labelled as sedaDNA refer to sedaDNA observations, the others to NISP observations. The results are extracted from Supplementary Data 4.

Level	130	129	128	127	126	125	124	123	122	121	119
Canis-DNA	0.36	4.63	3.55	7.93	9.41	20.9	20.7	11.11	12.59	8.59	0.14
Canis	57.1	0.0	28.5	0.0	14.2	0.0	0.0	0.0	0.00	0.00	0.00
Vulpes-DNA	0.00	11.89	6.86	2.65	46.89	3.66	5.18	7.45	8.48	6.70	0.22
Vulpes	0.00	0.00	0.00	0.00	0.00	0.00	0.00	100.0	0.00	0.00	0.00
Cuon-DNA	6.78	15.73	27.9	22.1	3.38	4.07	4.57	2.87	7.50	1.88	3.14
Cuon	0.00	0.00	0.00	0.00	0.00	0.00	0.00	0.00	0.00	0.00	0.00
Cervus-DNA	2.76	4.08	9.05	8.01	8.14	19.3	11.1	10.94	12.66	13.31	0.54
Cervus	2.45	1.31	7.19	3.27	2.29	11.9	9.64	1.14	17.32	17.48	25.8
Capreolus-DNA	0.00	0.00	0.00	0.00	0.00	0.00	0.00	0.00	0.00	0.00	100
Capreolus	0.00	0.00	0.00	0.00	0.00	0.00	0.00	0.00	0.00	0.00	100
Rangifer-DNA	0.00	0.00	100.	0.00	0.00	0.00	0.00	0.00	0.00	0.00	0.00
Rangifer	0.00	0.00	0.00	0.00	0.00	0.00	0.00	0.00	0.00	0.00	0.00

Capra-DNA	6.36	6.47	12.6	6.56	14.38	10.2	10.6	10.34	9.94	11.28	1.13
Capra	7.86	3.78	12.5	3.78	1.16	14.1	8.59	1.75	15.57	11.21	19.6
Rupicapra-DNA	1.18	12.28	8.08	13.7	4.08	6.58	18.4	2.16	19.23	14.27	0.00
Rupicapra	9.90	0.99	9.90	0.00	8.91	4.95	9.90	1.98	18.81	21.78	12.8
Bos-DNA	19.7	15.34	21.4	2.67	8.90	1.49	14.6	4.98	2.59	5.98	2.25
Bos	0.00	0.00	0.00	0.00	0.00	0.00	0.00	0.00	11.76	0.00	88.2
Bison-DNA	7.00	65.01	0.00	23.0	4.97	0.00	0.00	0.00	0.00	0.00	0.00
Bison	0.00	0.00	0.00	0.00	0.00	0.00	0.00	0.00	0.00	0.00	0.00
Equus-DNA	8.00	24.76	10.5	1.44	13.85	23.2	8.43	2.38	4.70	2.71	0.00
Equus	11.7	0.00	0.00	0.00	0.00	0.00	5.88	0.00	0.00	0.00	82.3
Panthera-DNA	18.9	5.84	24.8	3.39	7.67	29.5	1.87	3.38	0.00	1.79	2.63
Panthera	0.00	0.00	100.	0.00	0.00	0.00	0.00	0.00	0.00	0.00	0.00
Lynx-DNA	0.00	0.00	13.8	0.00	86.13	0.00	0.00	0.00	0.00	0.00	0.00
Lynx	0.00	0.00	0.00	0.00	0.00	0.00	0.00	0.00	0.00	0.00	0.00
Ursus-DNA	10.9	40.13	9.29	4.18	24.17	0.00	0.00	4.67	2.53	1.58	2.51
Ursus	60.0	0.00	0.00	0.00	0.00	0.00	0.00	0.00	0.00	0.00	40.0
Crocuta-DNA	59.2	24.15	4.32	0.99	1.17	1.60	0.00	0.00	0.00	3.58	4.97
Crocuta	0.00	0.00	0.00	0.00	0.00	0.00	0.00	0.00	0.00	0.00	0.00
Lepus-DNA	0.00	6.78	0.00	8.47	19.92	8.26	16.7	14.92	7.98	16.90	0.00
Lepus	6.25	16.67	66.6	3.13	0.00	1.04	3.13	0.00	0.00	1.04	2.08

We then tested the possibility that the two observations (NISP and sedaDNA) describe the same underlying phenomenon. The data consists of discrete values, and we assume that these two observations are paired and that the distribution does not follow normality. The Kolmogorov-Smirnov (KS) test is a non-parametric statistical test used to compare two datasets' cumulative distribution functions (CDFs). It's particularly useful for determining whether two samples are likely to come from the same distribution. The KS test compares the shape and spread of two distributions, here, we use it to compare if

sedaDNA and NISP observations come from the same distribution. We used Rstudio [37] to test the relationship. The results are presented in Supplementary Table 4.

Supplementary Table 4: Kolmogorov-Smir*nov two-sided test results* regarding the possible relationship between NISP and sedaDNA at El Mirón.

Taxa	D value	*p-value*
Equus	0.63636	0.02325
Rangifer	Not Tested	Not Tested
Cervus	0.27273	0.8326
Capreolus	Not Tested	Not Tested
Bos	0.81818	0.001268
Bison	Not Tested	Not Tested
Capra	0.27273	0.8079
Rupicapra	0.18182	0.9934
Lepus	0.54545	0.0758
Crocuta	Not Tested	Not Tested
Canis	0.72727	0.005946
Vulpes	0.81818	0.001268
Cuon	Not Tested	Not Tested
Panthera	Not Tested	Not Tested
Lynx	Not Tested	Not Tested
Ursus	0.63636	0.02325

Results interpretation: Four taxa present no significant *p-value* ($p<0.05$): *Lepus, Capra, Rupicapra* and *Cervus*. In summary, according to the KS test results, there is insufficient evidence to conclude that, in these four taxa, the DNA and Zooarcheological results come from different distributions. The high *p-value* suggests that any observed differences in the distributions of relative presence are likely due to chance. These results suggest that the main animals of prey, *Capra, Rupicapa,* and *Cervus,* do not have significant differences in relative presence in both datasets. Surprisingly, this is not observed for *Canis,* which presents significant sedaDNA across the profile. Overall, The results suggest that sedaDNA matches the zooarcheological distribution results when the taxa have large numbers of representation in the archeofaunal and sedaDNA observations.

We next tested the possibility of correlation between both datasets. Spearman's correlation coefficient assesses the strength and direction of association between two ranked variables. It is suitable when the data does not meet the assumptions of normality or when there is not an assumption of linearity. We used Rstudio 2023.12.1 and R version 4.1.2 [37] to test the relationship. The results are presented in Supplementary Table 5.

Supplementary Table 5: Spearman's correlation test results regarding the possible relationship between NISP and sedaDNA at El Mirón.

Taxa	Spearman's correlation coefficient (rho)	*p-value*	*Sample (n)*
Equus	-0.3237481	0.3314398	11
Rangifer	Not Tested	Not Tested	-
Cervus	0.2909091	0.3864346	11
Capreolus	Not Tested	Not Tested	-
Bos	-0.5258759	0.09660888	11
Bison	Not Tested	Not Tested	-
Capra	-0.277905	0.407993	11
Rupicapra	0.1743193	0.608212	11
Lepus	-0.5953553	0.05330582	11
Crocuta	Not Tested	Not Tested	
Canis	-0.4393724	0.1763393	11
Vulpes	0.2	0.5554454	11
Cuon	Not Tested	Not Tested	-
Panthera	Not Tested	Not Tested	-
Lynx	Not Tested	Not Tested	-
Ursus	0.108118	0.7516816	11

Results interpretation: The results show the presence of no significant correlation between both datasets at a statistical threshold of *p-value<0.05*.

Supplementary Note 4: Individual genomes and phylogenies analyses

In this Note, we describe the analyses of the individual mtDNA sequences from the sediments and their comparisons with present-day and ancient diversity. Consensus sequences were merged with present-day and ancient sequences and were aligned using MAFFT 7.52 [57]. Maximum Likelihood trees were produced using partial deletions of 95%, GTR substitution model and 100 bootstrap replications using MEGA 6 [58]. We also estimated the presence of missing sites on the consensus sequences, counting the number of "N" with a tailor-made script. Trees were produced with Fig Tree 1.4.4 [59] and TreeViewer[60]. For classifying the *C. crocuta* sequences from sample ElMiron_10 we performed a pairwise distance matrix in MEGA[58] 10.2.4 using 100 bootstrap replications, 95% partial deletion and the Maximum composite method. The datasets used for each of the analyses are presented in Supplementary Data 12. We have used more than 750 mtDNA sequences from present-day and ancient genomes from the following publications: [8,9,24,61–156]. The read length distributions of the taxa from the recovered partial genomes (Supplementary Data 5) are described individually in this Note.

1. Cuon alpinus

We analyzed three mitochondrial DNA (mtDNA) sequences from *Cuon alpinus*: ElMiron_8, ElMiron_9, and ElMiron_7 (Supplementary Figures 5-7). For reconstructing the consensus sequences and subsequent alignments, we used the reference sequence NC_013445.1 (*Cuon alpinus*). These sequences exhibit 31 variable sites across their length. Coverage plots indicate that the sequences are incomplete; indeed, *Cuon alpinus* shows one of the highest rates of missing sites among all animals examined in this study. The average across the 70 recovered genomes is 3,877 missing sites. Notably, all three *Cuon* sequences have nearly double this number of missing sites (Supplementary Figure 6, Supplementary Data 3).

The mapping plot analysis (Supplementary Figure 6) shows that the recovered sequences present coverage gaps compared to the reference (NC_013445.1), representing an Asian individual. The reads aligning to *Cuon* sequences exhibit a lower edit distance (average = 1.3, SD = 0.19) compared to those aligning to *Canis* sequences (average = 1.6, SD = 0.27). This difference suggests that we are only recovering more conserved regions of the *Cuon*

sequence. This could be explained for the fact that the sequence of *Cuon alpinus* was not present in the capture design and therefore we only recovered more conserved regions.

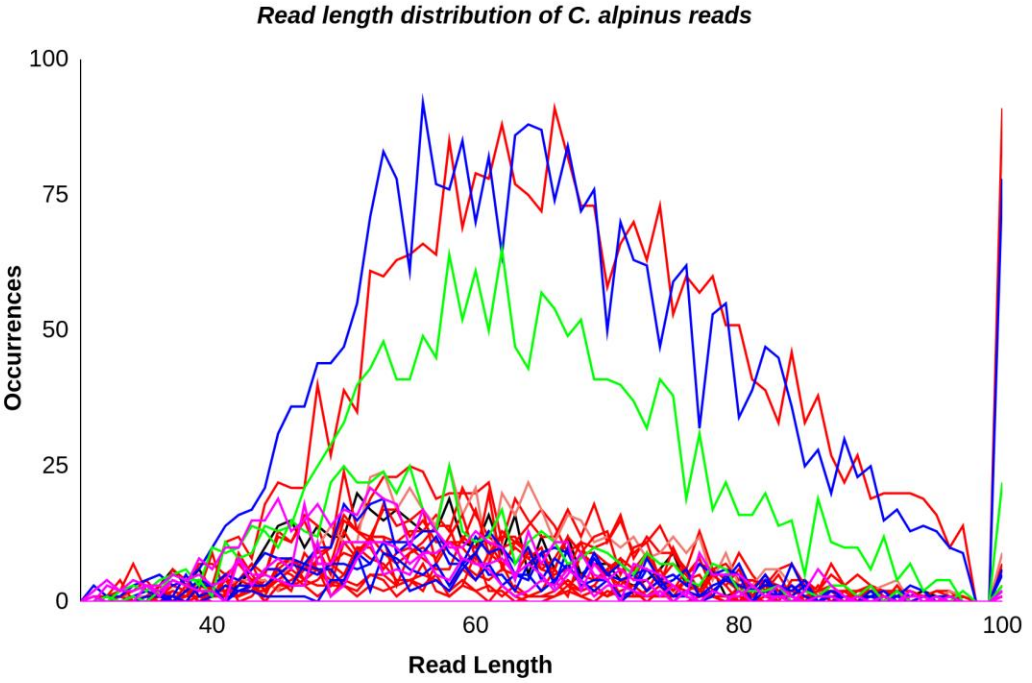

Supplementary Figure 5: read length distribution of El Mirón *C. alpinus* sequences. Black depicts Initial Magdalenian 119.2 level, Red depicts Solutrean 121-127 levels, Blue depicts Gravettian 128 level, Green depicts semi-sterile 129 level, Purple depicts Mousterian 130 level.

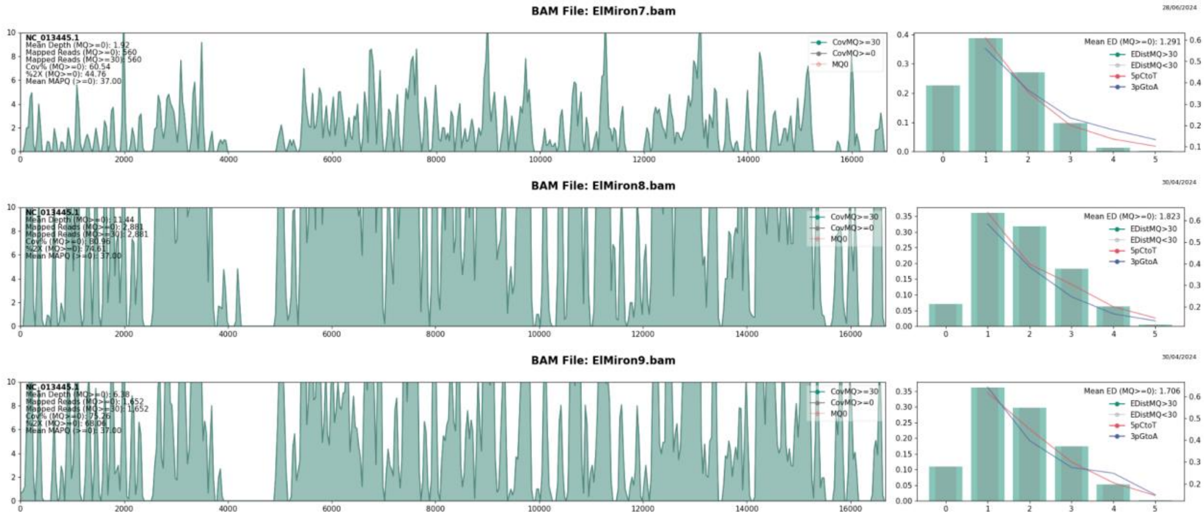

Supplementary Figure 6*:* Coverage plots and damage plots of *C. alpinus* sedaDNA mtDNA genomes.

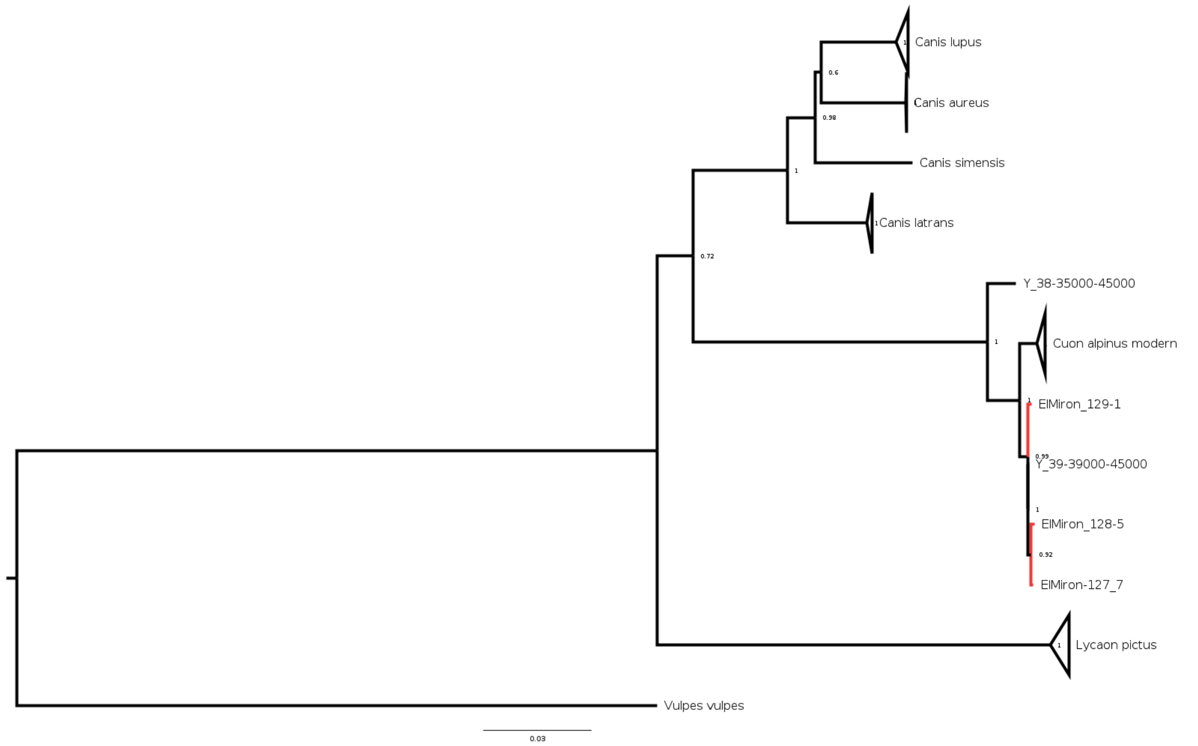

Supplementary Figure 7: Maximum likelihood tree of the *Cuon mtDNA* genomes. Red denotes the *Cuon alpinus* mtDNA genomes from El Mirón soil samples*.* Node numbers denote bootstrap values. The tree is rooted with *Vulpes vulpes* mtDNA. All the sequences from El Mirón are located close to sample Y-39 from Bacho Kiro in Bulgaria dated to the late Pleistocene.

2. Canis lupus

For the *C. lupus* analyses*,* We used samples: ElMiron_1, ElMiron_2, ElMiron_14, ElMiron_15, ElMiron_3, ElMiron_16, ElMiron_4, ElMiron_17, ElMiron_5, ElMiron_6, ElMiron_7, ElMiron_19, ElMiron_20, ElMiron_21 (Supplementary Figures 8-11). For reconstructing the consensus sequences as well as the individual alignments we used the reference sequence NC_008092.1. Most of the sequences are almost complete, with several with only a few hundred missing sites, being one of the species with the highest values of covered sequences (Supplementary Figure 10, Supplementary Data 3). This shows the similarity between the reference sequence and the El Mirón Pleistocene sequences. As *C. lupus* is one of the taxa with more recovered genomes from El Mirón sediments, we have tested the presence of a correlation between the coverage and the missing sites. We observe that with higher coverage, the amount of missing sites is

decreased in a relationship that is not linear (Supplementary Figure 9). We tested the significance of the correlation with Sperman's correlation test and the relationship is highly significant n=14, rho=-0.996 and *p-value*=1.3e-13.

We constructed the multiple sequence alignment with our 14 samples plus 215 other sequences of *C. lupus and* four outliers (NC_013445.1 plus El_Mirón8, ElMiron_7 and ElMiron_8 *C. alpinus* sequence)

The alignment, excluding outgroups, has 2039 variable positions and 194 variable positions within El Mirón 14 sequences.

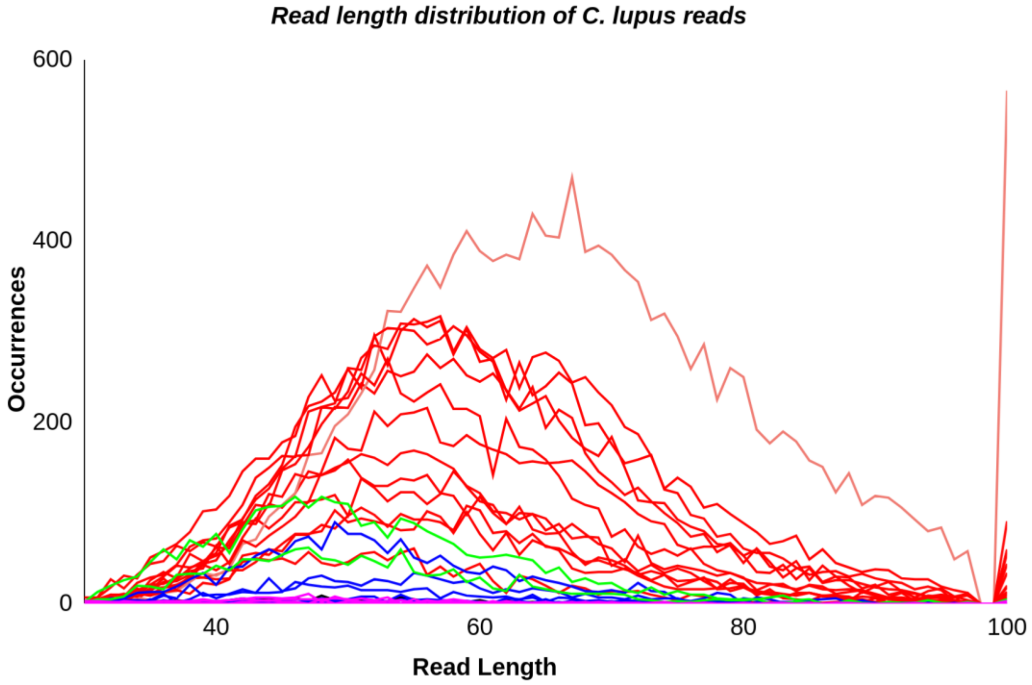

Supplementary Figure 8: read length distribution of El Mirón *C. lupus* sequences. Black depicts Initial Magdalenian 119.2 level, Red depicts Solutrean 121-127 levels, Blue depicts Gravettian 128 level, Green depicts semi-sterile 129 level, Purple depicts Mousterian 130 level.

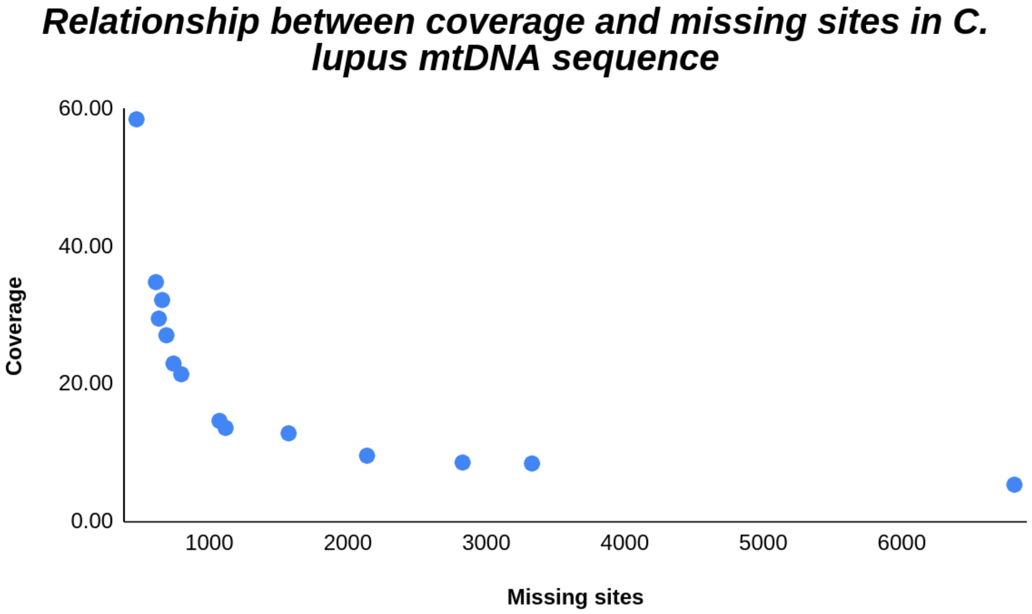

Supplementary Figure 9: Relationship between missing sites and coverage in the reads attributed to *C. lupus* from El Mirón. The distribution suggests that significant yields are necessary to increase the coverage across the sequence with the selected methodology.

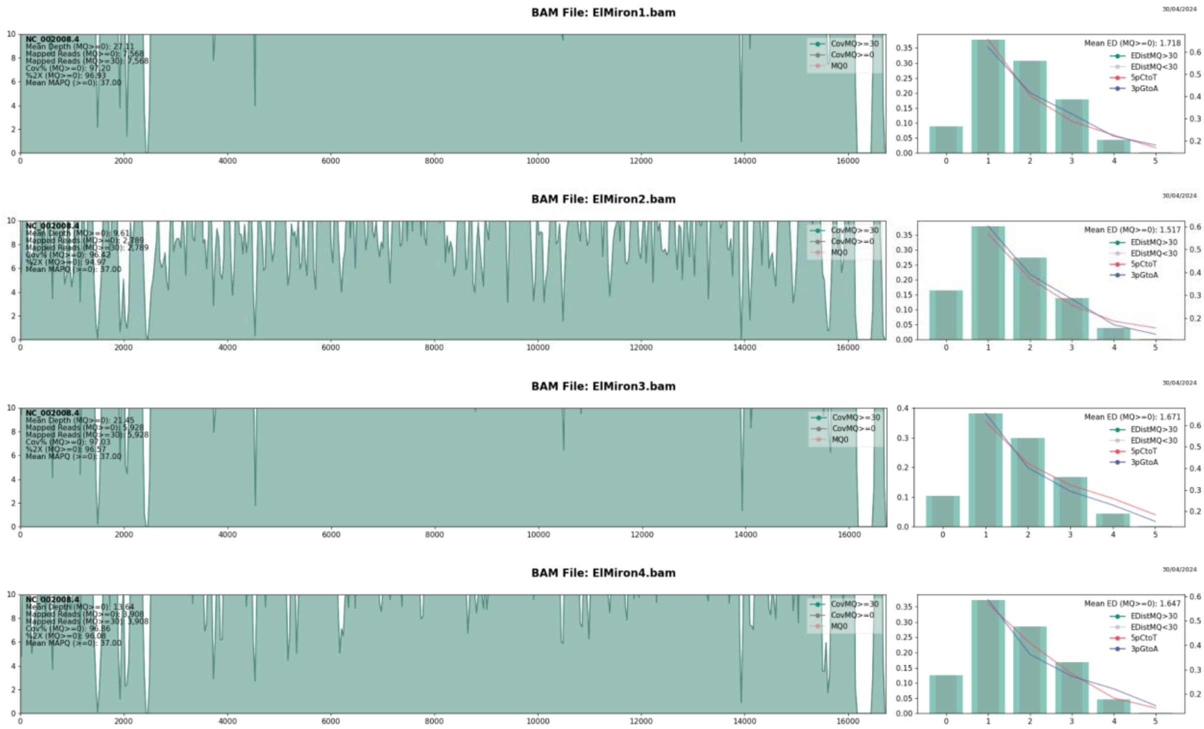

BAM File: ElMiron5.bam

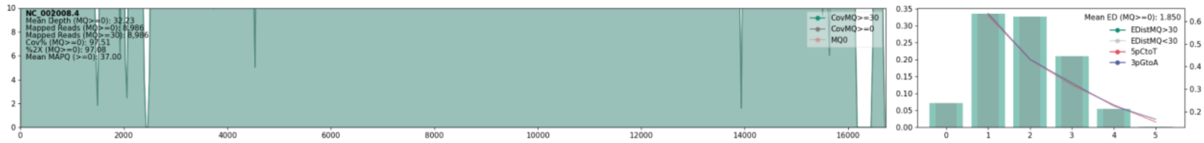

BAM File: ElMiron6.bam

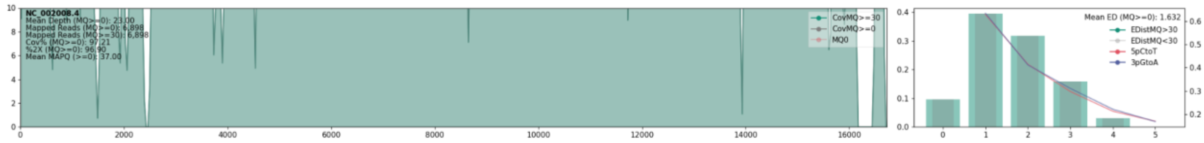

BAM File: ElMiron7.bam

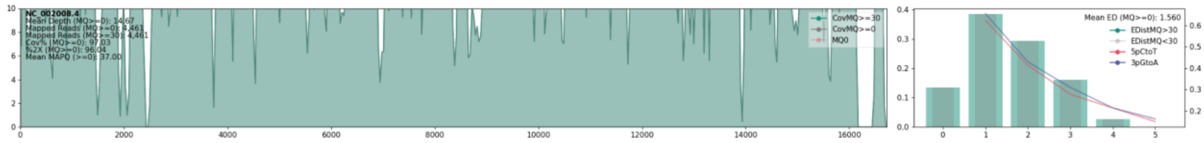

BAM File: ElMiron14.bam

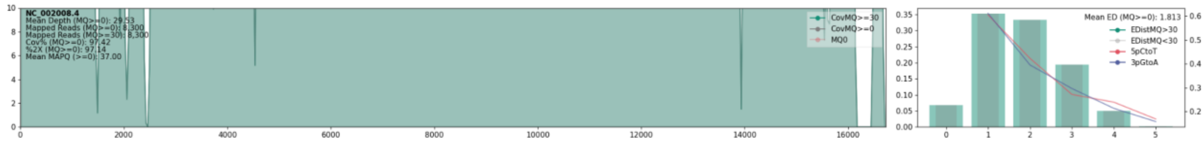

BAM File: ElMiron15.bam

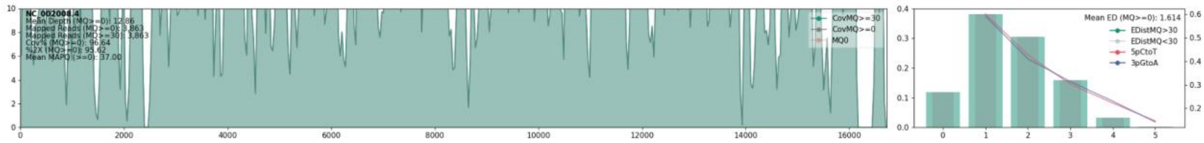

BAM File: ElMiron16.bam

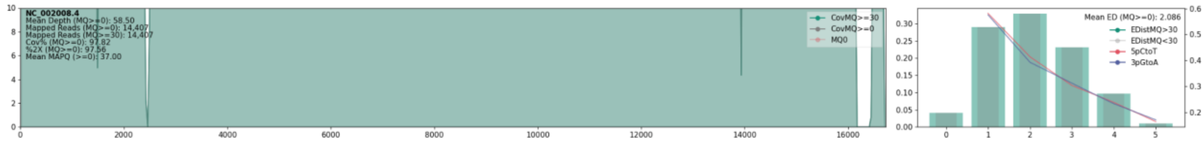

BAM File: ElMiron17.bam

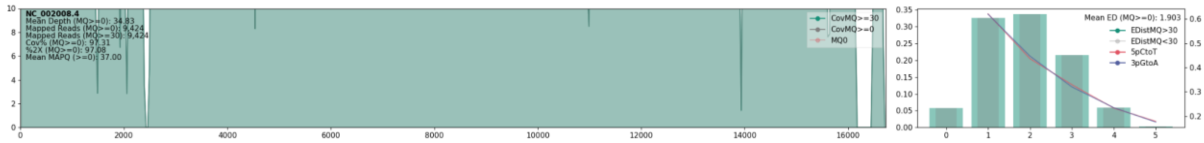

BAM File: ElMiron19.bam

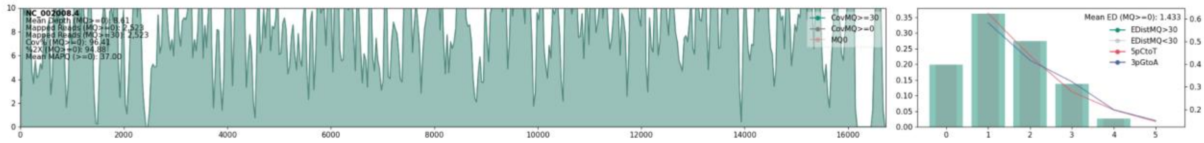

BAM File: ElMiron20.bam

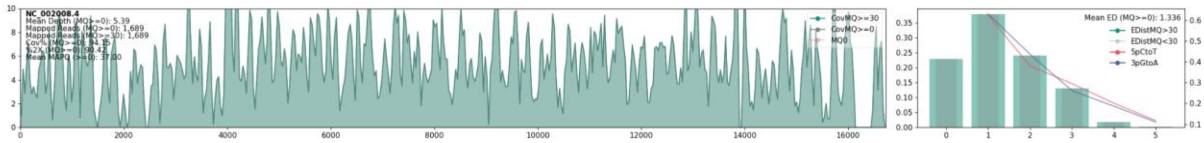

BAM File: ElMiron21.bam

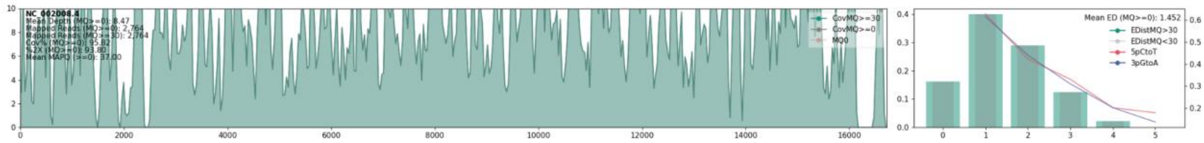

Supplementary Figure 10: Coverage and damage plots of *C. lupus* sedaDNA sedaDNA mtDNA genomes.

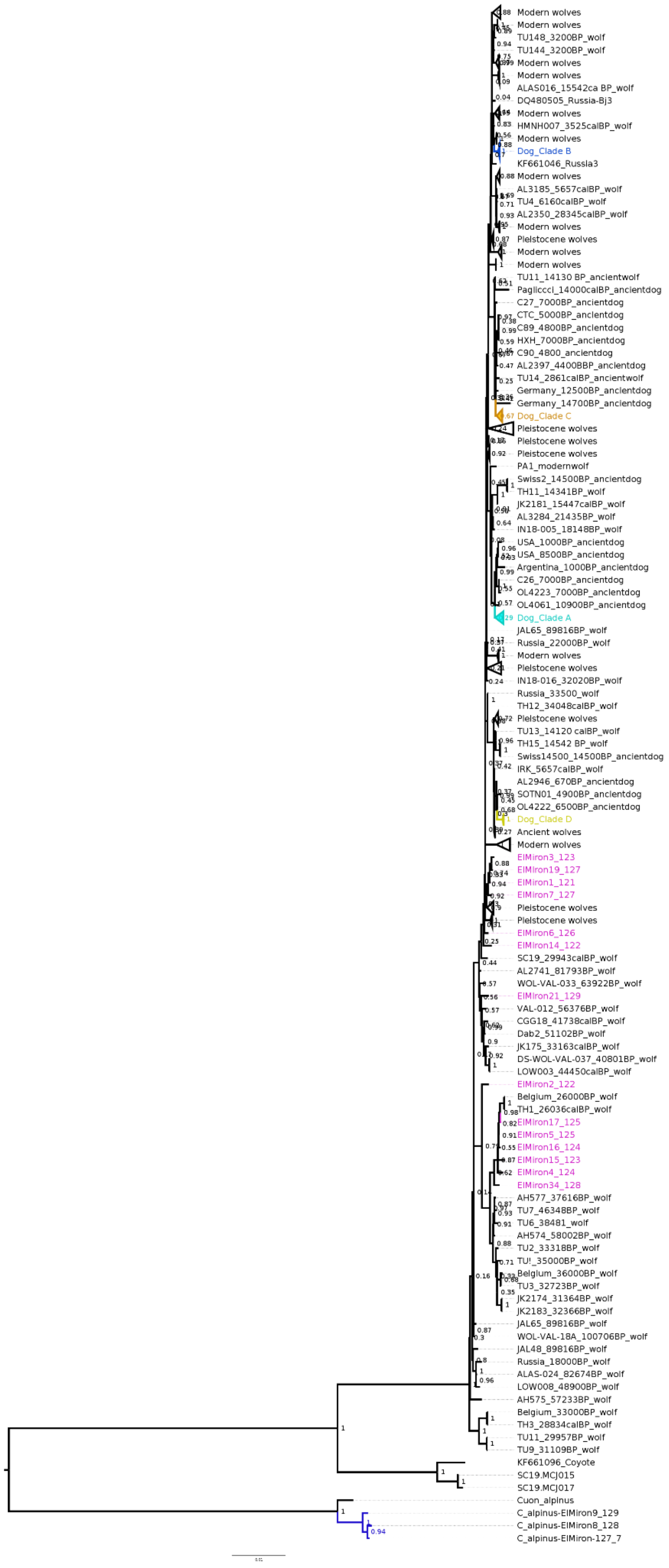
Modern wolves
Modern wolves
TU148_3200BP_wolf
TU144_3200BP_wolf
Modern wolves
Modern wolves
ALAS016_15542ca BP_wolf
DQ480505_Russia-Bj3
Modern wolves
HMNH007_3525calBP_wolf
Modern wolves
Dog_Clade B
KF661046_Russia3
Modern wolves
AL3185_5657calBP_wolf
TU4_6160calBP_wolf
AL2350_28345calBP_wolf
Modern wolves
Pleistocene wolves
Modern wolves
Modern wolves
TU11_14130 BP_ancientwolf
Paglicci_14000calBP_ancientdog
C27_7000BP_ancientdog
CTC_5000BP_ancientdog
C89_4800BP_ancientdog
HXH_7000BP_ancientdog
C90_4800_ancientdog
AL2397_4400BBP_ancientdog
TU14_2861calBP_ancientwolf
Germany_12500BP_ancientdog
Germany_14700BP_ancientdog
Dog_Clade C
Pleistocene wolves
Pleistocene wolves
Pleistocene wolves
PA1_modernwolf
Swiss2_14500BP_ancientdog
TH11_14341BP_wolf
JK2181_15447calBP_wolf
AL3284_21435BP_wolf
IN18-005_18148BP_wolf
USA_1000BP_ancientdog
USA_8500BP_ancientdog
Argentina_1000BP_ancientdog
C26_7000BP_ancientdog
OL4223_7000BP_ancientdog
OL4061_10900BP_ancientdog
Dog_Clade A
JAL65_89816BP_wolf
Russia_22000BP_wolf
Modern wolves
Pleistocene wolves
IN18-016_32020BP_wolf
Russia_33500_wolf
TH12_34048calBP_wolf
Pleistocene wolves
TU13_14120 calBP_wolf
TH15_14542 BP_wolf
Swiss14500_14500BP_ancientdog
IRK_5657calBP_wolf
AL2946_670BP_ancientdog
SOTN01_4900BP_ancientdog
OL4222_6500BP_ancientdog
Dog_Clade D
Ancient wolves
Modern wolves
ElMiron3_123
ElMiron19_127
ElMiron1_121
ElMiron7_127
Pleistocene wolves
Pleistocene wolves
ElMiron6_126
ElMiron14_122
SC19_29943calBP_wolf
AL2741_81793BP_wolf
WOL-VAL-033_63922BP_wolf
ElMiron21_129
VAL-012_56376BP_wolf
CGG18_41738calBP_wolf
Dab2_51102BP_wolf
JK175_33163calBP_wolf
DS-WOL-VAL-037_40801BP_wolf
LOW003_44450calBP_wolf
ElMiron2_122
Belgium_26000BP_wolf
TH1_26036calBP_wolf
ElMiron17_125
ElMiron5_125
ElMiron16_124
ElMiron15_123
ElMiron4_124
ElMiron34_128
AH577_37616BP_wolf
TU7_46348BP_wolf
TU6_38481_wolf
AH574_58002BP_wolf
TU2_33318BP_wolf
TU!_35000BP_wolf
Belgium_36000BP_wolf
TU3_32723BP_wolf
JK2174_31364BP_wolf
JK2183_32366BP_wolf
JAL65_89816BP_wolf
WOL-VAL-18A_100706BP_wolf
JAL48_89816BP_wolf
Russia_18000BP_wolf
ALAS-024_82674BP_wolf
LOW008_48900BP_wolf
AH575_57233BP_wolf
Belgium_33000BP_wolf
TH3_28834calBP_wolf
TU11_29957BP_wolf
TU9_31109BP_wolf
KF661096_Coyote
SC19.MCJ015
SC19.MCJ017
Cuon_alpinus
C_alpinus-ElMiron9_129
C_alpinus-ElMiron8_128
C_alpinus-ElMiron-127_7
0.01

Supplementary Figure 11: Maximum likelihood tree of the *Canis lupus mtDNA* genomes. Pink denotes the *Canis lupus* mtDNA genomes from El Mirón soil samples. Node numbers denote bootstrap values. The tree is rooted with dhole mtDNA sequences, including three from El Mirón. All the sequences from El Mirón are located within the Pleistocene *C. lupus* diversity of Eurasia.

3. Vulpes vulpes

We analysed four mtDNA sedaDNA genomes: ElMiron_1, ElMiron_6, ElMiron_14 and El_Mirón21 (Supplementary Figures 12-14). We used the reference sequence NC_008434.1 to reconstruct the consensus sequences. The Mirón sedaDNA mtDNA sequences were aligned with other 12 *Vulpes spp.* sequences and 2 outgroup. One sequence is almost complete, only presenting 1,653 missing sites (Supplementary Figure 13, Supplementary Data 3).

The alignment, excluding outgroups, has 3280 variable sites. Within the four El Mirón samples, there are 39 variable sites.

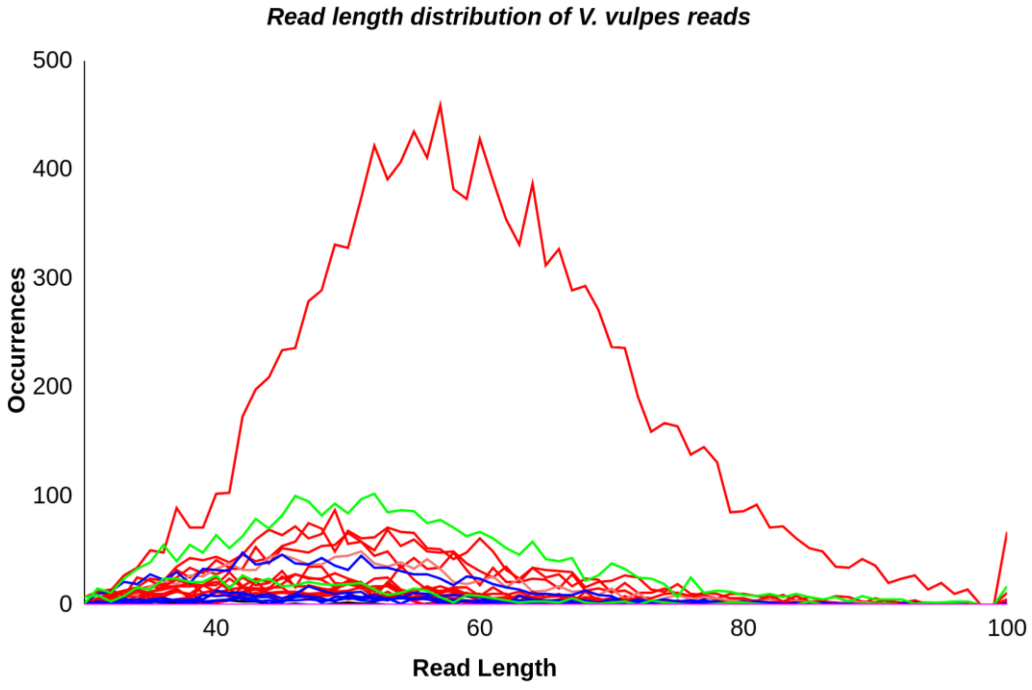

Supplementary Figure 12: Read length distribution of El Mirón *V. vulpes* sequences. Black depicts Initial Magdalenian 119.2 level, Red depicts Solutrean 121-127 levels, Blue

depicts Gravettian 128 level, Green depicts semi-sterile 129 level, Purple depicts Mousterian 130 level.

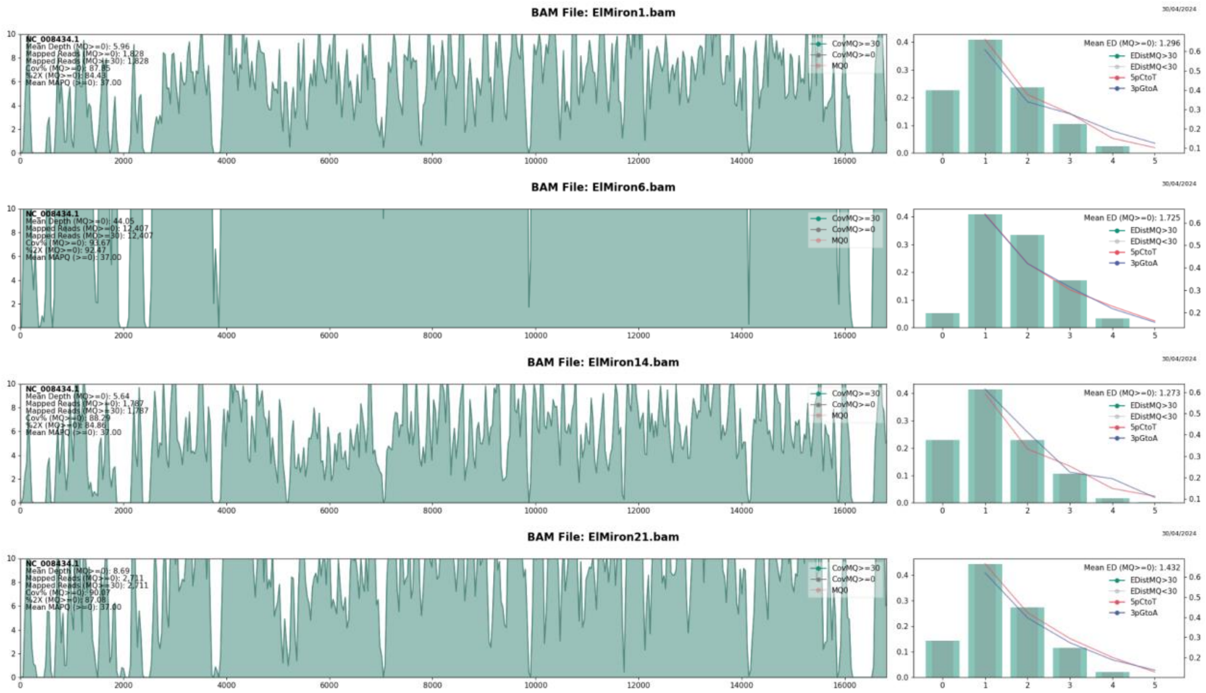

Supplementary Figure 13: Coverage plots and damage plots of *V. vulpes* sedaDNA mtDNA genomes.

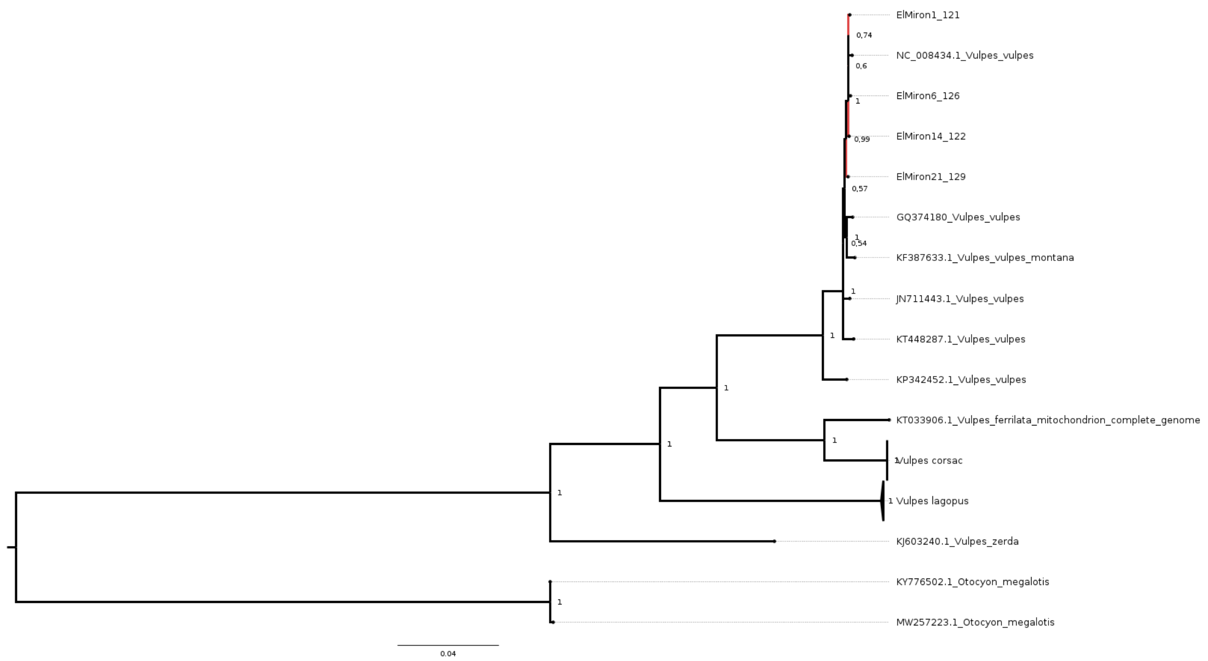

Supplementary Figure 14: Maximum likelihood tree of the *Vulpes vulpes* seda mtDNA genomes. The red colour denotes the sedaDNA sequences from El Mirón soil samples. Node numbers denote bootstrap values. The tree is rooted in *Otocyon megalotis*. The sequences from El Mirón are located within the diversity of *V. vulpes.*

4. Crocuta crocuta

We recovered a partial genome of *Crocuta crocuta* from sample El Miron_10 (Supplementary Figures 15-16). For reconstructing the consensus sequences as well as the individual alignments we used the reference sequence NC_020670.1. We compared the sequence with other 23 present day and ancient *Crocuta crocuta* diversity using a distance matrix.

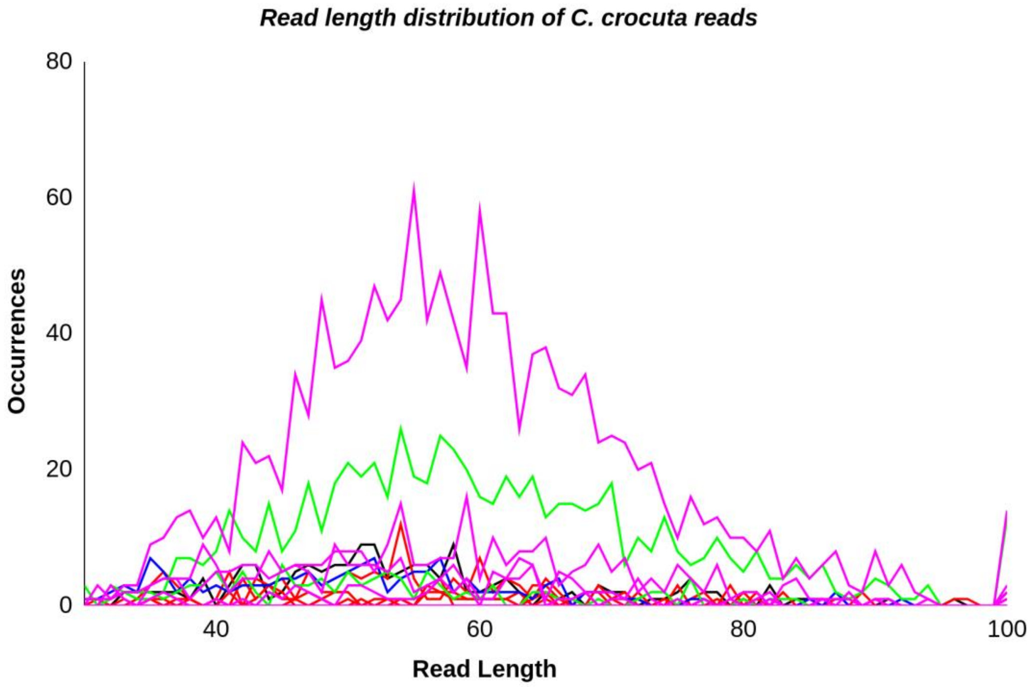

Supplementary Figure 15: Read length distribution of El Mirón *C. crocuta.* Black depicts Initial Magdalenian 119.2 level, Red depicts Solutrean 121-127 levels, Blue depicts Gravettian 128 level, Green depicts semi-sterile 129 level, Purple depicts Mousterian 130 level.

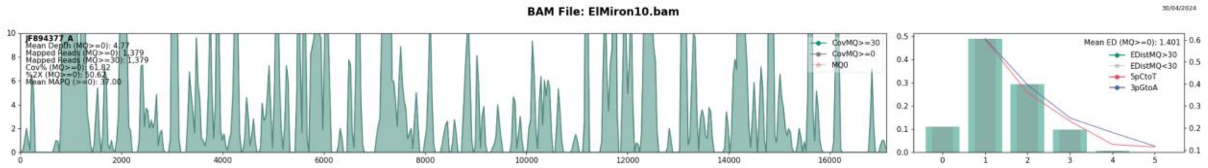

Supplementary Figure 16: Coverage and damage plot of *C. crocuta* mtDNA sedaDNA sequence.

5. *Panthera pardus*

We analysed two mtDNA sequences ElMiron_5 and ElMiron_9 (Supplementary Figures 17-19). For reconstructing the consensus sequences as well as the individual alignments we used the reference sequence NC_010641.1. We aligned our 2 new reported sequences together with 25 present-day and ancient *P. pardus* mtDNA genomes *and* 5 other *Panthera* genus sequences. The two sequences from El Mirón show 21 variable sites across the sequence.

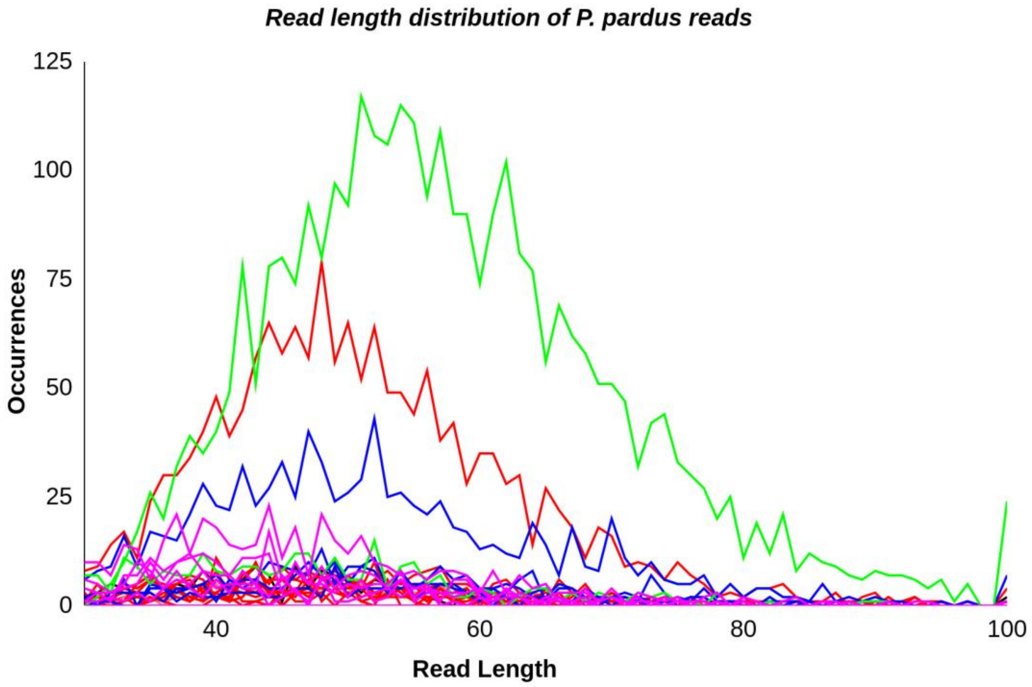

Supplementary Figure 17: Read length distribution of El Mirón *P. pardus* mtDNA sedaDNA sequences. Black depicts Initial Magdalenian 119.2 level, Red depicts Solutrean 121-127 levels, Blue depicts Gravettian 128 level, Green depicts semi-sterile 129 level, Purple depicts Mousterian 130 level.

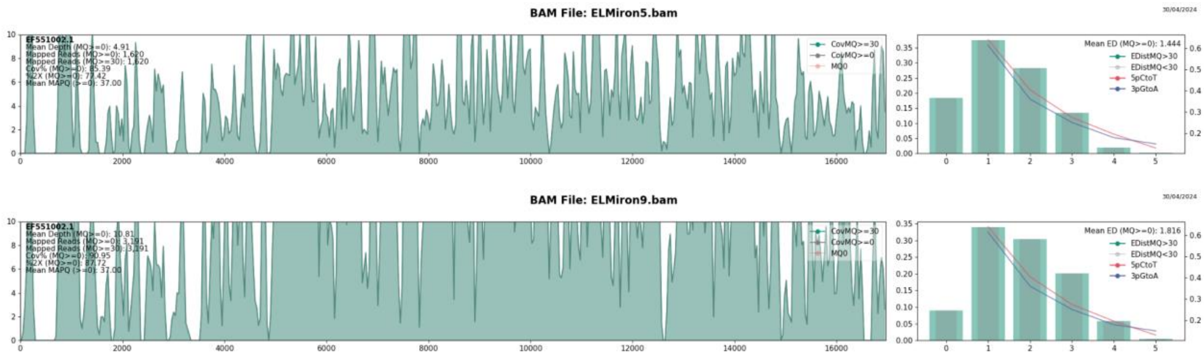

Supplementary Figure 18: Coverage and damage plots of *P. pardus* sedaDNA mtDNA sequences.

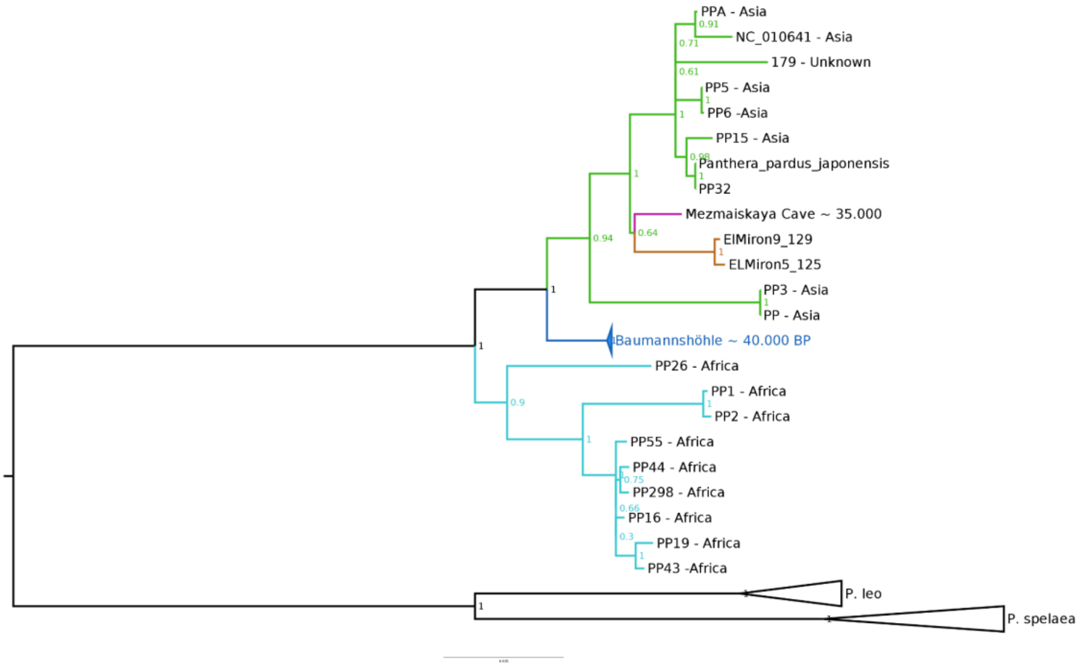

Supplementary Figure 19: Maximum likelihood tree of *Panthera* mtDNA genomes: A genomic similarity between El Mirón leopards and Caucasus *P. pardus* BAR001 from the Pleistocene is observed. Light blue corresponds to African leopards, Blue to Pleistocene European leopards, Purple for caucasian Pleistocene leopards and green for Asian leopards. El Mirón leopards are in brown.

6. Ursus arctos

We analysed one mtDNA partial genome, ElMiron_9 (Supplementary Figure 20-22). For reconstructing the consensus sequences and individual alignments, we used the reference sequence NC_003427.1. The sequence is almost fully covered, showing the similarity of *Ursus arctos* from El Mirón and the reference sequence (Supplementary Figure 21).

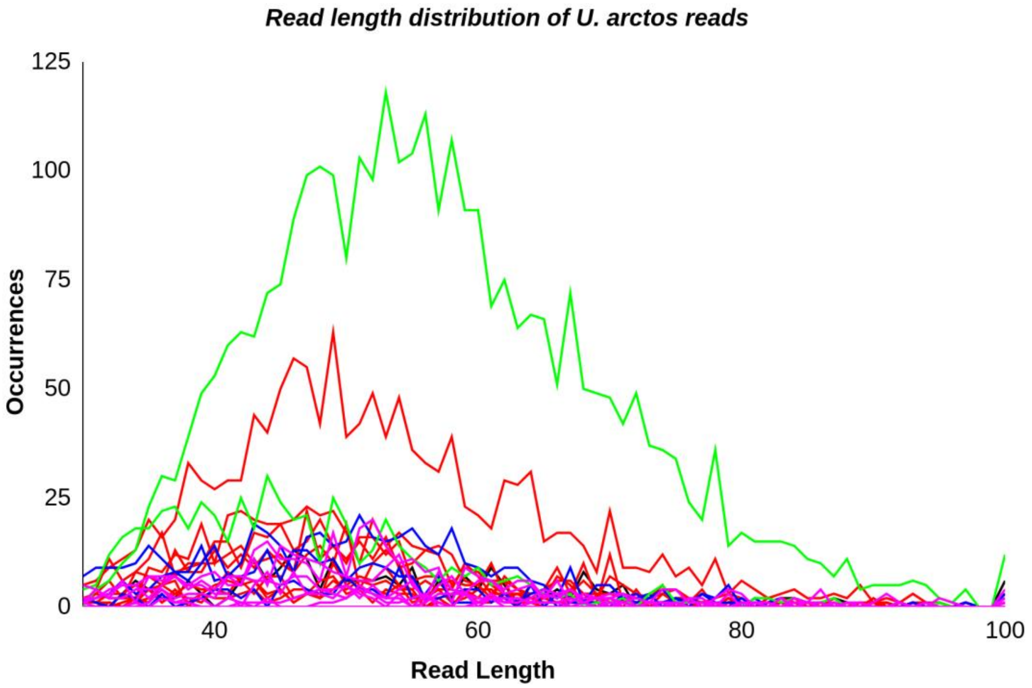

Supplementary Figure 20: Read length distribution of El Mirón *U. arctos* sequences. Black depicts the Initial Magdalenian 119.2 level, Red depicts the Solutrean 121-127 levels, Blue depicts the Gravettian 128 level, Green depicts the semi-sterile 129 level, and Purple depicts the Mousterian 130 level.

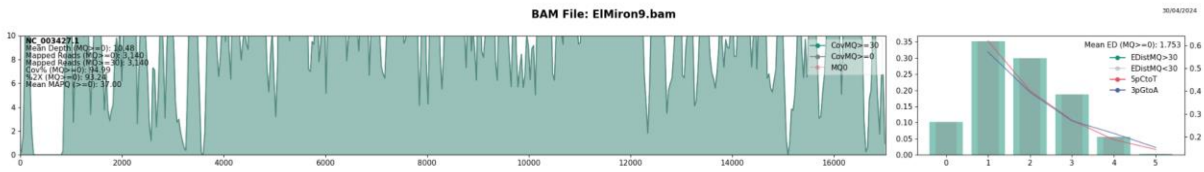

Supplementary Figure 21: *U. arctos sedaDNA mtDNA genomes coverage plots and damage plots.*

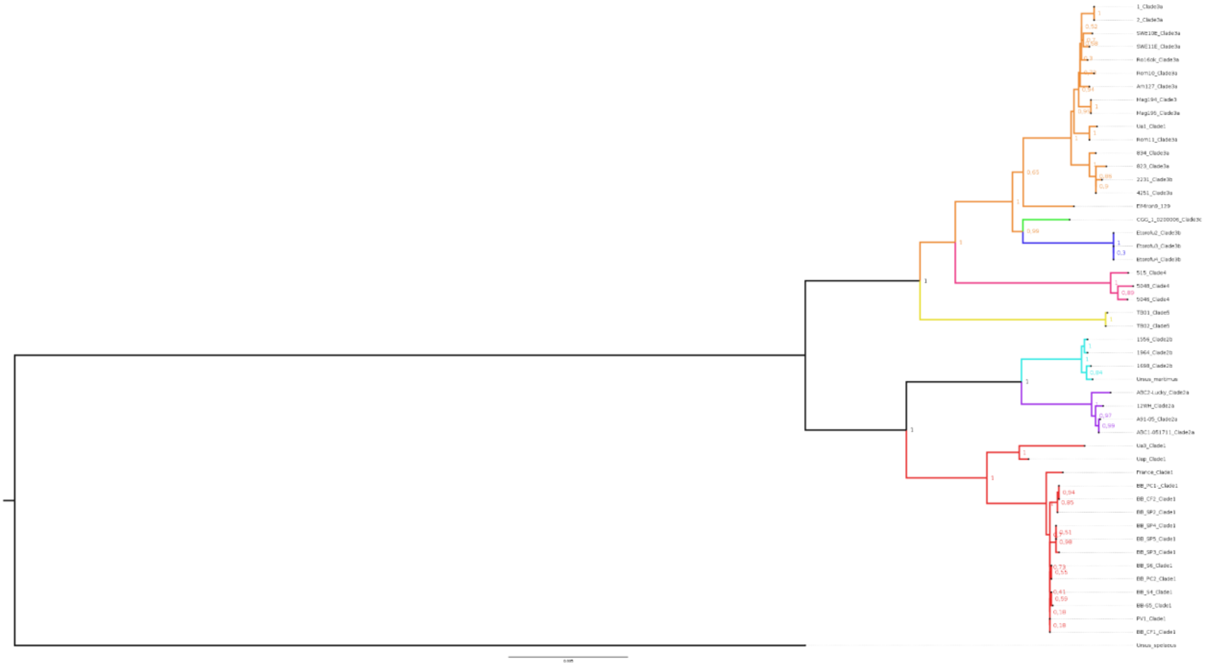

Supplementary Figure 22: Maximum likelihood tree of the *Ursidae* mtDNA genomes: The tree is rooted in *the U. spelaeus mtDNA genome. Colours denote clades. Orange denotes clade 3a, Dark blue clade 3b, Green clade 3C, Pink clade 4, Light blue clade 2b, Purple clade 2a, and red clade 1. ElMiron_9 from level 129 is placed in clade 3a, separated from other Pleistocene bears from Iberia,* which are placed in clade 1.

7. Cervus elaphus

We analysed 21 mtDNA partial genomes of *C. elaphus,* including: ElMiron_1, ElMiron_2, ElMiron_3, ElMiron_4, ElMiron_5, ElMiron_6, ElMiron_7, ElMiron_8, ElMiron_9, ElMiron_13, ElMiron_14, ElMiron_15, ElMiron_16, ElMiron_17, ElMiron_18, ElMiron_18 extract 2, ElMiron_19, ElMiron_20, ElMiron_28, ElMiron_30, ElMiron_34 (Supplementary Figures 23-26). For reconstructing the consensus sequences as well as the individual alignments we used the reference sequence NC_007704.2. Many sequences show the presence of limited missing sites, but others show elevated numbers (Supplementary Data 3). As *C. elaphus* is one of the taxa with more genomes, we have tested the presence of a relationship between the coverage and the missing sites. We observe that with higher coverage, the amount of missing sites is reduced (Supplementary Figure 24), in a relationship that is nonlinear. We tested the significance of the correlation with Sperman's tests, and the relationship is highly significant n=21, rho=-0.972 and *p-value*=1.8e^{-10}. The 21 sequences show 139 variable sites when aligned.

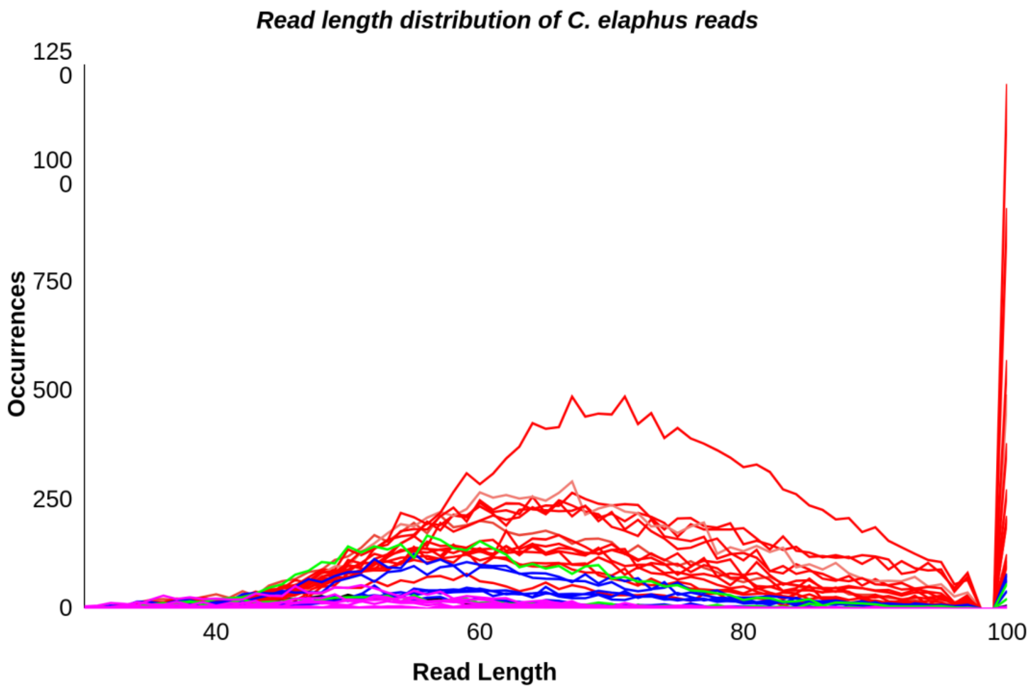

Supplementary Figure 23: Read length distribution of El Mirón *C. elaphus* sequences. Black depicts Initial Magdalenian 119.2 level, Red depicts Solutrean 121-127 levels, Blue depicts Gravettian 128 level, Green depicts semi-sterile 129 level, Purple depicts Mousterian 130 level.

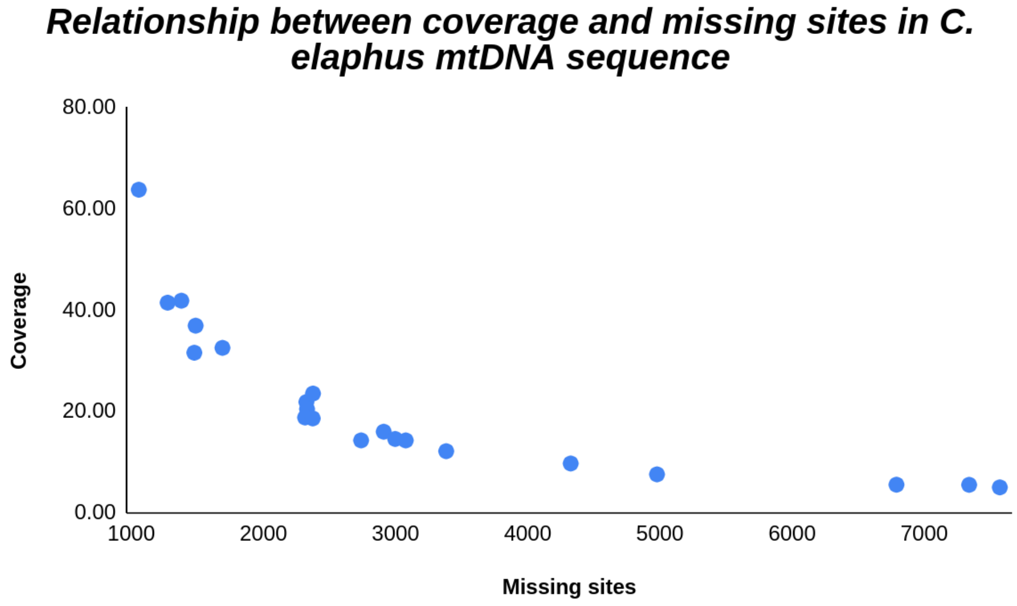

Supplementary Figure 24: Relationship between missing sites and coverage in the reads attributed to *C. elaphus* from El Mirón.

BAM File: ElMiron1.bam

01/05/2024

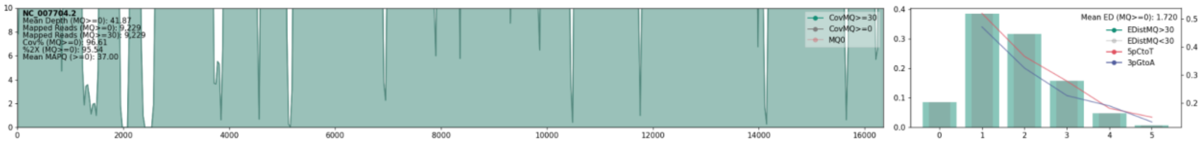

BAM File: ElMiron2.bam

01/05/2024

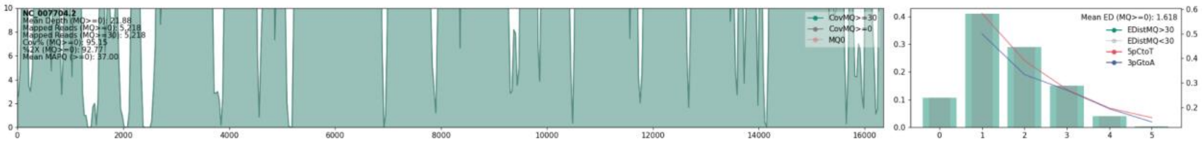

BAM File: ElMiron3.bam

01/05/2024

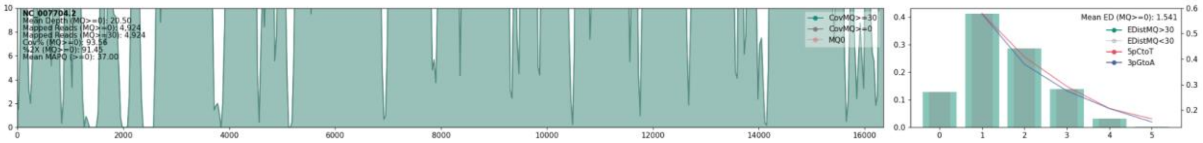

BAM File: ElMiron4.bam

01/05/2024

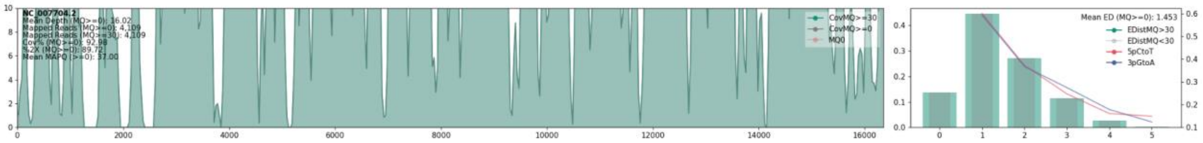

BAM File: ElMiron5.bam

01/05/2024

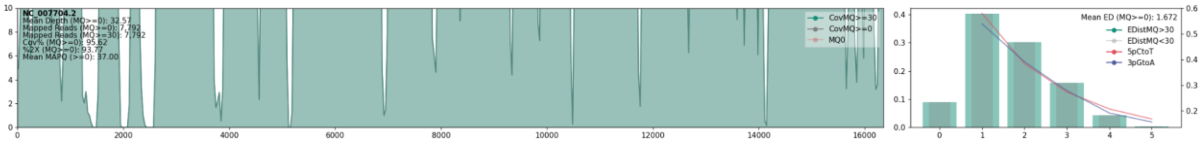

BAM File: ElMiron6.bam

01/05/2024

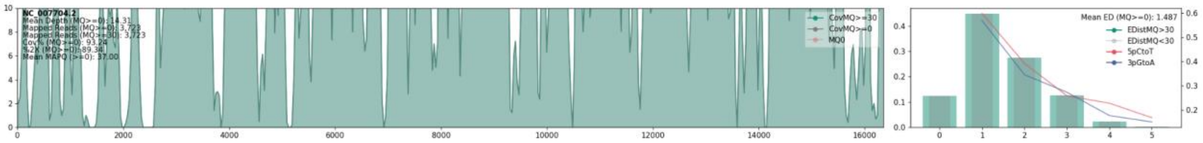

BAM File: ElMiron7.bam

01/05/2024

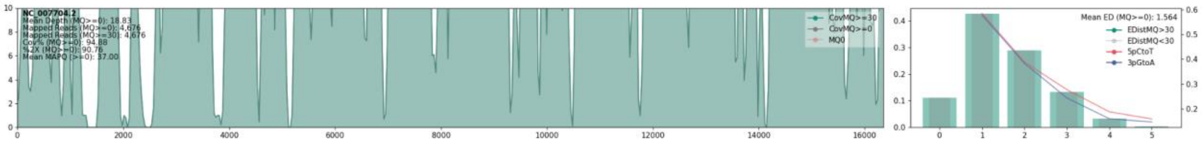

BAM File: ElMiron8.bam

01/05/2024

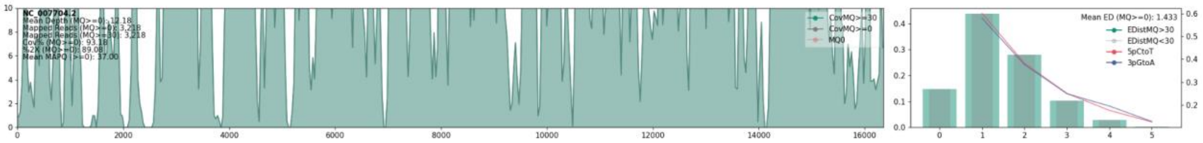

BAM File: ElMiron9.bam

01/05/2024

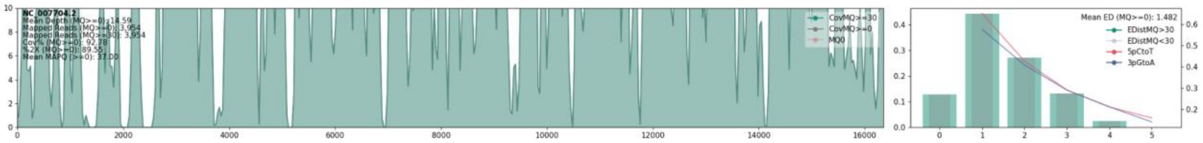

BAM File: ElMiron13.bam

01/05/2024

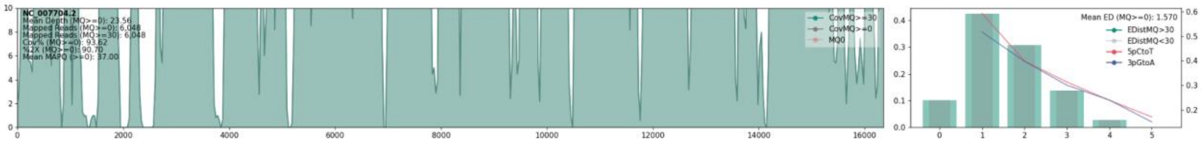

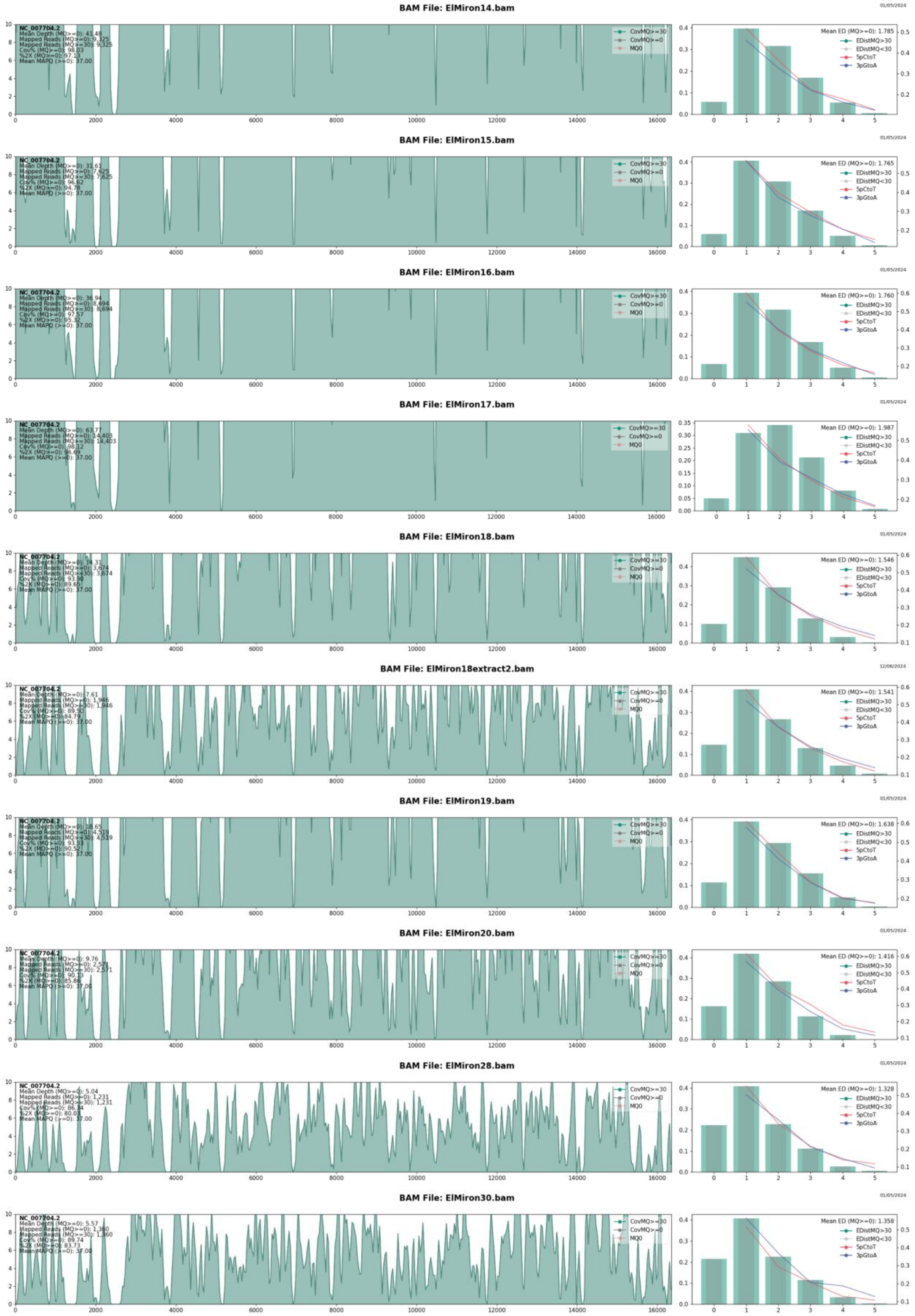
BAM File: ElMiron14.bam
01/05/2024
NC_007704.2
Mean ED (MQ>=0): 1.785
BAM File: ElMiron15.bam
01/05/2024
Mean ED (MQ>=0): 1.765
BAM File: ElMiron16.bam
01/05/2024
Mean ED (MQ>=0): 1.760
BAM File: ElMiron17.bam
01/05/2024
Mean ED (MQ>=0): 1.987
BAM File: ElMiron18.bam
01/05/2024
Mean ED (MQ>=0): 1.546
BAM File: ElMiron18extract2.bam
12/06/2024
Mean ED (MQ>=0): 1.541
BAM File: ElMiron19.bam
01/05/2024
Mean ED (MQ>=0): 1.638
BAM File: ElMiron20.bam
01/05/2024
Mean ED (MQ>=0): 1.416
BAM File: ElMiron28.bam
01/05/2024
Mean ED (MQ>=0): 1.328
BAM File: ElMiron30.bam
01/05/2024
Mean ED (MQ>=0): 1.358
CovMQ>=30
CovMQ>=0
MQ0
EDistMQ>30
EDistMQ<30
5pCtoT
3pGtoA

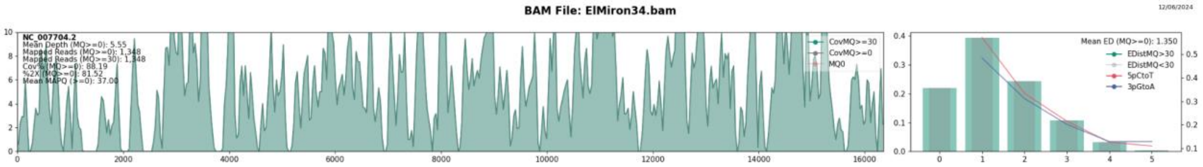

Supplementary Figure 25: Coverage plots and damage plots of *C. elaphus sedaDNA* mtDNA genomes.

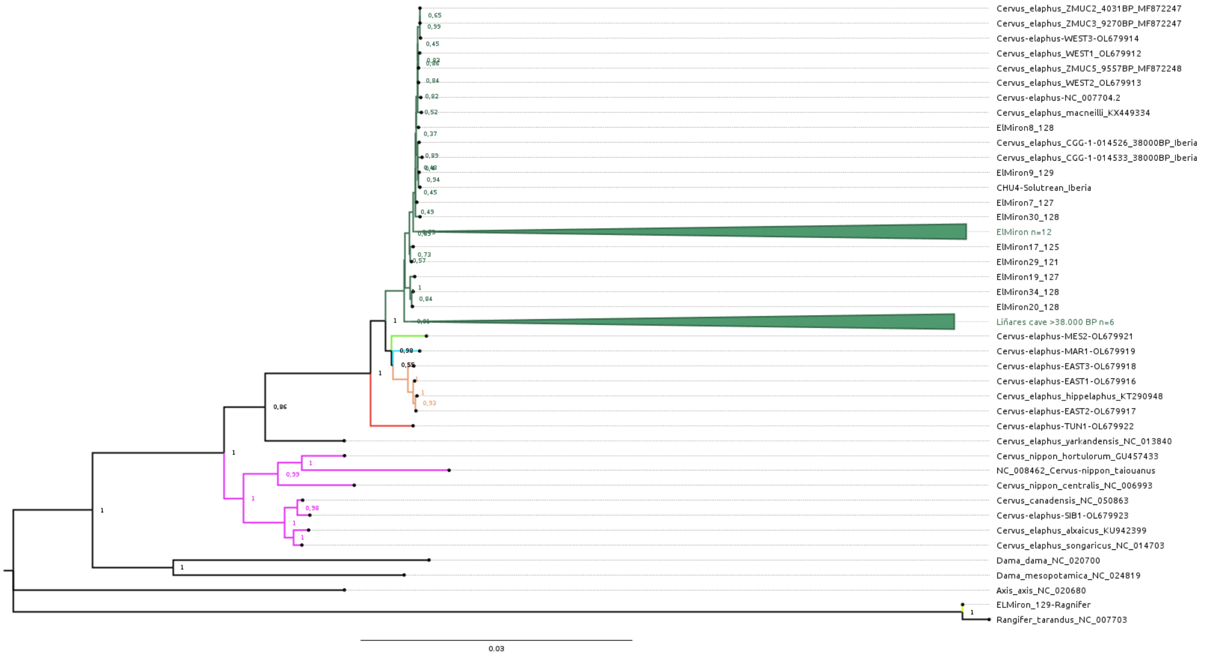

Supplementary Figure 26: Maximum likelihood tree of the Cervidae mtDNA genomes. The tree is rooted with *R. tarandus* mtDNA genomes. Colours denote clades. Dark green represents Western Clade A; pink represents the Eastern *Cervus* diversity; red represents Western Clade B; orange Western Clade C; light green Western Clade D, and light blue Western Clade E according to [130]. All the sequences from El Mirón are related to the rest of Clade A's diversity. It also shows the genetic proximity of El Mirón deer mtDNA sequences to the Pleistocene sequences from Liñares cave in Spain and the Solutrean bone tool from Chufín. Numbers in the nodes depict bootstrap values.

8. Rangifer tarandus

We analysed one mtDNA sedaDNA genome of *Rangifer tarandus* corresponding to ElMiron_21 (Supplementary Figure 27-28). We used the reference sequence NC_007703.1 to reconstruct the consensus sequence and individual alignments. The sequence is included in the *Cervidae* phylogeny (Supplementary Figure 26).

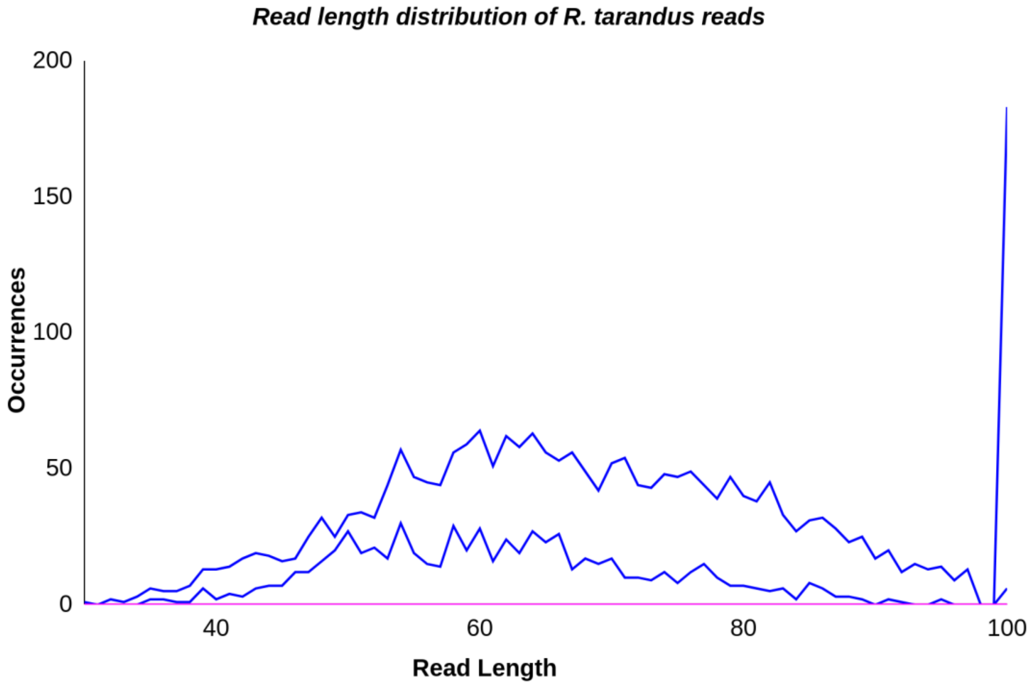

Supplementary Figure 27: Read length distribution of El Mirón *R. tarandus* mtDNA sedaDNA sequences. Black depicts the Initial Magdalenian 119.2 level, Red depicts the Solutrean 121-127 levels, Blue depicts the Gravettian 128 level, Green depicts the semi-sterile 129 level, and Purple depicts the Mousterian 130 level.

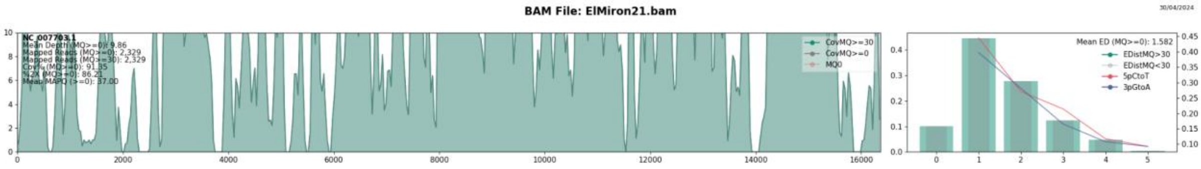

Supplementary Figure 28: Coverage and damage plots of *R. tarandus* sedaDNA mtDNA genome.

9. Rupicapra pyrenaica

We recovered five partial mtDNA genomes: ElMiron_1, ElMiron_2, ElMiron_14, ElMiron_7, and ElMiron_16 (Supplementary Figures 29-30). We used the reference sequence NC_0206331 to reconstruct the consensus sequences, we aligned the sequences from El Mirón with 35 other samples. When aligned, the five sequences from El Mirón show 37 variable sites.

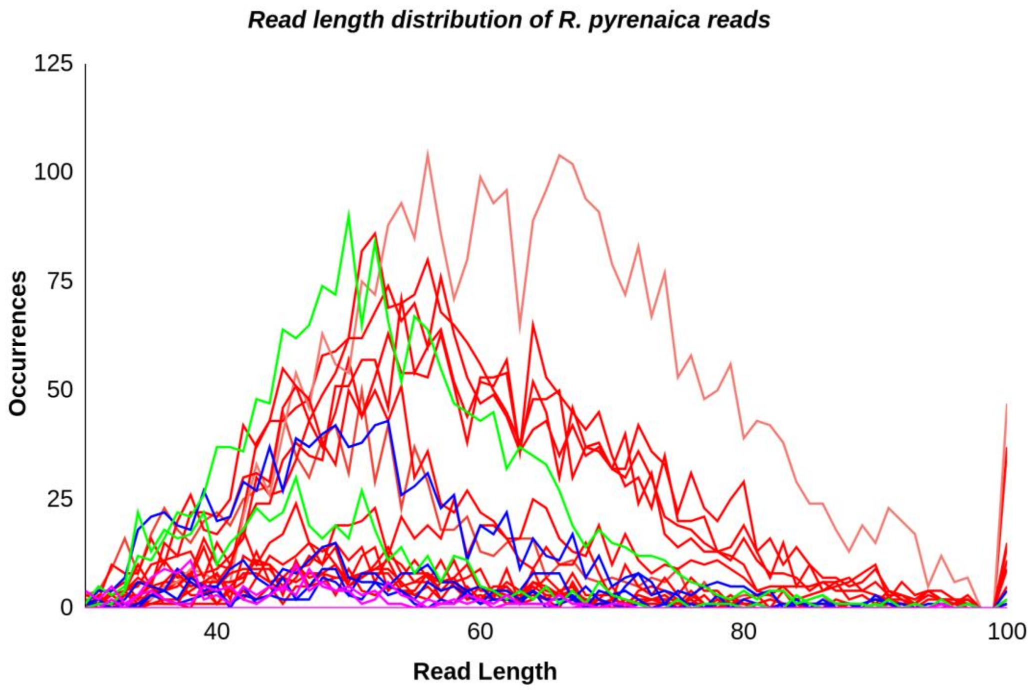

Supplementary Figure 29: Read length distribution of El Mirón *R. pyrenaica* genomes. Black depicts the Initial Magdalenian 119.2 level, Red depicts the Solutrean 121-127 levels, Blue depicts the Gravettian 128 level, Green depicts the semi-sterile 129 level, and Purple depicts the Mousterian 130 level.

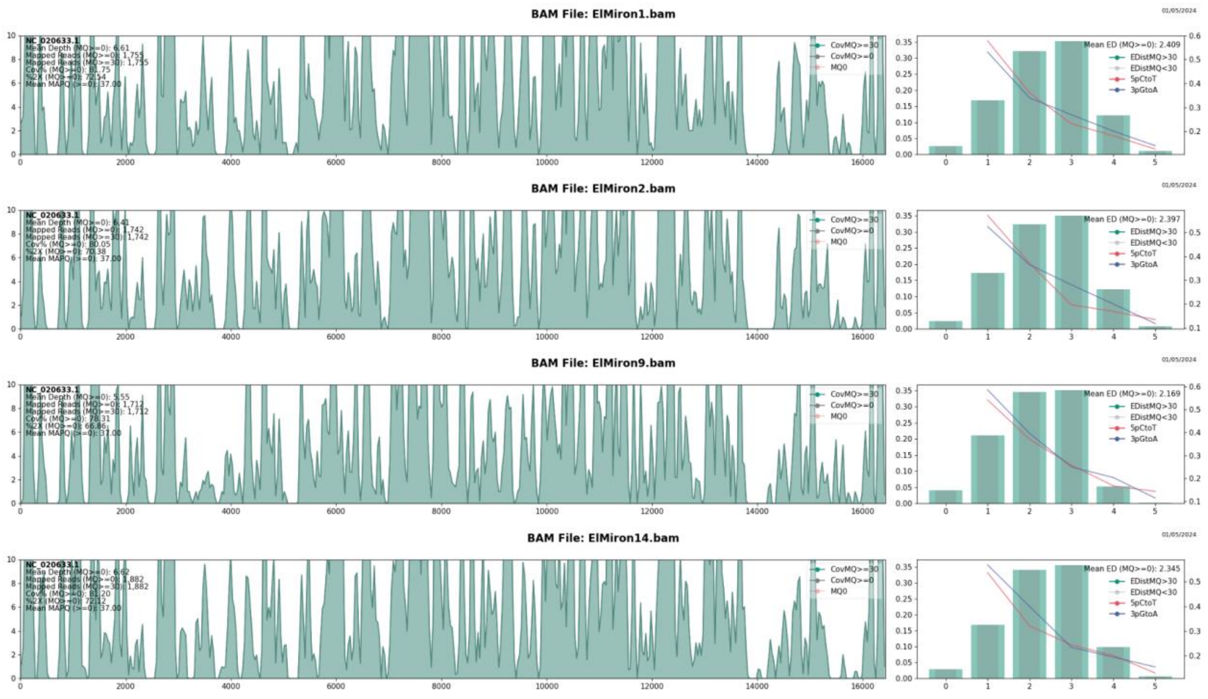

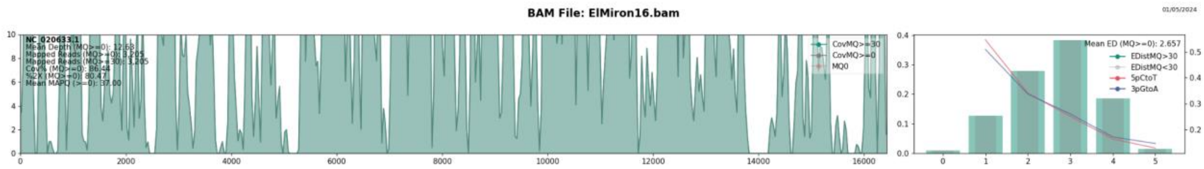

Supplementary Figure 30: read length distribution of El Mirón *R. pyrenaica* sedaDNA sequences.

10. Capra pyrenaica

We analysed 17 mtDNA sequences ElMiron_2, ElMiron_4, ElMiron_5, ElMiron_6, ElMiron_7, ElMiron_8, ElMiron_14, ElMiron_15 ,ElMiron_16, ElMiron_17, ElMiron_18, ElMiron_18 extracat 2, ElMiron_19, ElMiron_20, ElMiron_30, ElMiron_32, ElMiron_34 (Supplementary Figures 31-34). For reconstructing the consensus sequences and the individual alignments we used the reference sequence NC_020623.1 (alpine chamoix *Capra ibex)*. These sequences were aligned with the other 16 *Caprinae* sequences. The 17 sequences from El Mirón show 297 variable sites when aligned. As *C. pyrenaica* is one of the taxa with more genomes, we have tested the presence of a relationship between the coverage and the missing sites. We observe that with higher coverage, the amount of missing sites decreases (Supplementary Figure 32); in a relationship that is not lineal, we tested the significance of the correlation with Sperman's tests and the relationship is highly significant n=17, rho=-0.94 and *p-value*=4.79e−9. We can observe that there are regions of the genome not covered, which could indicate that the Pleistocene chamois present in Cantabria was differentiated from the reference used in this current analysis or that the capture baits designed with *Capra hircus* did not capture these regions. Despite these gaps, we are confident that these sequences resemble *C. pyrenaica* based on the unequivocally placing in the phylogeny (Supplementary Figure 33). The 21 sequences show 139 variable sites when aligned.

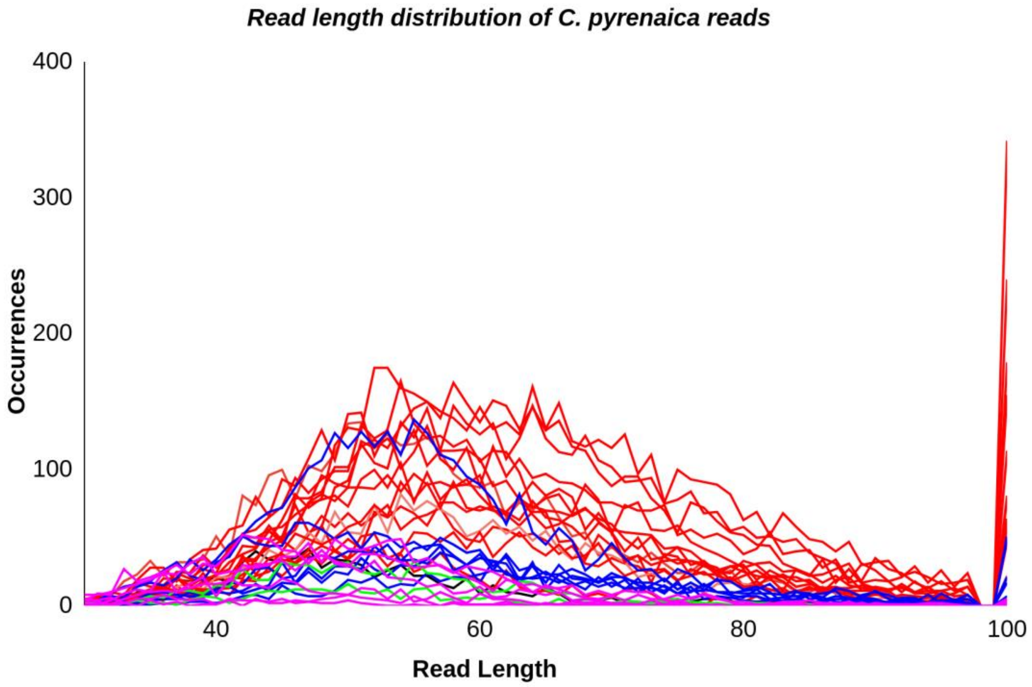

Supplementary Figure 31: Read length distribution of El Mirón *C. pyrenaica* sequences. Black depicts Initial Magdalenian 119.2 level, Red depicts Solutrean 121-127 levels, Blue depicts Gravettian 128 level, Green depicts semi-sterile 129 level, Purple depicts Mousterian 130 level.

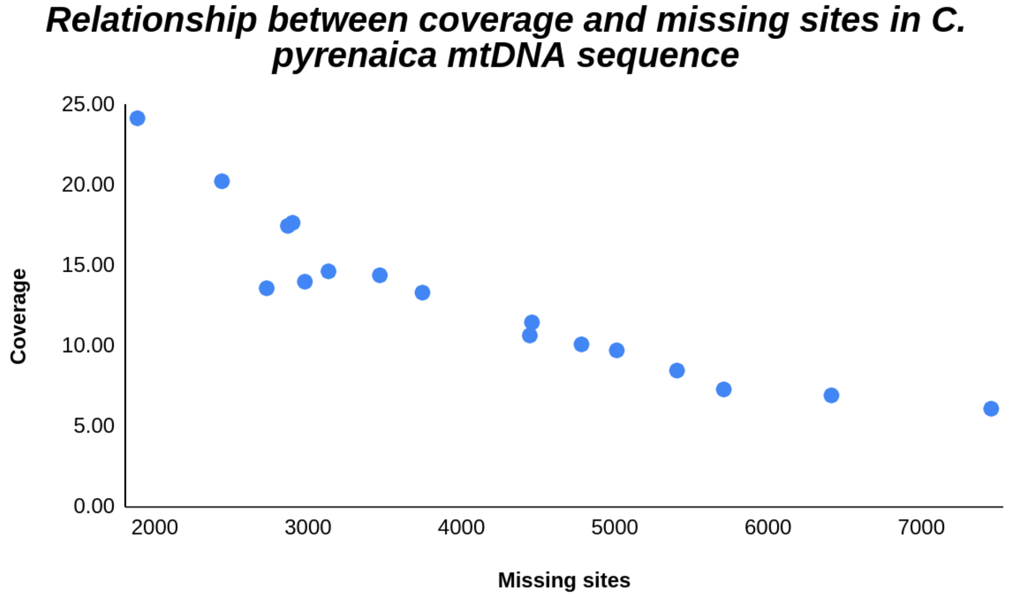

Supplementary Figure 32: Relationship between missing sites and coverage in the reads attributed to *C. pyrenaica* from El Mirón.

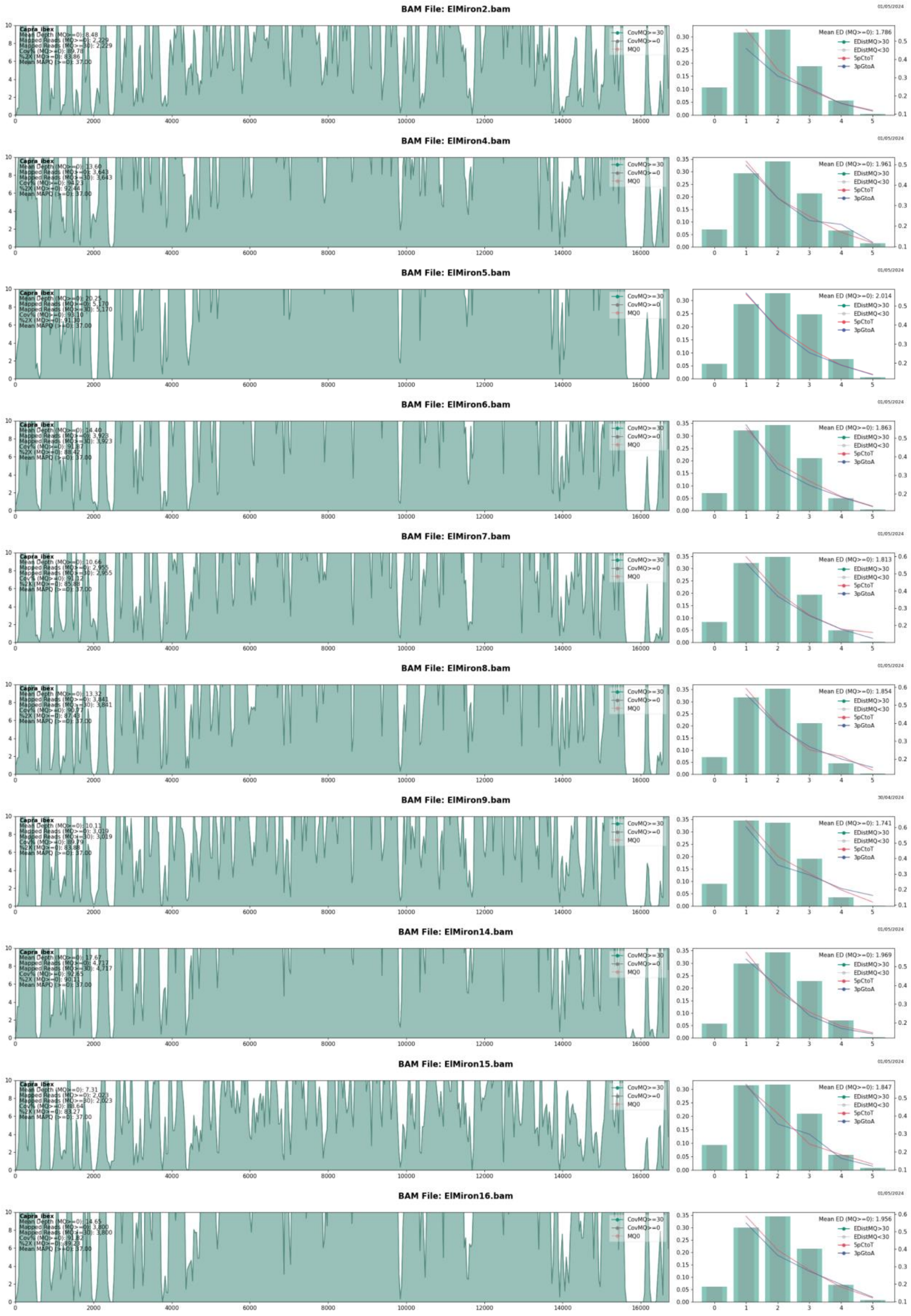
BAM File: ElMiron2.bam
01/05/2024
Capra_ibex
CovMQ>=30
CovMQ>=0
MQ0
Mean ED (MQ>=0): 1.786
EDistMQ>30
EDistMQ<30
5pCtoT
3pGtoA
BAM File: ElMiron4.bam
01/05/2024
Mean ED (MQ>=0): 1.961
BAM File: ElMiron5.bam
01/05/2024
Mean ED (MQ>=0): 2.014
BAM File: ElMiron6.bam
01/05/2024
Mean ED (MQ>=0): 1.863
BAM File: ElMiron7.bam
01/05/2024
Mean ED (MQ>=0): 1.813
BAM File: ElMiron8.bam
01/05/2024
Mean ED (MQ>=0): 1.854
BAM File: ElMiron9.bam
30/04/2024
Mean ED (MQ>=0): 1.741
BAM File: ElMiron14.bam
01/05/2024
Mean ED (MQ>=0): 1.969
BAM File: ElMiron15.bam
01/05/2024
Mean ED (MQ>=0): 1.847
BAM File: ElMiron16.bam
01/05/2024
Mean ED (MQ>=0): 1.956

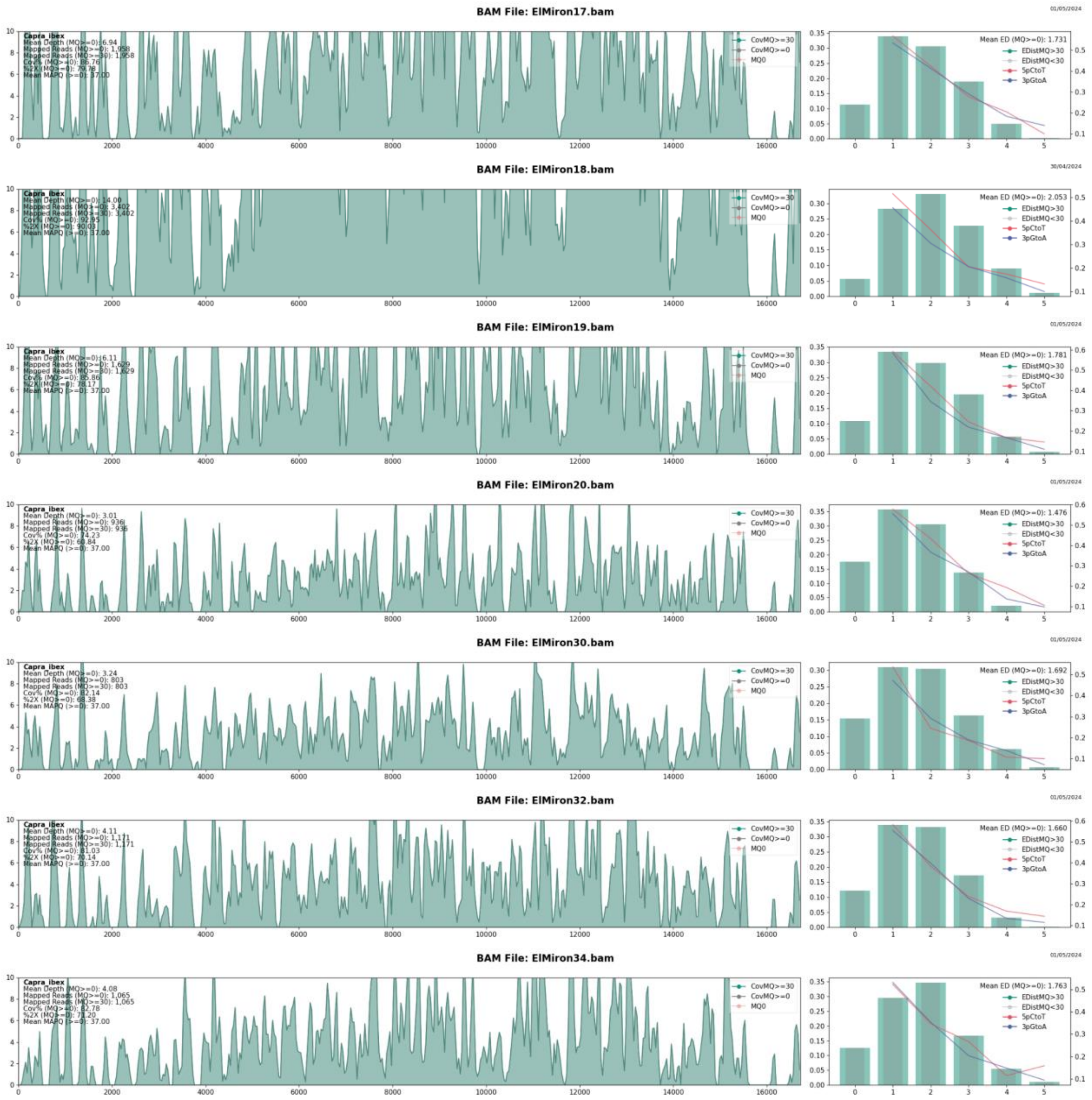

Supplementary Figure 33: Coverage plots and damage plots of *C. pyrenaica* sedaDNA genomes.

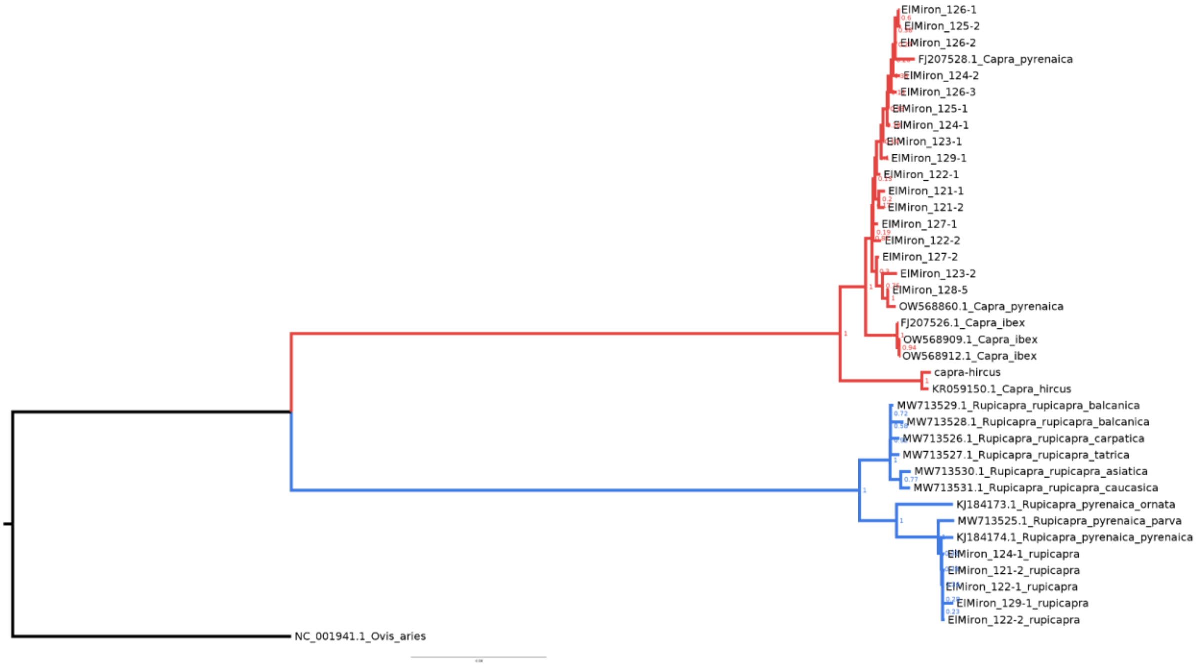

Supplementary Figure 34: Maximum likelihood tree of the *Caprinae mtDNA* genomes. The red colour denotes the *Capra* mtDNA genomes, and the blue ones represent the *Rupicapra* mtDNA genomes. The phylogeny has been rooted with *O. aries* mtDNA sequence. The Phylogeny confirms that the mtDNA sequences from El Mirón belong to *C. pyrenaica and R. pyrenaica.* Node numbers denote bootstrap values.

11. Equus sp.

We analysed one mtDNA partial genome ElMiron_9 (Supplementary Figures 35-37). For reconstructing the consensus sequences as well as the individual alignments we used the reference sequence NC_001640.1. We aligned the sequence from ElMiron_9 sample with other 106 ancient and modern horse sequences plus an outgroup.

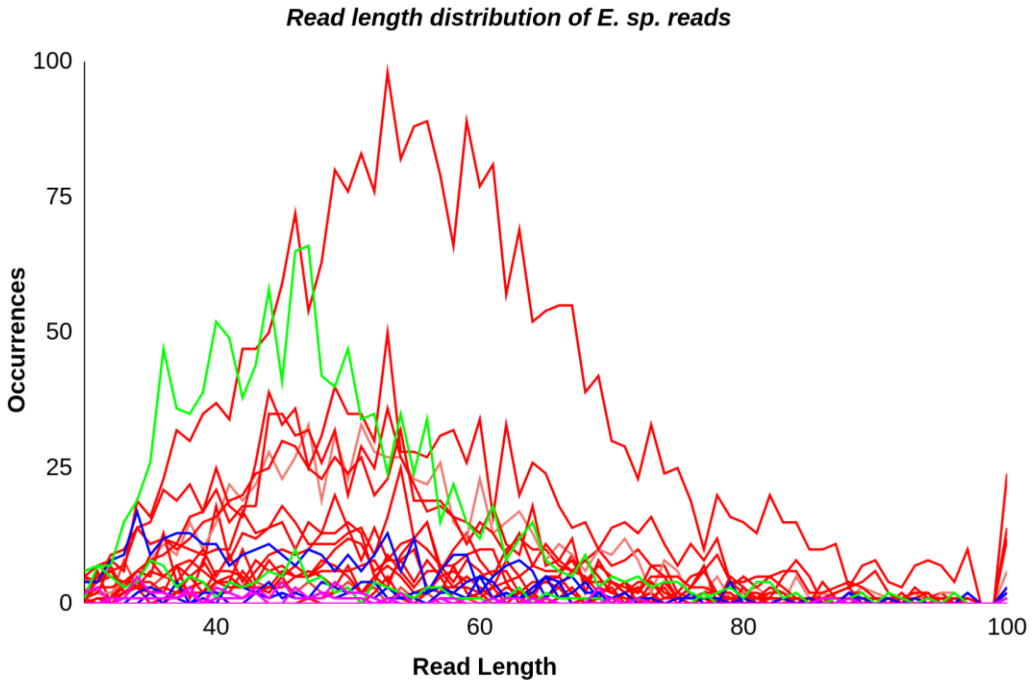

Supplementary Figure 35: read length distribution of El Mirón *E.* sp*.* sequences.

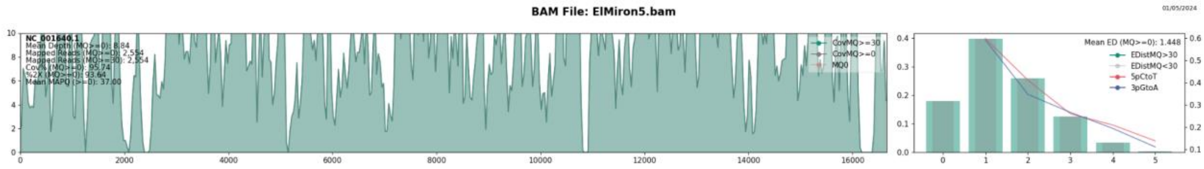

Supplementary Figure 36: read length distribution of El Mirón *Equus.* sp*.* sedaDNA mtDNA genomes.

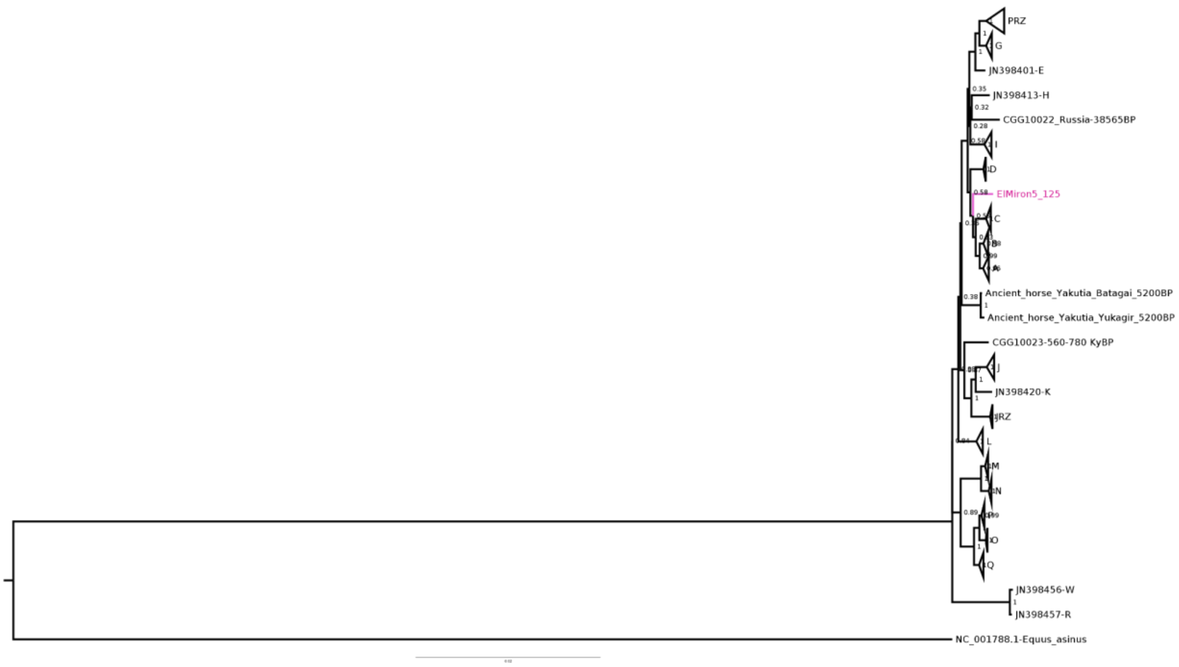

Supplementary Figure 37: Maximum likelihood tree of the *Equus* sp. *mtDNA* genomes. Purple denotes the Mirón5_125 mtDNA sedaDNA sequence. This sequence appears within the present-day and ancient Eurasian diversity as a separate clade.

We have not analysed the individual genomes for the rest of the species we identified in Supplementary Note 3. Still, we have aligned the reads against the reference sequence (Supplementary Table 6), and we present the read length distribution in Supplementary Figure 38. The deamination values are present in Supplementary Data 3.

Supplementary Table 6: Reference mtDNA genomes used for the species not described before

Taxa	Reference used
C. capreolus	NC_020684.1
O. aries	NC_001941.1
S. aluco	NC_072569.1
C. corax	NC_034838.1
T. europaea	NC_002391.1
C. livia	NC_013978.1
S. scrofa	NC_000845.1
M. arvalis	NC_038176.1
F. catus	NC_001700.1
B. bonasus	NC_006853.1
B. primigenius	NC_013996.1
L. europaeus	NC_004028.1
S. araneus	NC_027963.1
P. graculus	NC_025927.1
F. tinnunculus	NC_011307.1
M. nivalis	NC_020639.1
L. pardinus	NC_028319.1
C. antiquitatis	FJ905813.1
M. primigenius	NC_007596.2

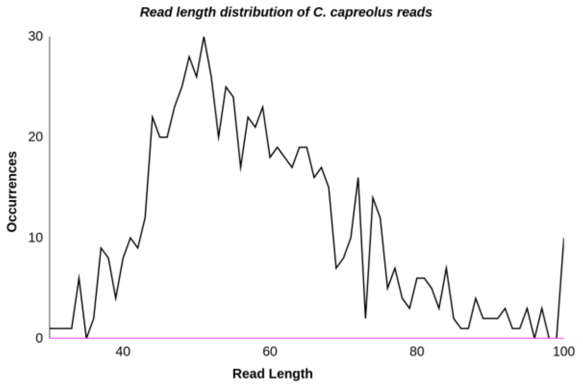
Read length distribution of C. capreolus reads
Occurrences
0
10
20
30
40
60
80
100
Read Length

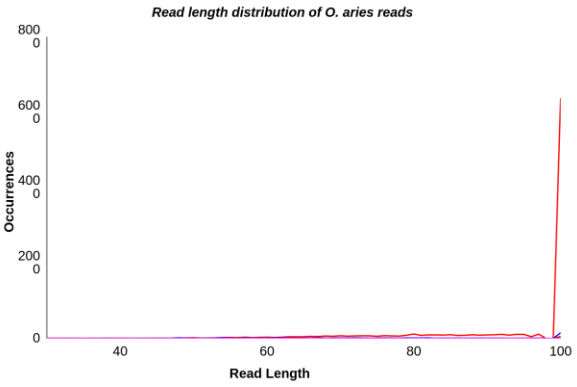
Read length distribution of O. aries reads
Occurrences
0
200
0
400
0
600
0
800
0
40
60
80
100
Read Length

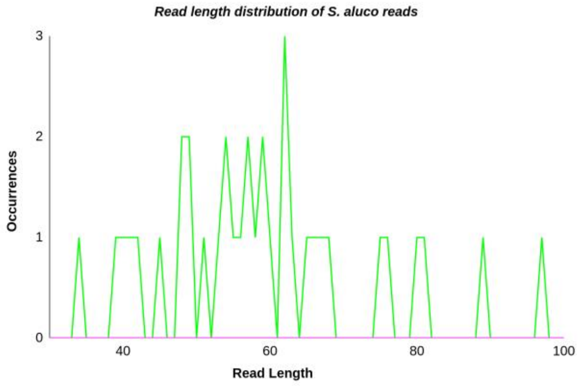
Read length distribution of S. aluco reads
Occurrences
0
1
2
3
40
60
80
100
Read Length

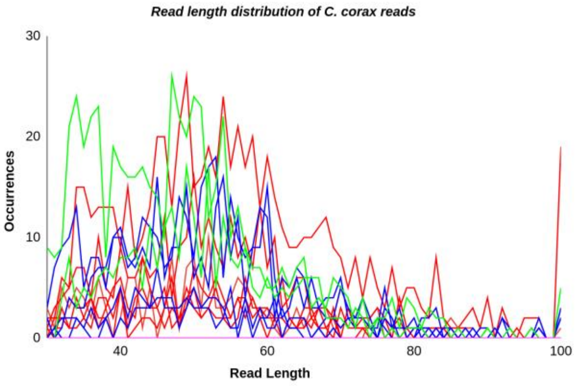
Read length distribution of C. corax reads
Occurrences
0
10
20
30
40
60
80
100
Read Length

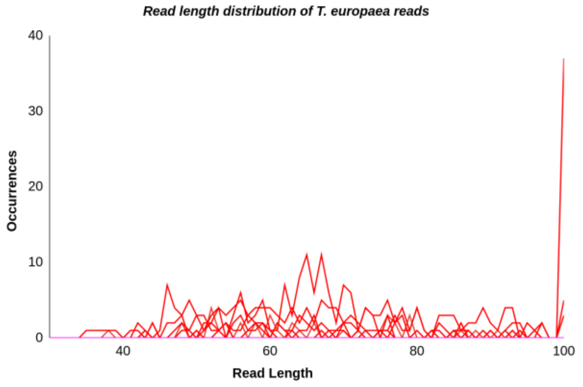
Read length distribution of T. europaea reads
Occurrences
0
10
20
30
40
40
60
80
100
Read Length

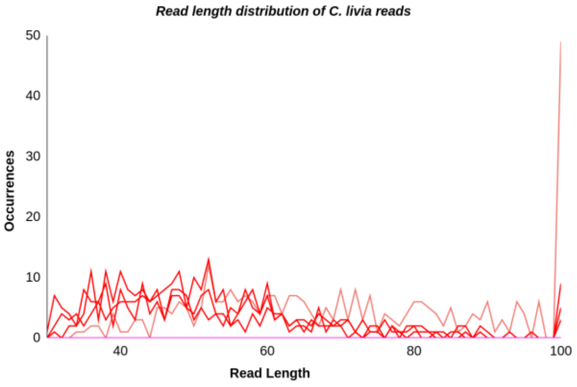
Read length distribution of C. livia reads
Occurrences
0
10
20
30
40
50
40
60
80
100
Read Length

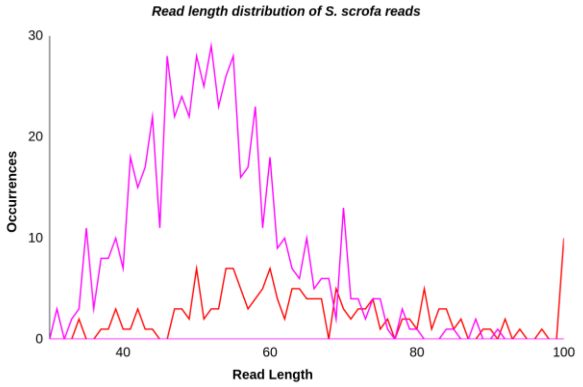
Read length distribution of S. scrofa reads
Occurrences
0
10
20
30
40
60
80
100
Read Length

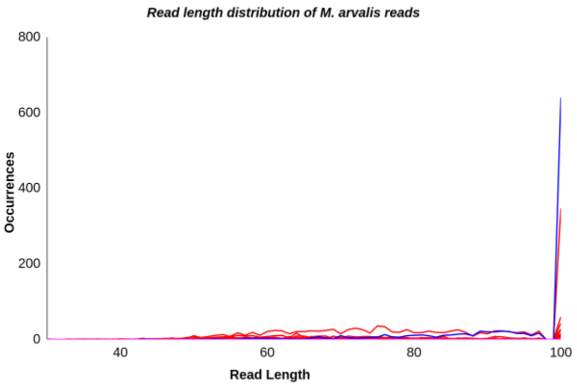
Read length distribution of M. arvalis reads
Occurrences
0
200
400
600
800
40
60
80
100
Read Length

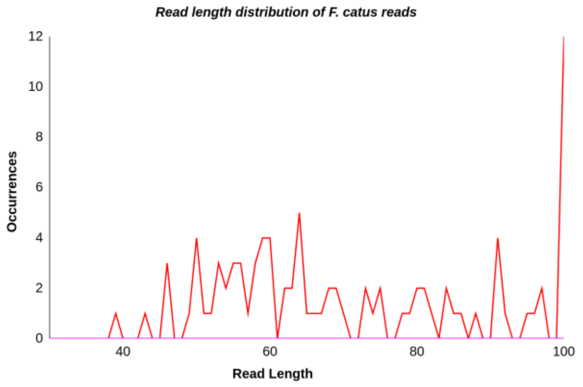
Read length distribution of F. catus reads
Occurrences
Read Length
0 2 4 6 8 10 12
40 60 80 100

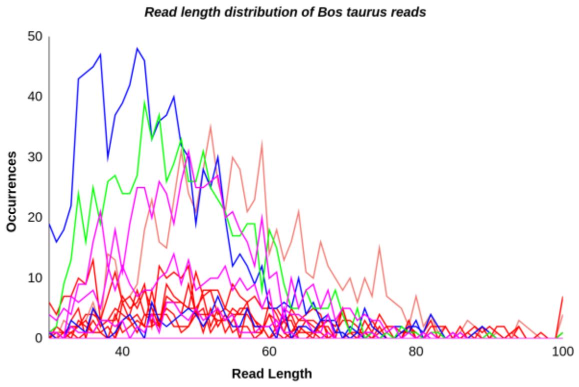
Read length distribution of Bos taurus reads
Occurrences
Read Length
0 10 20 30 40 50
40 60 80 100

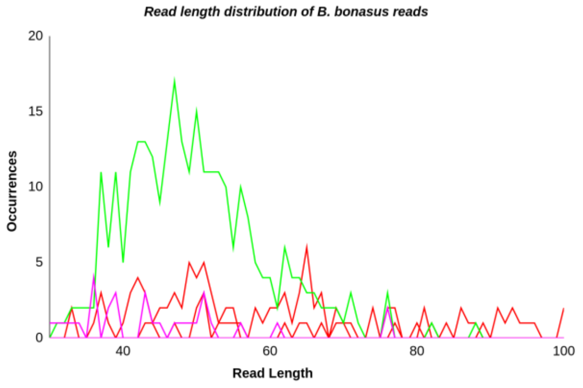
Read length distribution of B. bonasus reads
Occurrences
Read Length
0 5 10 15 20
40 60 80 100

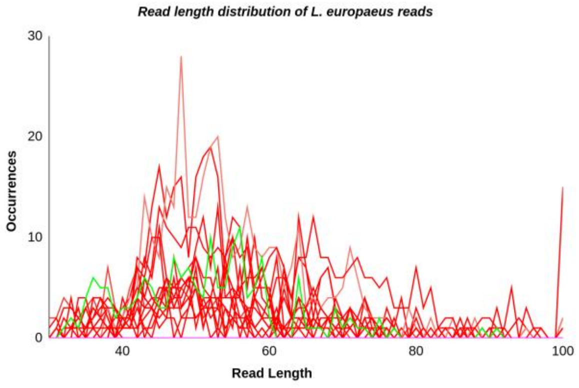
Read length distribution of L. europaeus reads
Occurrences
Read Length
0 10 20 30
40 60 80 100

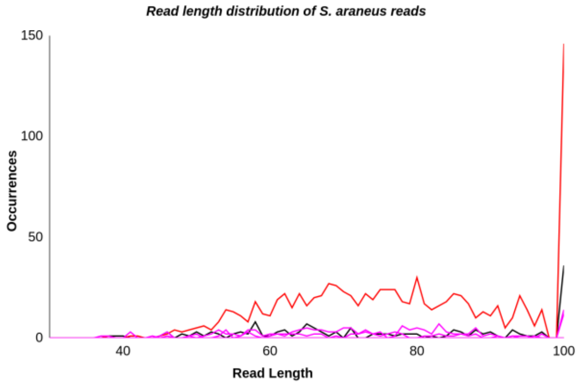
Read length distribution of S. araneus reads
Occurrences
Read Length
0 50 100 150
40 60 80 100

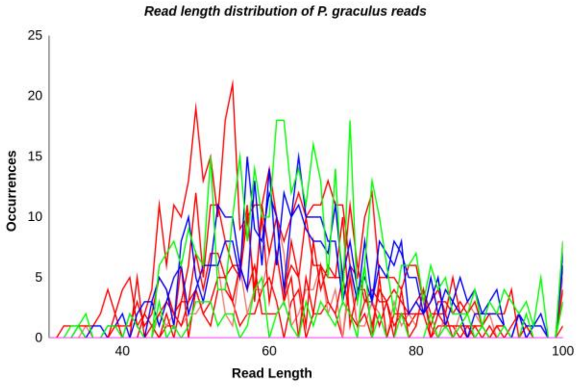
Read length distribution of P. graculus reads
Occurrences
Read Length
0 5 10 15 20 25
40 60 80 100

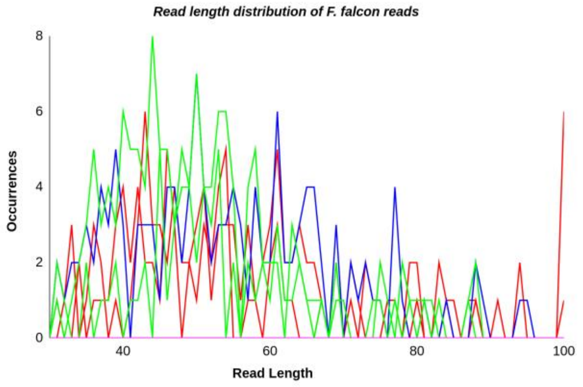
Read length distribution of F. falcon reads
Occurrences
Read Length
0 2 4 6 8
40 60 80 100

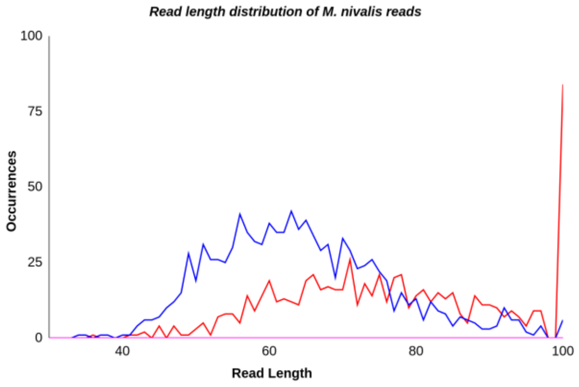
Read length distribution of M. nivalis reads
Occurrences
Read Length
0 25 50 75 100
40 60 80 100

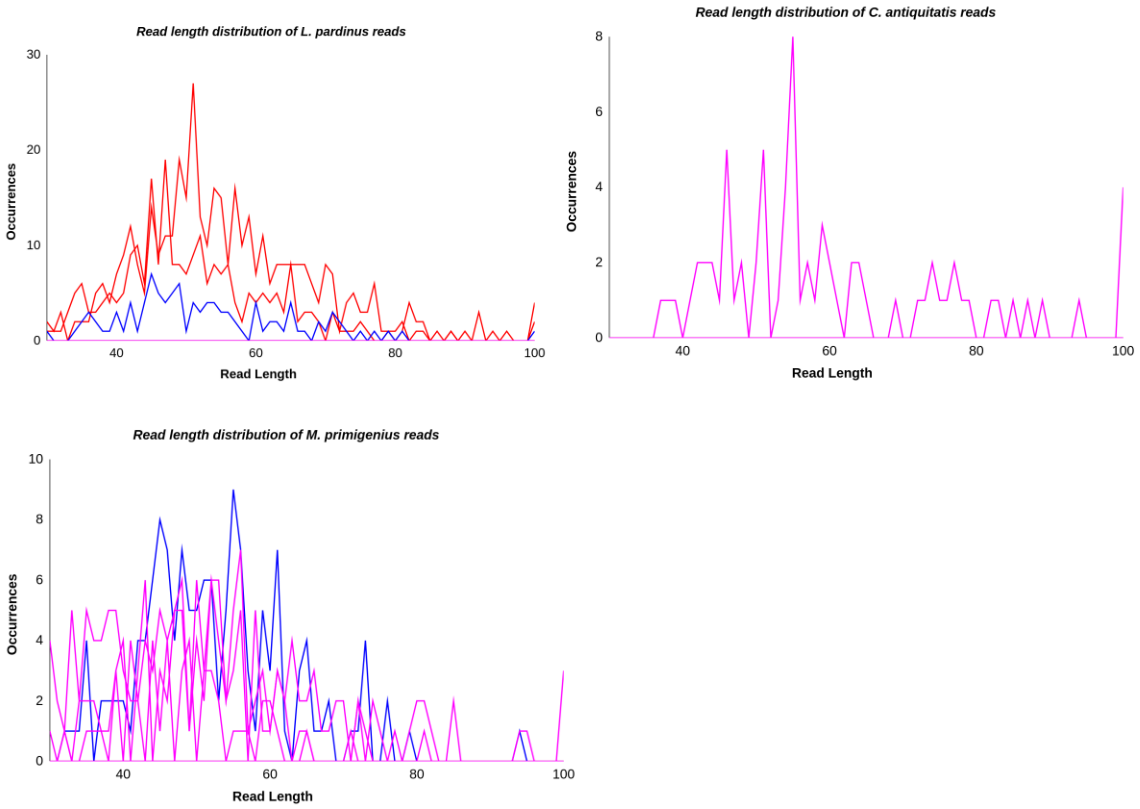

Supplementary Figure 38: Read length distribution of the taxa that have been individually aligned. Black depicts the Initial Magdalenian 119.2 level, Red depicts Solutrean 121-127 levels, Blue depicts the Gravettian 128 level, Green depicts the semi-sterile 129 level, and Purple depicts the Mousterian 130 level.

12. Human analyses

Human DNA reads were analysed with mapDamage2-2.2.2 to check for deamination as we did for the rest of analyzed taxa. We later calculated the amount of contamination and determined the presence of multiple sequences using Schmutzi 2.0 [157] (with and without prediction of contamination) and Calico [158]. Calico does not distinguish between modern or ancient reads but enables us to determine the proportions of the sequence donors, assessing the presence of multiple individual sequences mixed in our reads. We later generated the mtDNA sedaDNA genome consensus sequences with Schmutzi and checked the positions individually using IGV 2.16.1 genome viewer. We also determined the mtDNA haplogroup with haplogrep 3.0 [159]. We aligned the resulting sequences with a dataset comprising modern and ancient sequences (Supplementary Data 12) using MAFFT 7.52 [57]. We produced Maximum Likelihood trees using MEGA 10.2.4 with a partial deletion of 95%, GTR substitution model and 500 bootstrap replicates[58].

We detected the presence of human reads in all samples but only 10 with deamination values greater than 30%, which we consider the ones showing real Pleistocene DNA. Only ElMiron_1, ElMiron_14 and ElMiron_18 showed enough reads to assess the relationship of the mtDNA sequences with other ancient and modern ones. None of these samples corresponds to level 130, not detecting the presence of human DNA linked to Mousterian archaeological culture in El Mirón.

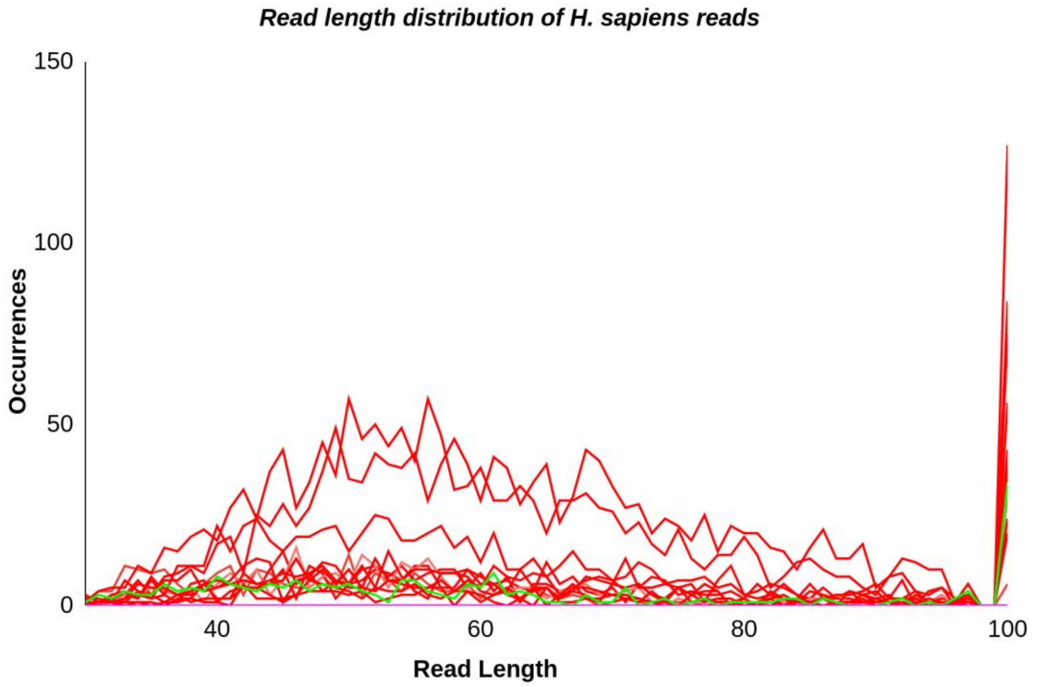

Supplementary Figure 39: read length distribution of El Mirón *H. sapiens* sequences. Black depicts the Initial Magdalenian 119.2 level, Red depicts Solutrean 121-127 levels, Blue depicts the Gravettian 128 level, Green depicts the semi-sterile 129 level, and Purple depicts the Mousterian 130 level.

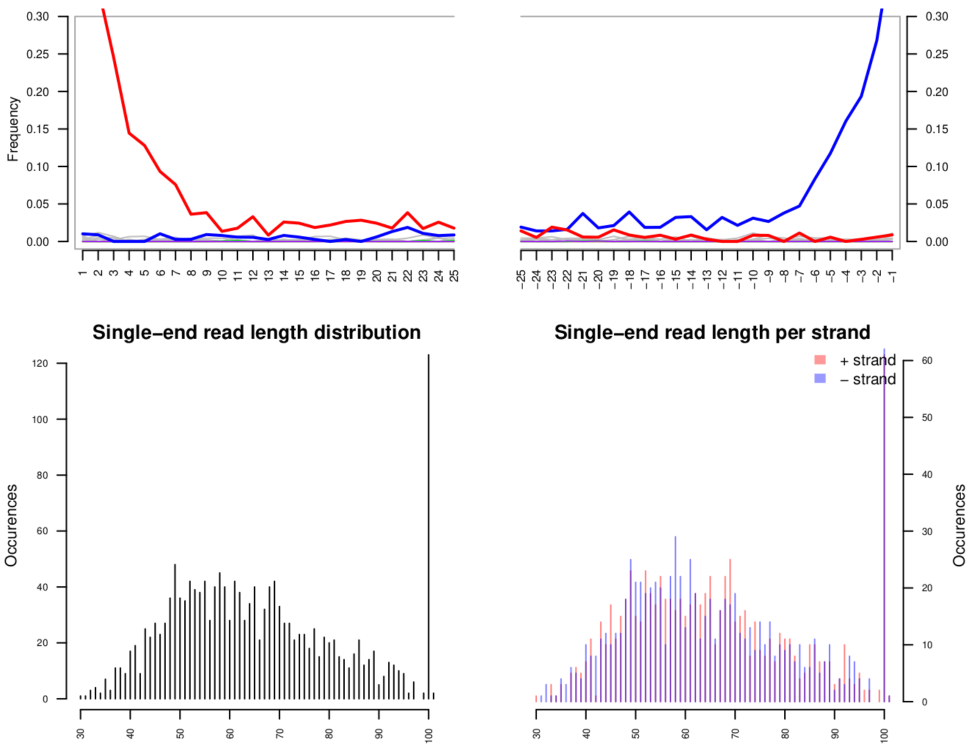

Supplementary Figure 40: Read length distribution and deamination pattern of El Miron18 (Level 126) sample human reads.

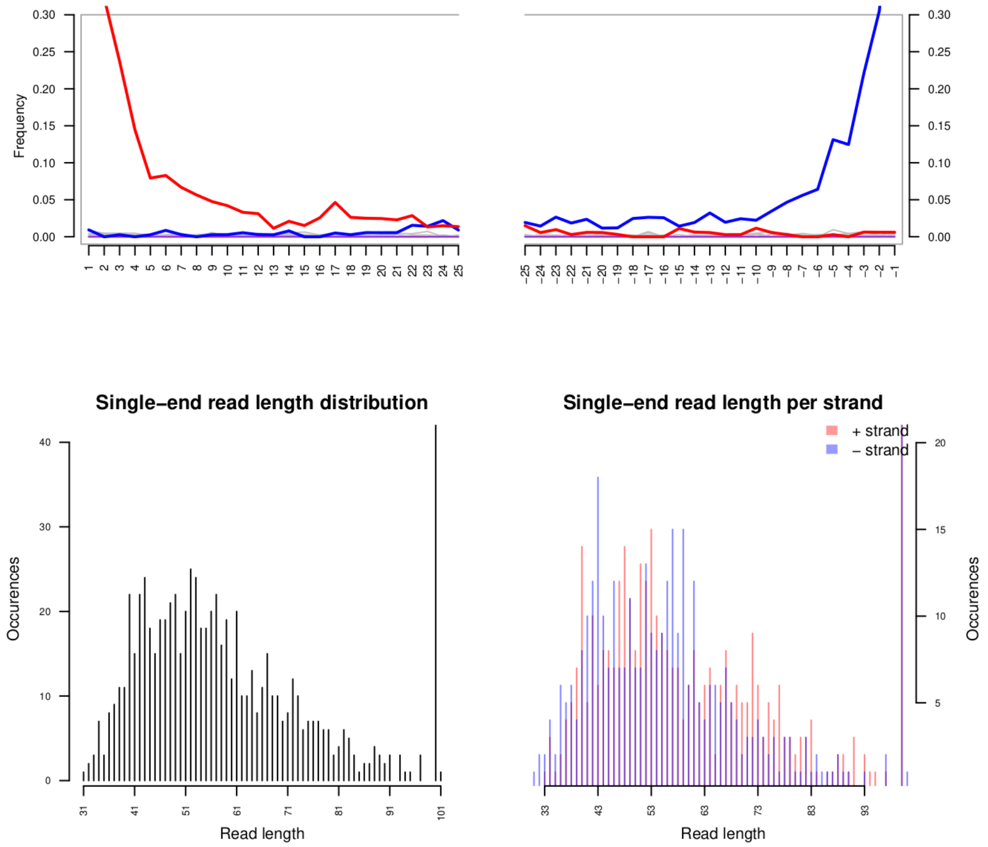

Supplementary Figure 41: Read length distribution and deamination pattern of El Miron1 (Level 121) sample human reads.

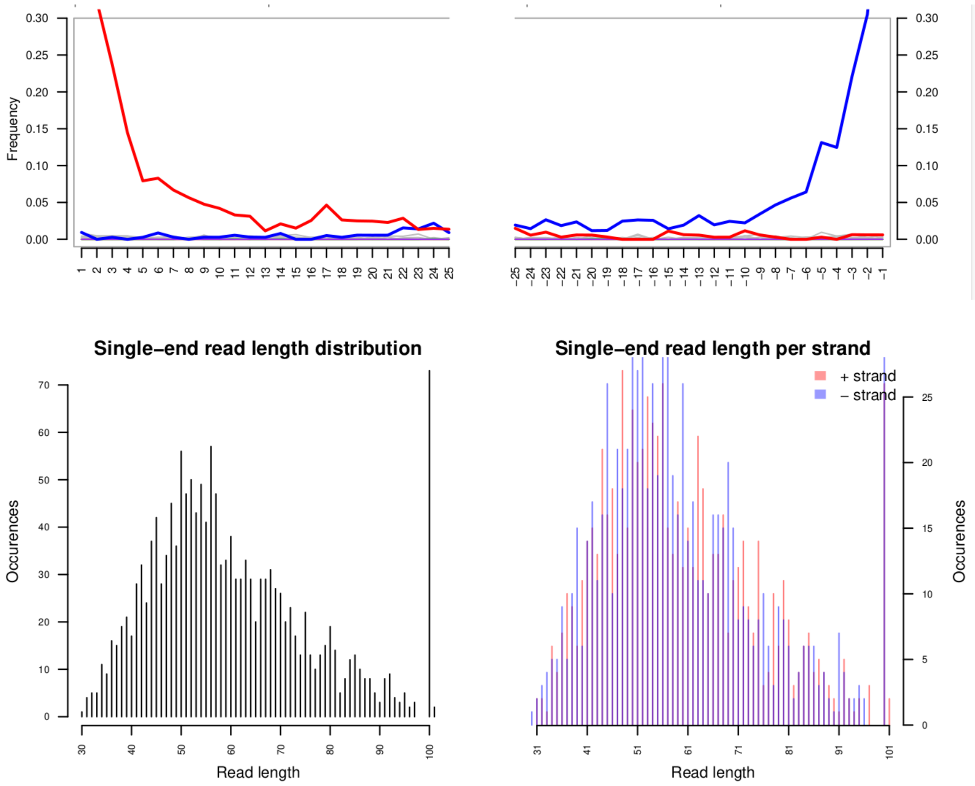

Supplementary Figure 42: Read length distribution and deamination pattern of El Miron14 (Level 122) sample human reads.

Supplementary References

1. Marín-Arroyo, A. B. *et al.* Seasonality of Human Occupations in El Mirón Cave: Late Upper Paleolithic Hunter-Gatherer Settlement-Subsistence Systems in Cantabrian Spain. *Journal of Paleolithic Archaeology* **6**, 7 (2023).
2. Cuenca-Bescós, G., Marín-Arroyo, A. B. & Martínez, I. Relationship between Magdalenian subsistence and environmental change: The mammalian evidence from El Mirón (Spain). *Quaternary* (2012).
3. Hopkins, R. J. A., Straus, L. G. & González Morales, M. R. Assessing the chronostratigraphy of el Mirón cave, Cantabrian Spain. *Radiocarbon* **63**, 821–852 (2021).
4. Straus, L. G. & González Morales, M. R. *El Mirón Cave, Cantabrian Spain: The Site and Its Holocene Archaeological Record*. (University of New Mexico Press, 2012).
5. Straus, L. G. & González Morales, M. R. New Dates for the Solutrean and Magdalenian of Cantabrian Spain: El Miron and La Riera Caves. *Radiocarbon* **60**, 1013–1016 (2018).
6. Straus, L. G. & González Morales, M. R. The Upper Paleolithic sequence in el Mirón Cave (Ramales de la Victoria, Cantabria, Spain): An overview. *Journal of Archaeological Science: Reports* **27**, 101998 (2019).
7. Straus, L. G., González Morales, M. R., Carretero, J. M. & Marín-Arroyo, A. B. 'The Red Lady of El Mirón'. Lower Magdalenian life and death in Oldest Dryas Cantabrian Spain: an overview. *J. Archaeol. Sci.* **60**, 134–137 (2015).
8. Fu, Q. *et al.* The genetic history of Ice Age Europe. *Nature* **534**, 200–205 (2016).
9. Posth, C. *et al.* Palaeogenomics of Upper Palaeolithic to Neolithic European hunter-gatherers. *Nature* **615**, 117–126 (2023).
10. Marín-Arroyo, A. B. *et al.* The Middle to Upper Palaeolithic transition at El Mirón

Cave (Cantabria, Spain). *Quat. Int.* **544**, 23–31 (2020).

11. Farrand, W. Sedimentology of el Mirón cave. in *El Mirón Cave, Cantabrian Spain: The Site and Its Holocene Archaeological Record* (eds. Straus, L. G. & González Morales, M. R.) 60–94 (University of New Mexico Press, 2012).
12. Straus, L. G., González Morales, M., Farrand, W. R. & Hubbard, W. J. Sedimentological and stratigraphic observations in El Mirón, a late Quaternary cave site in the Cantabrian Cordillera, Northern Spain. *Geoarchaeology* **16**, 603–630 (2001).
13. Geiling, J. M. Human ecodynamics in the Late Upper Pleistocene of Northern Spain: an archeozoological study of ungulate remains from the Lower Magdalenian and other periods …. (University of Cantabria, 2020).
14. Sanguino, J. & Montes, R. *La cueva del 'El Pendo' : actuaciones arqueológicas 1994-2000*. (Consejería de Cultura, Educación y Deporte, 2001).
15. Montes, R., Sanguino, J., Martín, P., Gómez, A. J. & Morcillo, C. La secuencia estratigráfica de la cueva de El Pendo (Escobedo de Camargo, Cantabria): problemas geoarqueológicos de un referente cronocultural. *Geoarqueología y patrimonio en la Península Ibérica y el entorno mediterráneo, ADEMA, Almazán (Soria)* 127–138 (2005).
16. Kehl, M. *et al.* Towards a revised stratigraphy for the Middle to Upper Palaeolithic boundary at La Güelga (Narciandi, Asturias, Spain). Soil micromorphology and new radiocarbon data. *Bol. geol. min.* **129**, 183–206 (2018).
17. González-Morales, M. & Straus, L. G. La ocupación gravetiense de la cueva de El Mirón (Ramales de la Victoria, Cantabria) y el contexto del arte paleolítico temprano de la cuenca del Asón. in *Pensando el Gravetiense: nuevos datos para la Región Cantábrica, en su contexto peninsular y pirenaico* (eds. de las Heras Martín, C., Lasheras Corruchaga, J. A., Arrizabalaga Valbuena, A. & de la Rasilla Vives, M.)

289–299 (Ministerio de CulturaEditor: Monografías del Museo y Centro de Investigación de Altamira, 2012).

18. Straus, L. G. & González Morales, M. R. *El Mirón Cave, Cantabrian Spain: The Site and Its Holocene Archaeological Record*. (University of New Mexico Press, 2012).
19. Marín-Arroyo, A. B. *Arqueozoología En El Cantábrico Oriental Durante La Transición Pleistoceno/holoceno: La Cueva Del Mirón*. (PUbliCan, Ediciones de la Universidad de Cantabria, 2010).
20. Dabney, J. *et al.* Complete mitochondrial genome sequence of a Middle Pleistocene cave bear reconstructed from ultrashort DNA fragments. *Proc. Natl. Acad. Sci. U. S. A.* **110**, 15758–15763 (2013).
21. Korlević, P. *et al.* Reducing microbial and human contamination in DNA extractions from ancient bones and teeth. *Biotechniques* **59**, 87–93 (2015).
22. Meyer, M. & Kircher, M. Illumina sequencing library preparation for highly multiplexed target capture and sequencing. *Cold Spring Harb. Protoc.* **2010**, db.prot5448 (2010).
23. Kircher, M., Sawyer, S. & Meyer, M. Double indexing overcomes inaccuracies in multiplex sequencing on the Illumina platform. *Nucleic Acids Res.* **40**, e3 (2012).
24. Tejero, J.-M. *et al.* Cervidae antlers exploited to manufacture Prehistoric tools and hunting implements as a reliable source of ancient DNA. *Heliyon* e31858 (2024).
25. Rohland, N. *et al.* Three assays for in-solution enrichment of ancient human DNA at more than a million SNPs. *Genome Res.* **32**, 2068–2078 (2022).
26. Martin, M. Cutadapt removes adapter sequences from high-throughput sequencing reads. *EMBnet.journal* **17**, 10–12 (2011).
27. Myers, E. W. The fragment assembly string graph. *Bioinformatics* **1;21 Suppl 2**, 79–85 (2005).

28. Hannon, G. J. *FASTX-Toolkit*. (2010).

29. Vogel, N. A. *et al.* euka: Robust tetrapodic and arthropodic taxa detection from modern and ancient environmental DNA using pangenomic reference graphs. *Methods Ecol. Evol.* (2023) doi:10.1111/2041-210x.14214.

30. Li, H. *et al.* The Sequence Alignment/Map format and SAMtools. *Bioinformatics* **25**, 2078–2079 (2009).

31. Altschul, S. F., Gish, W., Miller, W., Myers, E. W. & Lipman, D. J. Basic local alignment search tool. *J. Mol. Biol.* **215**, 403–410 (1990).

32. Huson, D. H., Auch, A. F., Qi, J. & Schuster, S. C. MEGAN analysis of metagenomic data. *Genome Res.* **17**, 377–386 (2007).

33. Li, H. & Durbin, R. Fast and accurate short read alignment with Burrows-Wheeler transform. *Bioinformatics* **25**, 1754–1760 (2009).

34. Picard-tools. http://broadinstitute.github.io/picard.

35. Jónsson, H., Ginolhac, A., Schubert, M., Johnson, P. L. F. & Orlando, L. mapDamage2.0: fast approximate Bayesian estimates of ancient DNA damage parameters. *Bioinformatics* **29**, 1682–1684 (2013).

36. Ramírez, F. *et al.* deepTools2: a next generation web server for deep-sequencing data analysis. *Nucleic Acids Res.* **44**, W160–5 (2016).

37. Team, R. S. RStudio: integrated development for R. RStudio. *Inc., Boston, MA* (2015).

38. Narasimhan, V. *et al.* BCFtools/RoH: a hidden Markov model approach for detecting autozygosity from next-generation sequencing data. *Bioinformatics* **32**, 1749–1751 (2016).

39. Korneliussen, T. S., Albrechtsen, A. & Nielsen, R. ANGSD: Analysis of Next Generation Sequencing Data. *BMC Bioinformatics* **15**, 356 (2014).

40. Robinson, J. T. *et al.* Integrative genomics viewer. *Nat. Biotechnol.* **29**, 24–26 (2011).

41. Danecek, P., McCarthy, S. & Li, H. bcftools—utilities for variant calling and manipulating vcfs and bcfs. *The MIT/Expat License or GPL License, see the* (2015).
42. Gansauge, M.-T. & Meyer, M. Single-stranded DNA library preparation for the sequencing of ancient or damaged DNA. *Nat. Protoc.* **8**, 737–748 (2013).
43. Ripoll, M. P., Morales Pérez, J. V., Sanchis Serra, A., Aura Tortosa, J. E. & Montañana, I. S. Presence of the genus Cuon in upper Pleistocene and initial Holocene sites of the Iberian Peninsula: new remains identified in archaeological contexts of the Mediterranean region. *J. Archaeol. Sci.* **37**, 437–450 (2010).
44. Lloveras, L. *et al.* The role of birds in Upper Palaeolithic sites: Zooarchaeological and taphonomic analysis of the avian remains from Arbreda Cave (Serinyà, northeast Iberia). *Quat. Int.* **626-627**, 22–32 (2022).
45. Nabais, M., Pimenta, C. & Zilhão, J. Human-bird interaction in last Interglacial Iberia: A combined approach using skeletal part analysis, bone surface modification, bird ethology and ethnography. *Journal of Archaeological Science: Reports* **49**, 104023 (2023).
46. Sommer, R. S. & Benecke, N. Late Pleistocene and Holocene development of the felid fauna (Felidae) of Europe: a review. *J. Zool.* **269**, 7–19 (2006).
47. Finlayson, S. & Finlayson, C. The birdmen of the Pleistocene: On the relationship between Neanderthals and scavenging birds. *Quat. Int.* **421**, 78–84 (2016).
48. Gómez-Olivencia, A. *et al.* New evidence for the presence of reindeer (*Rangifer tarandus*) on the Iberian Peninsula in the Pleistocene: an archaeopalaeontological and chronological reassessment. *Boreas* **43**, 286–308 (2014).
49. Sanz-Royo, A., Terlato, G. & Marín-Arroyo, A. B. Taphonomic data from the transitional Aurignacian of El Castillo cave (Spain) reveals the role of carnivores at the Aurignacian Delta level. *Quaternary Science Advances* **13**, 100147 (2024).

50. Álvarez-Lao, D. J. & García, N. Comparative revision of the Iberian woolly mammoth (Mammuthus primigenius) record into a European context. *Quat. Sci. Rev.* **32**, 64–74 (2012).

51. Álvarez-Lao, D. J. & García, N. Southern dispersal and Palaeoecological implications of woolly rhinoceros (Coelodonta antiquitatis): review of the Iberian occurrences. *Quat. Sci. Rev.* **30**, 2002–2017 (2011).

52. Pochon, Z. *et al.* aMeta: an accurate and memory-efficient ancient metagenomic profiling workflow. *Genome Biol.* **24**, 242 (2023).

53. Guellil, M. *aDNA-BAMPlotter*. (2021). doi:10.5281/zenodo.5702679.

54. Vidal-Cordasco, M., Terlato, G., Ocio, D. & Marín-Arroyo, A. B. Neanderthal coexistence with *Homo sapiens* in Europe was affected by herbivore carrying capacity. *Sci Adv* **9**, eadi4099 (2023).

55. Carvalho, M. *et al.* Initial and lower magdalenian large mammal faunas and human subsistence at El mirón cave (Cantabria, Spain). *J. Paleolit. Archaeol.* **4**, (2021).

56. Straus, L. G., Morales, M. G., Arroyo, A. B. M. & Chiapusso, M. J. I. Las ocupaciones humanas de la cueva del Mirón (Ramales de la Victoria, Cantabria, España) durante el Último Máximo Glacial y el periodo Solutrense. *Espac. Tiempo Forma Ser. Prehist. Arqueol.* 413–426 (2012).

57. Katoh, K. & Standley, D. M. MAFFT multiple sequence alignment software version 7: improvements in performance and usability. *Mol. Biol. Evol.* **30**, 772–780 (2013).

58. Tamura, K., Stecher, G., Peterson, D., Filipski, A. & Kumar, S. MEGA6: Molecular Evolutionary Genetics Analysis version 6.0. *Mol. Biol. Evol.* **30**, 2725–2729 (2013).

59. Rambaut, A. & Drummond, A. FigTree v1. 3.1 Institute of Evolutionary Biology. *Univ. Edinb. J.* (2010).

60. Bianchini, G. & Sánchez-Baracaldo, P. TreeViewer: Flexible, modular software to

visualise and manipulate phylogenetic trees. *Ecol. Evol.* **14**, e10873 (2024).

61. Jones, E. R. *et al.* Upper Palaeolithic genomes reveal deep roots of modern Eurasians. *Nat. Commun.* **6**, 8912 (2015).

62. Yang, M. A. *et al.* 40,000-Year-Old Individual from Asia Provides Insight into Early Population Structure in Eurasia. *Curr. Biol.* **27**, 3202–3208.e9 (2017).

63. Seguin-Orlando, A. *et al.* Paleogenomics. Genomic structure in Europeans dating back at least 36,200 years. *Science* **346**, 1113–1118 (2014).

64. Fu, Q. *et al.* An early modern human from Romania with a recent Neanderthal ancestor. *Nature* **524**, 216–219 (2015).

65. Hajdinjak, M. *et al.* Initial Upper Palaeolithic humans in Europe had recent Neanderthal ancestry. *Nature* **592**, 253–257 (2021).

66. Bollongino, R. *et al.* 2000 years of parallel societies in Stone Age Central Europe. *Science* **342**, 479–481 (2013).

67. Posth, C. *et al.* Pleistocene Mitochondrial Genomes Suggest a Single Major Dispersal of Non-Africans and a Late Glacial Population Turnover in Europe. *Curr. Biol.* **26**, 827–833 (2016).

68. Benazzi, S. *et al.* Archaeology. The makers of the Protoaurignacian and implications for Neandertal extinction. *Science* **348**, 793–796 (2015).

69. Fu, Q. *et al.* A revised timescale for human evolution based on ancient mitochondrial genomes. *Curr. Biol.* **23**, 553–559 (2013).

70. Lazaridis, I. *et al.* Ancient human genomes suggest three ancestral populations for present-day Europeans. *Nature* **513**, 409–413 (2014).

71. Gilbert, M. T. P. *et al.* DNA from pre-Clovis human coprolites in Oregon, North America. *Science* **320**, 786–789 (2008).

72. Günther, T. *et al.* Population genomics of Mesolithic Scandinavia: Investigating

early postglacial migration routes and high-latitude adaptation. *PLoS Biol.* **16**, e2003703 (2018).

73. Vai, S. *et al.* Ancestral mitochondrial N lineage from the Neolithic 'green' Sahara. *Sci. Rep.* **9**, 3530 (2019).

74. Hublin, J.-J. *et al.* Initial Upper Palaeolithic Homo sapiens from Bacho Kiro Cave, Bulgaria. *Nature* 1–4 (2020).

75. Lazaridis, I. *et al.* Paleolithic DNA from the Caucasus reveals core of West Eurasian ancestry. *bioRxiv* 423079 (2018).

76. Gelabert, P. *et al.* Genome-scale sequencing and analysis of human, wolf, and bison DNA from 25,000-year-old sediment. *Curr. Biol.* (2021) doi:10.1016/j.cub.2021.06.023.

77. Villalba-Mouco, V. *et al.* A 23,000-year-old southern Iberian individual links human groups that lived in Western Europe before and after the Last Glacial Maximum. *Nat Ecol Evol* **7**, 597–609 (2023).

78. Briggs, A. W. *et al.* Targeted retrieval and analysis of five Neandertal mtDNA genomes. *Science* **325**, 318–321 (2009).

79. Prüfer, K. *et al.* The complete genome sequence of a Neanderthal from the Altai Mountains. *Nature* **505**, 43–49 (2014).

80. Soares, P. *et al.* The Expansion of mtDNA Haplogroup L3 within and out of Africa. *Mol. Biol. Evol.* **29**, 915–927 (2012).

81. Gonder, M. K., Mortensen, H. M., Reed, F. A., de Sousa, A. & Tishkoff, S. A. Whole-mtDNA genome sequence analysis of ancient African lineages. *Mol. Biol. Evol.* **24**, 757–768 (2007).

82. Torroni, A., Achilli, A., Macaulay, V., Richards, M. & Bandelt, H.-J. Harvesting the fruit of the human mtDNA tree. *Trends Genet.* **22**, 339–345 (2006).

83. Behar, D. M. *et al.* The dawn of human matrilineal diversity. *Am. J. Hum. Genet.* **82**, 1130–1140 (2008).

84. Barbieri, E. & Sestili, P. Reactive oxygen species in skeletal muscle signaling. *J. Signal Transduct.* **2012**, 982794 (2012).

85. Behar, D. M. *et al.* A 'Copernican' reassessment of the human mitochondrial DNA tree from its root. *Am. J. Hum. Genet.* **90**, 675–684 (2012).

86. Harich, N. *et al.* The trans-Saharan slave trade - clues from interpolation analyses and high-resolution characterization of mitochondrial DNA lineages. *BMC Evol. Biol.* **10**, 138 (2010).

87. Cerný, V. *et al.* Migration of Chadic speaking pastoralists within Africa based on population structure of Chad Basin and phylogeography of mitochondrial L3f haplogroup. *BMC Evol. Biol.* **9**, 63 (2009).

88. Batini, C. *et al.* Insights into the demographic history of African Pygmies from complete mitochondrial genomes. *Mol. Biol. Evol.* **28**, 1099–1110 (2011).

89. Ingman, M., Kaessmann, H., Pääbo, S. & Gyllensten, U. Mitochondrial genome variation and the origin of modern humans. *Nature* **408**, 708–713 (2000).

90. Podgorná, E., Soares, P., Pereira, L. & Cerný, V. The genetic impact of the lake chad basin population in North Africa as documented by mitochondrial diversity and internal variation of the L3e5 haplogroup. *Ann. Hum. Genet.* **77**, 513–523 (2013).

91. Vyas, S., Zaganjor, E. & Haigis, M. C. Mitochondria and Cancer. *Cell* **166**, 555–566 (2016).

92. Pennarun, E. *et al.* Divorcing the Late Upper Palaeolithic demographic histories of mtDNA haplogroups M1 and U6 in Africa. *BMC Evol. Biol.* **12**, 234 (2012).

93. Olivieri, A. *et al.* The mtDNA legacy of the Levantine early Upper Palaeolithic in Africa. *Science* **314**, 1767–1770 (2006).

94. González, A. M. *et al.* Mitochondrial lineage M1 traces an early human backflow to Africa. *BMC Genomics* **8**, 223 (2007).
95. Fernandes, V. *et al.* The Arabian cradle: mitochondrial relicts of the first steps along the southern route out of Africa. *Am. J. Hum. Genet.* **90**, 347–355 (2012).
96. Greenspan, B. Family Tree DNA. Preprint at (2008).
97. Derenko, M. *et al.* Complete mitochondrial DNA diversity in Iranians. *PLoS One* **8**, e80673 (2013).
98. Pereira, L. *et al.* No evidence for an mtDNA role in sperm motility: data from complete sequencing of asthenozoospermic males. *Mol. Biol. Evol.* **24**, 868–874 (2007).
99. Achilli, A. *et al.* Mitochondrial DNA backgrounds might modulate diabetes complications rather than T2DM as a whole. *PLoS One* **6**, e21029 (2011).
100. Costa, M. D. *et al.* Data from complete mtDNA sequencing of Tunisian centenarians: testing haplogroup association and the 'golden mean' to longevity. *Mech. Ageing Dev.* **130**, 222–226 (2009).
101. Fraumene, C. *et al.* High resolution analysis and phylogenetic network construction using complete mtDNA sequences in sardinian genetic isolates. *Mol. Biol. Evol.* **23**, 2101–2111 (2006).
102. Derbeneva, O. A., Starikovskaya, E. B., Wallace, D. C. & Sukernik, R. I. Traces of early Eurasians in the Mansi of northwest Siberia revealed by mitochondrial DNA analysis. *Am. J. Hum. Genet.* **70**, 1009–1014 (2002).
103. Thalmann, O. *et al.* Complete mitochondrial genomes of ancient canids suggest a European origin of domestic dogs. *Science* **342**, 871–874 (2013).
104. Loog, L. *et al.* Ancient DNA suggests modern wolves trace their origin to a Late Pleistocene expansion from Beringia. *Mol. Ecol.* **29**, 1596–1610 (2020).

105. Skoglund, P., Ersmark, E., Palkopoulou, E. & Dalén, L. Ancient wolf genome reveals an early divergence of domestic dog ancestors and admixture into high-latitude breeds. *Curr. Biol.* **25**, 1515–1519 (2015).

106. Matsumura, S., Inoshima, Y. & Ishiguro, N. Reconstructing the colonization history of lost wolf lineages by the analysis of the mitochondrial genome. *Mol. Phylogenet. Evol.* **80**, 105–112 (2014).

107. Björnerfeldt, S., Webster, M. T. & Vilà, C. Relaxation of selective constraint on dog mitochondrial DNA following domestication. *Genome Res.* **16**, 990–994 (2006).

108. Chen, L. & Zhang, H. H. Canis lupus chanco mitochondrion, complete genome. Preprint at (2009).

109. Zhang, H., Zhang, J., Chen, L. & Liu, G. The complete mitochondrial genome of Chinese Shinjang wolf. *Mitochondrial DNA* **25**, 106–108 (2014).

110. Pang, J.-F. *et al.* mtDNA data indicate a single origin for dogs south of Yangtze River, less than 16,300 years ago, from numerous wolves. *Mol. Biol. Evol.* **26**, 2849–2864 (2009).

111. Arnason, U., Gullberg, A., Janke, A. & Kullberg, M. Mitogenomic analyses of caniform relationships. *Mol. Phylogenet. Evol.* **45**, 863–874 (2007).

112. Gopalakrishnan, S. *et al.* Interspecific Gene Flow Shaped the Evolution of the Genus Canis. *Curr. Biol.* **28**, 3441–3449.e5 (2018).

113. Bergström, A. *et al.* Grey wolf genomic history reveals a dual ancestry of dogs. *Nature* **607**, 313–320 (2022).

114. Webb, K. M. & Allard, M. W. Mitochondrial genome DNA analysis of the domestic dog: identifying informative SNPs outside of the control region. *J. Forensic Sci.* **54**, 275–288 (2009).

115. Bon, C. *et al.* Coprolites as a source of information on the genome and diet of the

cave hyena. *Proc. Biol. Sci.* **279**, 2825–2830 (2012).

116. Westbury, M. V. *et al.* Hyena paleogenomes reveal a complex evolutionary history of cross-continental gene flow between spotted and cave hyena. *Sci Adv* **6**, eaay0456 (2020).

117. Westbury, M. V. *et al.* Extended and Continuous Decline in Effective Population Size Results in Low Genomic Diversity in the World's Rarest Hyena Species, the Brown Hyena. *Mol. Biol. Evol.* **35**, 1225–1237 (2018).

118. Westbury, M. V., De Cahsan, B., Dalerum, F., Norén, K. & Hofreiter, M. Aardwolf Population Diversity and Phylogenetic Positioning Inferred Using Complete Mitochondrial Genomes. *sawr.1* **49**, 27–33 (2019).

119. Hiendleder, S., Lewalski, H., Wassmuth, R. & Janke, A. The complete mitochondrial DNA sequence of the domestic sheep (Ovis aries) and comparison with the other major ovine haplotype. *J. Mol. Evol.* **47**, 441–448 (1998).

120. Hassanin, A., Ropiquet, A., Couloux, A. & Cruaud, C. Evolution of the mitochondrial genome in mammals living at high altitude: new insights from a study of the tribe Caprini (Bovidae, Antilopinae). *J. Mol. Evol.* **68**, 293–310 (2009).

121. Zhong, H.-M., Zhang, H.-H., Sha, W.-L., Zhang, C.-D. & Chen, Y.-C. Complete Mitochondrial Genome of the Red Fox (Vuples vuples) and Phylogenetic Analysis with Other Canid Species. *Dongwuxue Yanjiu* **31**, 122–130 (2010).

122. Zhang, Z. *et al.* High-Quality Chromosome-Level Genome Assembly of the Corsac Fox (Vulpes corsac) Reveals Adaptation to Semiarid and Harsh Environments. *Int. J. Mol. Sci.* **24**, (2023).

123. Zhao, C., Zhang, H., Liu, G., Yang, X. & Zhang, J. The complete mitochondrial genome of the Tibetan fox (Vulpes ferrilata) and implications for the phylogeny of Canidae. *C. R. Biol.* **339**, 68–77 (2016).

124. Yu, J.-N., Kim, S., Oh, K. & Kwak, M. Complete mitochondiral genome of the Korean red fox Vulpes vulpes (Carnivora, Canidae). *Mitochondrial DNA* **23**, 118–119 (2012).

125. Frank, K. *et al.* Complete mitochondrial genome sequence of a Hungarian red deer (Cervus elaphus hippelaphus) from high-throughput sequencing data and its phylogenetic position within the family Cervidae. *Acta Biol. Hung.* **67**, 133–147 (2016).

126. Kim, H.-J. *et al.* The complete mitochondrial genome of Cervus canadensis (Erxleben, 1777), as a model species of Chronic Wasting Disease (CWD). *Mitochondrial DNA B Resour* **5**, 2621–2623 (2020).

127. Hassanin, A. *et al.* Pattern and timing of diversification of Cetartiodactyla (Mammalia, Laurasiatheria), as revealed by a comprehensive analysis of mitochondrial genomes. *C. R. Biol.* **335**, 32–50 (2012).

128. Rey-Iglesia, A., Grandal-d'Anglade, A., Campos, P. F. & Hansen, A. J. Mitochondrial DNA of pre-last glacial maximum red deer from NW Spain suggests a more complex phylogeographical history for the species. *Ecol. Evol.* **7**, 10690–10700 (2017).

129. Wada, K., Nishibori, M. & Yokohama, M. The complete nucleotide sequence of mitochondrial genome in the Japanese Sika deer (Cervus nippon), and a phylogenetic analysis between Cervidae and Bovidae. *Small Rumin. Res.* **69**, 46–54 (2007).

130. Mackiewicz, P. *et al.* Phylogeny and evolution of the genus Cervus (Cervidae, Mammalia) as revealed by complete mitochondrial genomes. *Sci. Rep.* **12**, 16381 (2022).

131. Li, Y., Ba, H. & Yang, F. Complete mitochondrial genome of Cervus elaphus songaricus (Cetartiodactyla: Cervinae) and a phylogenetic analysis with related

species. *Mitochondrial DNA A DNA Mapp Seq Anal* **27**, 620–621 (2016).

132. Wada, K., Okumura, K., Nishibori, M., Kikkawa, Y. & Yokohama, M. The complete mitochondrial genome of the domestic red deer (Cervus elaphus) of New Zealand and its phylogenic position within the family Cervidae. *Anim. Sci. J.* **81**, 551–557 (2010).
133. Hirata, D. *et al.* Molecular phylogeography of the brown bear (Ursus arctos) in Northeastern Asia based on analyses of complete mitochondrial DNA sequences. *Mol. Biol. Evol.* **30**, 1644–1652 (2013).
134. Miller, W. *et al.* Polar and brown bear genomes reveal ancient admixture and demographic footprints of past climate change. *Proc. Natl. Acad. Sci. U. S. A.* **109**, E2382–90 (2012).
135. Keis, M. *et al.* Complete mitochondrial genomes and a novel spatial genetic method reveal cryptic phylogeographical structure and migration patterns among brown bears in north-western Eurasia. *J. Biogeogr.* **40**, 915–927 (2013).
136. Anijalg, P. *et al.* Ongoing recovery of a brown bear population from a century-old severe bottleneck: insights from population genetic and spatially explicit analyses. *Conserv. Genet.* **21**, 27–40 (2020).
137. Fortes, G. G. *et al.* Ancient DNA reveals differences in behaviour and sociality between brown bears and extinct cave bears. *Mol. Ecol.* **25**, 4907–4918 (2016).
138. Korsten, M. *et al.* Sudden expansion of a single brown bear maternal lineage across northern continental Eurasia after the last ice age: a general demographic model for mammals? *Mol. Ecol.* **18**, 1963–1979 (2009).
139. Delisle, I. & Strobeck, C. Conserved primers for rapid sequencing of the complete mitochondrial genome from carnivores, applied to three species of bears. *Mol. Biol. Evol.* **19**, 357–361 (2002).

140. Krause, J. *et al.* Mitochondrial genomes reveal an explosive radiation of extinct and extant bears near the Miocene-Pliocene boundary. *BMC Evol. Biol.* **8**, 220 (2008).
141. Rey-Iglesia, A. *et al.* Evolutionary history and palaeoecology of brown bear in North-East Siberia re-examined using ancient DNA and stable isotopes from skeletal remains. *Sci. Rep.* **9**, 4462 (2019).
142. Botigué, L. R. *et al.* Ancient European dog genomes reveal continuity since the Early Neolithic. *Nat. Commun.* **8**, 16082 (2017).
143. Bergström, A. *et al.* Origins and genetic legacy of prehistoric dogs. *Science* **370**, 557–564 (2020).
144. Boschin, F. *et al.* The first evidence for Late Pleistocene dogs in Italy. *Sci. Rep.* **10**, 13313 (2020).
145. Achilli, A. *et al.* Mitochondrial genomes from modern horses reveal the major haplogroups that underwent domestication. *Proc. Natl. Acad. Sci. U. S. A.* **109**, 2449–2454 (2012).
146. Xu, S. *et al.* High altitude adaptation and phylogenetic analysis of Tibetan horse based on the mitochondrial genome. *J. Genet. Genomics* **34**, 720–729 (2007).
147. Orlando, L. *et al.* Recalibrating Equus evolution using the genome sequence of an early Middle Pleistocene horse. *Nature* **499**, 74–78 (2013).
148. Lippold, S. *et al.* Discovery of lost diversity of paternal horse lineages using ancient DNA. *Nat. Commun.* **2**, 450 (2011).
149. Der Sarkissian, C. *et al.* Evolutionary Genomics and Conservation of the Endangered Przewalski's Horse. *Curr. Biol.* **25**, 2577–2583 (2015).
150. Librado, P. *et al.* Tracking the origins of Yakutian horses and the genetic basis for their fast adaptation to subarctic environments. *Proc. Natl. Acad. Sci. U. S. A.* **112**, E6889–97 (2015).

151. Yoon, S. H. *et al.* Origin and spread of Thoroughbred racehorses inferred from complete mitochondrial genome sequences: Phylogenomic and Bayesian coalescent perspectives. *PLoS One* **13**, e0203917 (2018).

152. Xu, X., Gullberg, A. & Arnason, U. The complete mitochondrial DNA (mtDNA) of the donkey and mtDNA comparisons among four closely related mammalian species-pairs. *J. Mol. Evol.* **43**, 438–446 (1996).

153. Taron, U. H. *et al.* Ancient DNA from the Asiatic Wild Dog (Cuon alpinus) from Europe. *Genes* **12**, (2021).

154. Campana, M. G. *et al.* Genome sequence, population history, and pelage genetics of the endangered African wild dog (Lycaon pictus). *BMC Genomics* **17**, 1013 (2016).

155. Sosale, M. S. *et al.* The complete mitochondrial genome and phylogenetic characterization of two putative subspecies of golden jackal (Canis aureus cruesemanni and Canis aureus moreotica). *Gene* **866**, 147303 (2023).

156. Koepfli, K.-P. *et al.* Genome-wide Evidence Reveals that African and Eurasian Golden Jackals Are Distinct Species. *Curr. Biol.* **25**, 2158–2165 (2015).

157. Renaud, G., Slon, V., Duggan, A. T. & Kelso, J. Schmutzi: estimation of contamination and endogenous mitochondrial consensus calling for ancient DNA. *Genome Biol.* **16**, 224 (2015).

158. Skoglund, P. *et al.* Genomic insights into the peopling of the Southwest Pacific. *Nature* **538**, 510–513 (2016).

159. Weissensteiner, H. *et al.* HaploGrep 2: mitochondrial haplogroup classification in the era of high-throughput sequencing. *Nucleic Acids Res.* **44**, W58–63 (2016).
