# Supplementary material for: A sedimentary ancient DNA perspective on human and carnivore persistence through the Late Pleistocene in El Mirón Cave, Spain
Source: Nat Commun. 2025 Jan 2;16:107. doi: 10.1038/s41467-024-55740-7 (PMC11696082; doi:10.1038/s41467-024-55740-7)
Supplement: Supplementary file 3 — Description of Additional Supplementary Files [file 41467_2024_55740_MOESM3_ESM.pdf]

Description of additional supplementary files:

Supplementary Data 1: Samples included in the study

Supplementary Data 2: Results of the classification of sequencing reads performed with euka, the taxonomic level is the one determined by euka

Supplementary Data 3: Results of the mapping process against a single reference sequence, the initial reads are those classified by EUKA

Supplementary Data 4: inhibition PCR results

Supplementary Data 5: Preservation of sedaDNA. Description of the A) sequenced reads, B) Classified reads at the species level and C) relative abundance of these reads over the sequenced ones.

Supplementary Data 6: Statistics of the human alignment process including the contamination estimates and variability predictions

Supplementary Data 7: Correspondence between the archaeozoological record and the sedaDNA. The upper value of each cell represents the number of reads attributed to each taxon after the individual mapping process, and the lower value of each cell represents the Number of Identified Specimens (NISP) through faunal analyses. Red represents from 1-500, yellow from 500 to 1000 and green above 1000 units.

Supplementary Data 8: List of recovered partial genomes above 5x coverage

Supplementary Data 9: Description of the variability in the recovered sedaDNA mitogenomes

Supplementary Data 10: Crocuta crocuta pairwise distance matrix perfomed with 100 boostrap replications, 95% of partial deletion and Maximum composite method. Green color has been used to define Haplogroup clusters

Supplementary Data 11: Relevant mtDNA substitutions in reference to NC_012920

Supplementary Data 12: mtDNA used for the mtDNA analyses. Only whole mtDNA sequences have been used.

Supplementary Data 13: Sequences included in the capture designed by TWIST biosciences
